# Imprinted antibody responses against SARS-CoV-2 Omicron sublineages

**DOI:** 10.1126/science.adc9127

**Published:** 2022-10-20

**Authors:** Young-Jun Park, Dora Pinto, Alexandra C. Walls, Zhuoming Liu, Anna De Marco, Fabio Benigni, Fabrizia Zatta, Chiara Silacci-Fregni, Jessica Bassi, Kaitlin R. Sprouse, Amin Addetia, John E. Bowen, Cameron Stewart, Martina Giurdanella, Christian Saliba, Barbara Guarino, Michael A. Schmid, Nicholas M. Franko, Jennifer K. Logue, Ha V. Dang, Kevin Hauser, Julia di Iulio, William Rivera, Gretja Schnell, Anushka Rajesh, Jiayi Zhou, Nisar Farhat, Hannah Kaiser, Martin Montiel-Ruiz, Julia Noack, Florian A. Lempp, Javier Janer, Rana Abdelnabi, Piet Maes, Paolo Ferrari, Alessandro Ceschi, Olivier Giannini, Guilherme Dias de Melo, Lauriane Kergoat, Hervé Bourhy, Johan Neyts, Leah Soriaga, Lisa A. Purcell, Gyorgy Snell, Sean P.J. Whelan, Antonio Lanzavecchia, Herbert W. Virgin, Luca Piccoli, Helen Y. Chu, Matteo Samuele Pizzuto, Davide Corti, David Veesler

**Affiliations:** 1 Department of Biochemistry, University of Washington, Seattle, WA 98195, USA.; 2 Howard Hughes Medical Institute, University of Washington, Seattle, WA 98195, USA.; 3 Humabs Biomed SA, a subsidiary of Vir Biotechnology, 6500 Bellinzona, Switzerland.; 4 Department of Molecular Microbiology, Washington University School of Medicine, St. Louis, MO 63110, USA; 5 Division of Allergy and Infectious Diseases, University of Washington, Seattle, WA 98195, USA.; 6 Vir Biotechnology, San Francisco, CA 94158, USA; 7 KU Leuven Department of Microbiology, Immunology and Transplantation, Rega Institute for Medical Research, Laboratory of Virology and Chemotherapy, B-3000 Leuven, Belgium; 8 Faculty of Biomedical Sciences, Università della Svizzera italiana, Lugano, Switzerland.; 9 Division of Nephrology, Ente Ospedaliero Cantonale, Lugano, Switzerland.; 10 Clinical School, University of New South Wales, Sydney, New South Wales, Australia.; 11 Clinical Trial Unit, Ente Ospedaliero Cantonale, Lugano, Switzerland.; 12 Division of Clinical Pharmacology and Toxicology, Institute of Pharmacological Sciences of Southern Switzerland, Ente Ospedaliero Cantonale, Lugano, Switzerland.; 13 Department of Clinical Pharmacology and Toxicology, University Hospital Zurich, Zurich, Switzerland; 14 Department of Medicine, Ente Ospedaliero Cantonale, Bellinzona, Switzerland; 15 Institut Pasteur, Université de Paris Cité, Lyssavirus Epidemiology and Neuropathology Unit, Paris, F-75015, France; 16 Department of Pathology and Immunology, Washington University School of Medicine, St Louis, MO, USA; 17 Department of Internal Medicine, UT Southwestern Medical Center, Dallas, TX, USA

## Abstract

SARS-CoV-2 Omicron sublineages carry distinct spike mutations and represent an antigenic shift resulting in escape from antibodies induced by previous infection or vaccination. We show that hybrid immunity or vaccine boosters elicit plasma neutralizing activity against Omicron BA.1, BA.2, BA.2.12.1 and BA.4/5 and that breakthrough infections, but not vaccination-only, induce neutralizing activity in the nasal mucosa. Consistent with immunological imprinting, most antibodies derived from memory B cells or plasma cells of Omicron breakthrough cases cross-react with the Wuhan-Hu-1, BA.1, BA.2, and BA.4/5 receptor-binding domains whereas Omicron primary infections elicit B cells of narrow specificity up to 6 months post infection. Although most clinical antibodies have reduced neutralization of Omicron, we identified an ultrapotent pan-variant neutralizing antibody, that is a strong candidate for clinical development.

The emergence of SARS-CoV-2 Omicron at the end of 2021 caused worldwide COVID-19 case surges. The Omicron BA.1 and BA.1.1 lineages swept the world followed by the BA.2 lineage (1). Although BA.1 and BA.2 share a large number of spike (S) mutations, they are each characterized by unique sets of amino acid changes, which are associated with different antigenic properties (2–4). The BA.2.12.1 sublineage emerged in the United States, peaking at the beginning of June and is characterized by the presence of the S_1_ L452Q receptor-binding domain (RBD) and S704L S_2_ subunit mutations in addition to the BA.2-defining mutations (4). The BA.2.75 sublineage is spreading in multiple countries and carries unique mutations (added to the BA.2 background) in the N-terminal domain (NTD), along with D339H, G446S and N460K mutations and the R493Q reversion in the RBD (5). The BA.3 S glycoprotein comprises a combination of mutations found in BA.1 S and BA.2 S (6), whereas BA.4 S and BA.5 S are identical to each other and comprise a deletion of residues 69–70, the L452R and F486V substitutions along with the R493Q reversion compared to BA.2 S (7). We characterized the emergence of Omicron (BA.1) as a major antigenic shift due to the unprecedented magnitude of immune evasion associated with this variant of concern (3, 8–12). Mutations in the BA.1 S glycoprotein NTD and RBD, which are the main targets of neutralizing antibodies (3, 8, 13–18), explain the markedly reduced plasma neutralizing activity of previously infected or vaccinated subjects, especially those that have not received booster doses, and the escape from most monoclonal antibodies (mAbs) used in the clinic. As a result, an increasing number of reinfections or breakthrough infections are occurring (19–22), even though these cases tend to be milder than infections of immunologically naive individuals.

## Characterization of plasma and mucosal humoral responses to Omicron infection

**Figure 1.**
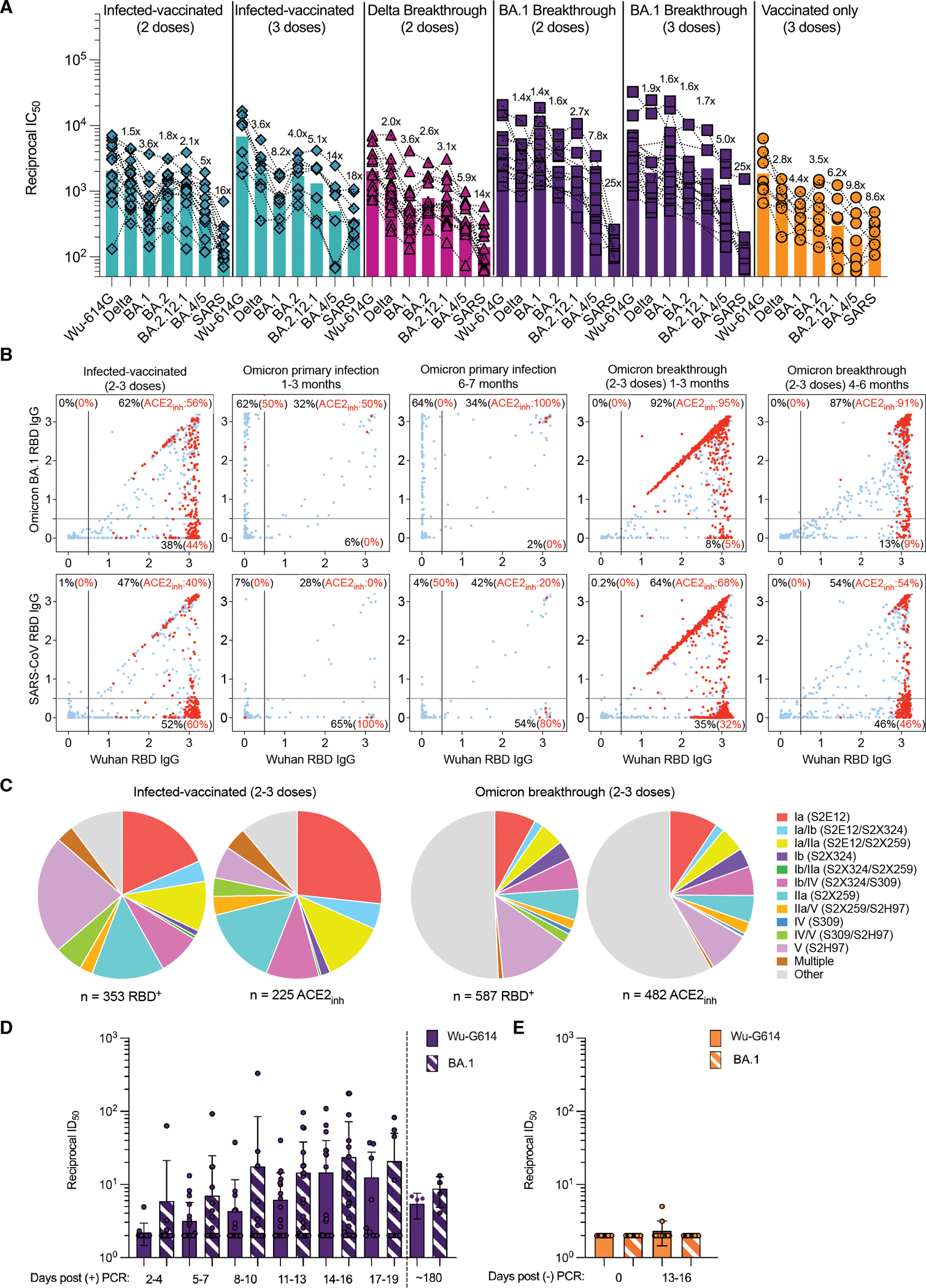
Evaluation of plasma, memory and mucosal antibody responses upon Omicron breakthrough infections in humans. **A**, Pairwise neutralizing activity (half-maximum inhibitory dilution; ID_50_) against Wu-G614, Delta, BA.1, BA.2, BA.2.12.1, BA.4/5 and SARS-CoV S VSV pseudoviruses using plasma from subjects who were infected and vaccinated, vaccinated and experienced breakthrough infection, or vaccinated-only individuals. VeroE6-TMPRSS2 cells were used as target cells (93). Data are the geometric mean of an *n* = 2 technical replicate and have been performed in at least 2 biologically independent experiments. GMTs are shown with a color-matched bar (and reported in Table S1) with fold change compared to Wu-G614 indicated above it. Demographics of enrolled donors are provided in Data S1. **B**, Cross-reactivity of IgGs secreted from memory B cells obtained from infected-vaccinated individuals (n=11), primary SARS-CoV-2 infection (n=3 samples collected at 1–3 months and n=2 samples collected at 6–7 months) or breakthrough cases (n=7 samples collected at 1–3 months and n=4 samples collected at 4–6 months) occurring in January-March 2022 when the prevalence of Omicron BA.1/BA.2 exceeded 90% in the region where samples were obtained (Fig. S2). Each dot represents a well containing oligoclonal B cell supernatant screened for the presence of IgGs binding to the SARS-CoV-2 Wuhan-Hu-1 and BA.1 RBDs (top) or to the SARS-CoV-2 Wuhan-Hu-1 and SARS-CoV RBDs (bottom) using ELISA. Red dots indicate inhibition of the interaction with ACE2 (using Wuhan-Hu-1 target antigen) as determined in a separate assay. The percentages are expressed relative to the total of positive hits against any of the antigens tested. Numbers of positive hits relative to individual donors are shown in Fig. S3. **C**, Frequency analysis of site-specific IgG antibodies derived from memory B cells. RBD sites targeted by IgG derived from memory B cells were defined by a blockade-of-binding assay using mAbs specific for sites Ia (S2E12), Ib (S2X324), IIa (S2X259), IV (S309; parent of sotrovimab) and V (S2H97). Hybrid sites Ia/Ib, Ia/IIa, Ib/IIa, Ib/IV, IIa/V and IV/V were defined by competition with the two corresponding mAbs. Hybrid sites exhibiting competition with more than 2 mAbs are indicated as “Multiple”. Lack of competition is indicated as “Other”. Pie charts show cumulative frequencies of IgGs specific for the different sites among total RBD-directed IgG antibodies (left) and those inhibiting binding of RBD to human ACE2 (right) in 11 infected-vaccinated individuals or 7 breakthrough cases. **D**, Neutralizing activity against Wu-G614 and BA.1 S VSV pseudoviruses determined from nasal swabs obtained longitudinally upon BA.1 breakthrough infection up to 185 days following positive PCR test (post (+) PCR). **E**, Neutralizing activity against Wu-G614 and BA.1 S VSV pseudoviruses from nasal swabs obtained longitudinally following a negative PCR (post (−) PCR)_ test of vaccinated-only individuals.

Understanding the relationships between prior antigen exposure, through vaccination or infection with one SARS-CoV-2 strain, and the immune response to subsequent infections with a different strain is paramount to guiding strategies to exit the COVID-19 pandemic. To address this question, we first evaluated the magnitude of immune evasion associated with the Omicron sublineages by assessing the neutralizing activity of human plasma using a non-replicative vesicular stomatitis virus (VSV) pseudotyped with Wuhan-Hu-1 S harboring G614 (Wu-G614), Delta, BA.1, BA.2, BA.2.12.1 and BA.4/5 mutations or with SARS-CoV S (Fig. 1A, Fig. S1A–G, Table S1, Data S1). We compared plasma from 6 cohorts of individuals: those previously infected in 2020 (with a Washington-1-like SARS-CoV-2 strain) and then vaccinated twice or three times (‘Infected-vaccinated 2 doses’, ‘Infected-vaccinated 3 doses’), those who were vaccinated and then experienced either a Delta or an Omicron BA.1 breakthrough infection (‘Delta breakthrough 3 doses’, ‘BA.1 breakthrough 2 doses’ or ‘BA.1 breakthrough 3 doses’), or those who have only been vaccinated and boosted (‘vaccinated-only 3 doses’). Neutralizing antibody responses were slightly more robust against BA.2 S VSV than BA.1 S VSV among all groups except for the BA.1 breakthrough cases. Reductions of geometric mean titers (GMTs) relative to Wu-G614 S VSV ranged from 1.4- and 8.2-fold against BA.1 and between 1.6- and 4-fold against BA.2 (Fig. 1A, Fig. S1A–G, Table S1, Data S1), in line with recent findings (4). BA.2.12.1 S VSV was associated with further reductions of plasma neutralizing activity relative to BA.2 S VSV whereas BA.4/5 S VSV had the greatest impact of all SARS-CoV-2 variants evaluated here with GMT reductions of 5- to 14-fold relative to Wu-G614 S VSV (Fig. 1A, Fig. S1A–G, Table S1, Data S1). All six cohorts experienced reductions in plasma neutralizing GMT of 1.4–3.6-fold against Delta (23–25) relative to Wu-G614 S VSV, underscoring that even hybrid immunity (i.e., acquired through vaccination and infection (26)) do not overcome evasion from neutralizing antibody responses of this previously dominant variant of concern (Fig. 1A, Fig. S1A–G, Table S1, Data S1). The highest neutralizing GMTs against SARS-CoV-2 variants were observed for BA.1 breakthrough cases, possibly due to exposure to BA.1 S, as no correlation was found between time intervals and GMTs (Data S1). Neutralizing GMTs against the SARS-CoV S pseudovirus was reduced for all cohorts by 8.6- to 25-fold relative to Wu-G614 S VSV, underscoring the marked genetic and antigenic divergence of this sarbecovirus clade (19, 27, 28).

Given the recall of Wuhan-Hu-1 plasma neutralizing antibodies in Omicron breakthrough cases, we investigated the cross-reactivity of RBD-directed antibodies produced by in vitro stimulated memory B cells obtained up to 200 days after infection or vaccination as well as circulating plasma cells collected in the days following infection (29). These analyses used blood samples from individuals who were infected prior to the emergence of Omicron and subsequently vaccinated (‘Infected-vaccinated 2/3 doses’) as well as subjects who experienced either an Omicron primary infection or an Omicron breakthrough infection. Primary and breakthrough Omicron infections occurred between January and March 2022 during which the prevalence of Omicron BA.1/BA.2 sublineages exceeded 90% in the region from which samples were obtained (Fig. S2). Plasma neutralizing activity of Omicron-infected (primary and breakthrough) cases was reduced on average 6.1-fold against BA.4/BA.5 S VSV relative to BA.1 S VSV (Table S2), likely as a result of both RBD and NTD mutations in the former lineage, concurring with the above data and recent studies (30, 31). Strikingly, more than 80% of SARS-CoV-2 Wuhan-Hu-1 RBD-directed IgGs secreted by memory B cells and plasma cells obtained from breakthrough cases cross-reacted with BA.1, BA.2, BA.4/5 and Delta RBDs, and more than 90% of these antibodies blocked binding to ACE2 (a correlate of neutralization (13, 32)) (Fig. 1B, Fig. S3–S6, Table S2). Moreover, Omicron breakthrough infections failed to elicit BA.1-, BA.2- or BA.4/5-specific RBD-directed memory B cells. Notably, a fraction of Wuhan-Hu-1 RBD-directed antibodies cross-reacted with BA.2, but not BA.1 RBD (7 to 9%), and a smaller fraction (1 to 3%) also cross-reacted with BA.4/5 RBD, consistent with the antigenic distance of BA.1 from the other Omicron sublineages (Fig. 1B, Fig. S3–S6, Table S2). Furthermore, the proportion of BA.4/5-reacting antibodies cross-reacting with Wuhan-Hu-1, BA.1 and BA.2 decreased overtime when comparing 1–3 versus 4–6 months after breakthrough infections (Fig. S4D–F). This suggests that the maturation of antibodies driven by BA.1 or BA.2 breakthrough infections may also result in narrowing their specificity over time, thereby decreasing cross-reactivity with the BA.4/5 RBD. These findings illustrate how immunological imprinting from prior exposure, also named ‘original antigenic sin’, can strongly affect the response to distantly related antigens. In contrast, memory B cell-derived RBD-directed IgG antibodies obtained from Omicron primary infections up to 6–7 months after infection were present at low frequency and were mostly specific for the BA.1 and BA.2 RBDs, but did not cross-react with the BA.4/5 RBD (Fig. 1B, Fig. S3–S6, Data S1). The frequency of IgG antibodies cross-reacting with the SARS-CoV RBD was similar across all three cohorts, concurring with the overall weak plasma neutralizing activity (Fig. 1A–B and Table S2).

We determined the site specificity of RBD-directed antibodies secreted by stimulated memory B cells by competition with structurally characterized mAbs targeting four distinct antigenic sites (13, 27). Most of the memory B cell-derived antibodies from (pre-Omicron) infected-vaccinated individuals competed with the five reference mAbs used whereas a large fraction of antibodies from Omicron breakthrough cases did not compete with any of these five mAbs, indicating they recognize other undefined RBD antigenic sites (Fig. 1C and Fig. S7). Antibodies recognizing most antigenic sites overlapping with the receptor-binding motif, such as mAb S2E12 (33), were found at lower frequency upon Omicron breakthrough infections relative to infected-vaccinated subjects, consistent with the presence of several immune escape mutations in the Omicron RBM (Fig. 1C and Fig. S7) (3, 18). A similar relative reduction was observed for antibodies targeting RBD antigenic site IIa (recognized by the S2X259 mAb (34)) (Fig. 1C and Fig. S7), in agreement with previous findings describing Omicron immune escape from several site IIa mAbs (3, 8, 18). Collectively, these findings demonstrate that Omicron breakthrough infections preferentially expanded existing B cell pools primed by vaccination and elicited cross-reactive plasma cells and antibodies, supporting the concept of immunological imprinting.

To evaluate mucosal antibody responses in subjects who experienced a BA.1 breakthrough infection or vaccinated-only individuals, we assessed IgG and IgA binding titers in nasal swabs obtained longitudinally after PCR testing. Although we detected S-specific IgG, and to a lesser extent IgA, in swabs from several breakthrough cases, vaccinated-only individuals had no detectable binding antibody titers (Fig. S8A–D, Fig. S9A–B). We observed mucosal neutralizing activity against Wu-G614 and BA.1 S VSV pseudoviruses for nasal swabs obtained from breakthrough cases throughout the month following symptoms onset, corresponding to up to 19 days post positive PCR testing (Fig. 1D–E, fig. S9C and Data S1). Furthermore, analysis of nasal swabs obtained from four breakthrough cases approximately six months after symptoms onset demonstrated retention of neutralizing activity. Assessing plasma neutralizing antibody titers of these BA.1 breakthrough cases yielded similar magnitude and GMT reductions compared to the rest of the BA.1 breakthrough cohort (Fig. 1A, Figure S1F and Data S1). We note that the magnitude of neutralizing antibody responses in nasal swabs cannot be directly compared to plasma samples due to the self-administration procedure and resulting sample non-uniformity. Overall, we observed heterogenous mucosal neutralizing antibody responses among BA.1 breakthrough cases but not in vaccinated-only individuals (Fig. 1D–E, fig. S9C–D and Data S1). Collectively, these data underscore the lack of or very weak induction of mucosal antibody responses upon intra-muscular delivery of mRNA vaccines or adenovirus-vectored vaccines (35, 36) and are in line with concurrent findings that Omicron breakthrough infection but not vaccination alone induced neutralizing antibody responses and tissue-resident T cells in the nasal mucosa (37, 38).

## Omicron sublineages escape neutralization mediated by most clinical mAbs

**Figure 2:**
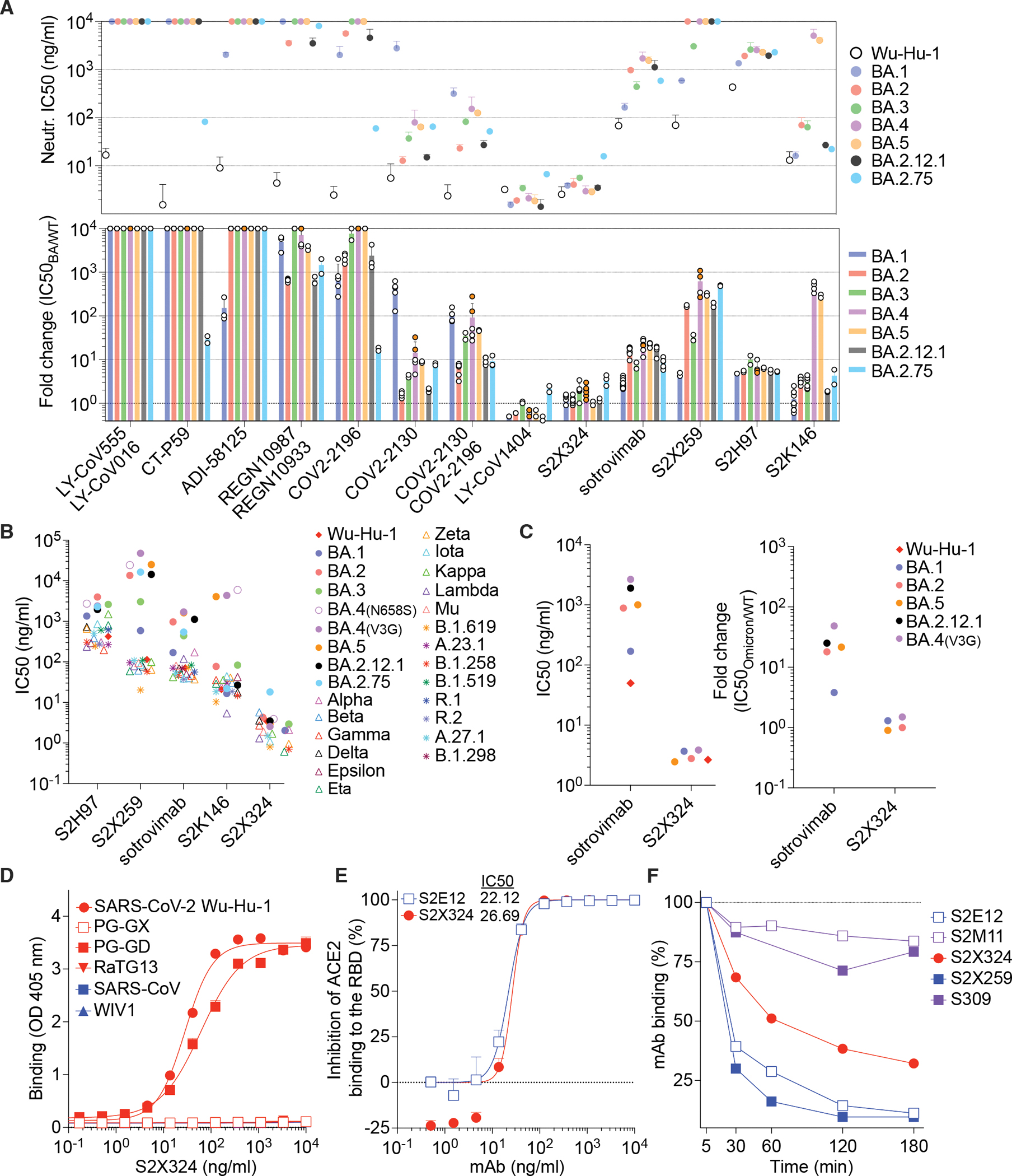
Identification and characterization of S2X324 as a pan-variant RBD-directed mAb. **(A)** mAb-mediated neutralization of BA.1, BA.2, BA.3, BA.4, BA.5, BA.2.12.1, and BA.2.75 S VSV pseudoviruses. Two haplotypes of BA.4 S were tested: BA.4-V3G (orange dots) and BA.4-N658S (white dots) and the IC_50_ values reported in the text are the averages of both haplotypes. The potency of each mAb or mAb cocktail is represented by their IC_50_ (top, geometric mean ± SD) or fold change relative to neutralization of the Wuhan-Hu-1 (D614) pseudovirus (bottom, average ± SD). *, not determined. **(B)** Neutralization of SARS-CoV-2 variant S VSV pseudoviruses mediated by broadly neutralizing sarbecovirus mAbs. Each symbol represents the GMT of at least two independent experiments. **(C)** Neutralizing activity (left) and fold change relative to WA-1/2020 (right) of S2X324 and sotrovimab against SARS-CoV2 Omicron BA.1, BA.2, BA.4, BA.5, and BA.2.12.1 authentic viruses using VeroE6-TMPRSS2 target cells. Data are representative of at least 2 biological independent experiments. Neutralization of Omicron BA.1 by sotrovimab refers to previously published data (3). **(D)** Cross-reactivity of S2X324 with sarbecovirus clade 1a and 1b RBDs analyzed by ELISA. (**E**) Preincubation of serial dilutions of S2X324 or S2E12 with the SARS-CoV-2 RBD prevents binding to the immobilized human ACE2 ectodomain in ELISA. PG-GX: Pangolin-Guangxi, PG-GD: Pangolin-Guangdong. Error bars indicate standard deviation between replicates. (**F**) S2X324-mediated S_1_-shedding from cell surface–expressed SARS-CoV-2 S as determined by flow cytometry. S2E12 and S2X259 were used as positive controls whereas S2M11 and S309 were used as negative controls.

We next evaluated the impact of BA.1, BA.2, BA.3, BA.4, BA.5, BA.2.12.1 and BA.2.75 S mutations on neutralization mediated by a panel of RBD-directed mAbs using VSV pseudoviruses and VeroE6 target cells. The site Ib COV2–2130 mAb weakly neutralized BA.1 (3), while it neutralized BA.2, BA.3, BA.4, BA.5, BA.2.12.1 and BA.2.75 S VSV pseudoviruses with 1.6-fold, 4.2-fold, 14.5-fold, 8.8-fold, 2.0-fold and 7.9-fold respective drops in half-maximal inhibition concentrations (IC_50_) compared to Wu-G614 S VSV (Fig. 2A and fig. S10A–B). Moreover, the COV2–2196+COV2–2130 mAb cocktail experienced a 106.4-fold, 7.6-fold, 35-fold, 92.8-fold, 46.5-fold, 9.3-fold and 9.1-fold reduction in potency against BA.1, BA.2, BA.3, BA.4, BA.5, BA.2.12.1 and BA.2.75, respectively (Fig. 2A and fig. S10A–B). Since COV2–2196 weakly inhibited Omicron sublineages (except for BA.2.75 where the reduction in IC_50_ is 17.3-fold), the neutralizing activity of the cocktail is largely mediated by COV2–2130. Within the COV2–2130 epitope, position 446 is a glycine residue for Wuhan-Hu-1, BA.2, BA.4, BA.5 and BA.2.12.1 S or a serine residue in BA.1, BA.3 and BA.2.75 S, the latter residue disrupting the binding interface of COV2–2130 (18). The importance of this site was also identified through deep mutational scanning (39) and this point mutation was shown to reduce neutralizing activity ~4 fold for COV2–2130 (8). The greater reduction in potency against BA.4 and BA.5 relative to BA.2 is likely driven by the L452R mutation, as reported (https://www.fda.gov/media/154701/download) (39). The REGN10987+REGN10933 mAb cocktail, LY-CoV16+LY-CoV555 mAb cocktail, the CT-P59 mAb and ADI-58125 mAb experienced reductions of in vitro neutralization potency ranging between two and four orders of magnitude against all Omicron sublineage S VSV pseudoviruses compared to Wu-G614 S VSV due to mutations in the RBM (Fig. 2A and fig. S10A–B) (18). CT-P59, however, retained neutralizing activity against the BA.2.75 sublineage (29.2-fold reduction relative to Wu-G614 S VSV). The recently described ACE2-mimicking S2K146 mAb (40) that retained unaltered activity against BA.1 compared to Wu-G614 (3), had a mildly reduced neutralizing activity against BA.2, BA.3, BA.2.12.1 and BA.2.75 S VSV pseudoviruses (3.3-fold, 3.1-fold, 1.9-fold and 4.3-fold, respectively) (Fig. 2A and fig. S10A–B). However, S2K146 experienced a marked reduction in neutralizing activity against BA.4 and BA.5 (with 472- and 285-fold IC_50_ reductions compared to Wu-G614 S VSV), likely due to the F486V mutation.

Sotrovimab, a site IV mAb with broad sarbecovirus (clade Ia and Ib) cross-neutralizing activity (41), experienced 16-fold, 7.3-fold, 21.3-fold, 22.6-fold, 16.6-fold and 8.3-fold reduction in potency relative to Wu-G614 against BA.2, BA.3, BA.4, BA.5, BA.2.12.1 and BA.2.75, respectively (Fig. 2A and fig. S10A–B). These reductions in neutralizing activity are greater than that observed against BA.1 (2.7-fold), although no additional residue mutations map to the sotrovimab epitope except the G339H substitution present in BA.2.75 instead of G339D found in BA.1 (41–43). We recently showed that sotrovimab retained in vitro effector functions against BA.2 and conferred Fc-dependent protection in the lungs of mice infected with BA.2 (44). The additional loss of neutralization of these Omicron sublineage VSV pseudoviruses beyond BA.1 likely results from the S371F substitution, which is found in BA.2, BA.3, BA.4/5, BA.2.12.1 and BA.2.75 and introduces a bulky phenylalanine nearby the N343 glycan which is part of the sotrovimab epitope (41). A recently determined BA.2 S structure shows that the RBD helix comprising residues 364–372 is indeed remodeled (45) and adopts a distinct conformation than the ones observed for Wuhan-Hu-1 S or BA.1 S structures (18, 46). This structural rearrangement is sterically incompatible with the glycan N343 conformation observed in S309-bound spike structures (18, 41), as supported by molecular dynamics simulations and likely explains the reductions in neutralization potency (fig. S11A–D). Although we could not test the effect of the S371F substitution alone in the Wu-G614 S background (due to poor VSV pseudovirus infectivity), the S371F, S373P and S375F triple mutant (as found in BA.2, BA.3, BA.4, BA.5, BA.2.12.1 and BA.2.75) reduced sotrovimab-mediated neutralization by 3.4-fold relative to Wu-D614 S VSV (Fig. S11E and Table S3). Moreover, the S371L, S373P and S375F triple mutant (as found in BA.1) did not alter sotrovimab activity (Fig. S11F and Table S3), lending further support to the role of F371 in reducing the sotrovimab potency against BA.2, BA.3, BA.4, BA.5, BA.2.12.1 and BA.2.75.

S2X259, a site IIa mAb that broadly reacts with the RBD of multiple sarbecoviruses (34) retained activity against BA.1 (3). However, the neutralization potency of S2X259 was decreased by one to two orders of magnitude against BA.2, BA.3, BA.4, BA.5, BA.2.12.1 and BA.2.75 S VSV pseudoviruses (Fig. 2A and fig. S10A–B), likely due to the detrimental effect of the aforementioned S371F/S373P/S375F-induced remodeling and of the R408S mutation (34). S2H97 is a site V mAb that experienced a 4.7- to 10-fold decrease in neutralization potency against Omicron sublineages compared to Wu-G614 S VSV (Fig. 2A and fig. S10A–B) despite the absence of mutations present in the epitope or otherwise found to affect binding by DMS, perhaps reflecting differential accessibility to its cryptic epitope in the context of these S trimers (27).

## Identification of the pan-variant and ultrapotent neutralizing mAb S2X324

The S2X324 mAb stood out in our panel as its neutralization potency was largely unaffected by BA.1, BA.2, BA.3, BA.4, BA.5, BA.2.12.1 and BA.2.75 S mutations (Fig. 2A and fig. S10A–B). S2X324 cross-reacted with and neutralized all SARS-CoV-2 (VSV pseudovirus and authentic virus) variants tested with IC_50_ values below 10 ng/ml except BA.2.75 for which the IC_50_ was 18 ng/ml (Fig. 2B–C, fig. S10A–C, fig. S12, fig. S13 and Table S4). S2X324 cross-reacted with the sarbecovirus clade 1b Pangolin-GD RBD, but did not recognize more divergent sarbecovirus RBDs (Fig. 2D), in contrast to the previously described broadly neutralizing mAbs S2X259 (34). Furthermore, S2X324 inhibited binding of the SARS-CoV-2 RBD to human ACE2 in a concentration-dependent manner, as measured by competition ELISA (Fig. 2E) and induced slow premature shedding (47) of the S_1_ subunit from cell-surface expressed S (Fig. 2F). However, S2X324 did not promote the fusogenic conformational changes of a wildtype-like purified recombinant S ectodomain trimer (Fig. S14), likely due to the slow kinetics of S_1_ shedding. This suggests that blockage of ACE2 binding is the main mechanism of S2X324-mediated inhibition of SARS-CoV-2.

To evaluate the ability of S2X324 to promote antibody dependent-phagocytosis or cytotoxicity, we tested whether the mAb could activate Fcγ receptors expressed at the surface of Jurkat cells. Although S2X324 only activated FcγRIIIa but not FcγRIIa in vitro (fig. S15 A–B), it triggered both antibody-dependent phagocytosis and cytotoxicity following incubation of peripheral blood mononuclear cells with SARS-CoV-2 S-expressing cells (Fig. S15 C–F). The slow S_1_ shedding kinetics likely explain the ability of S2X324 to promote Fc-mediated effector functions.

## Structural basis for S2X324-mediated neutralization

**Figure 3:**
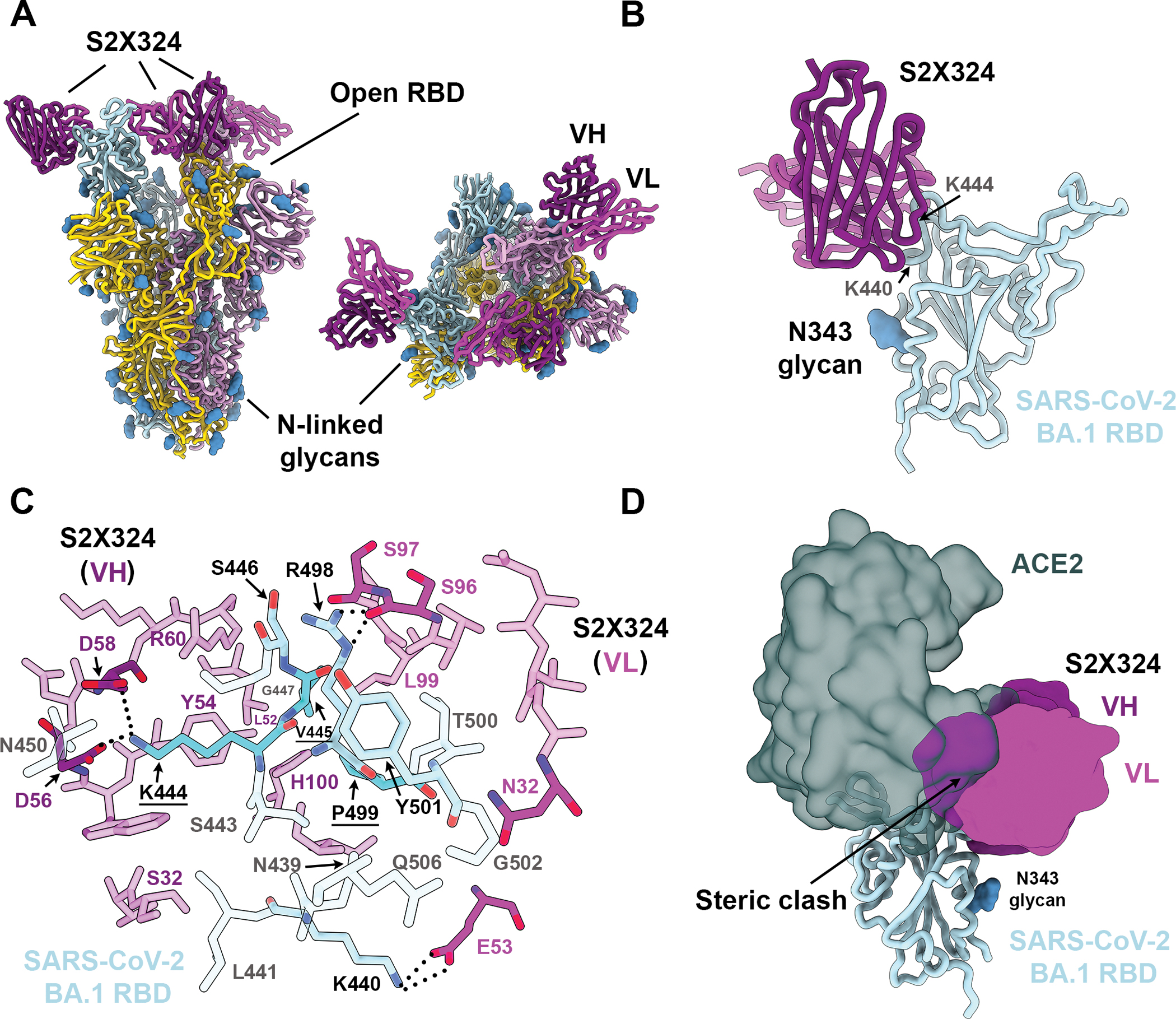
Structural characterization of the S2X324 pan-variant mAb. (**A**) Cryo-EM structure viewed along two orthogonal orientations of the prefusion SARS-CoV-2 Omicron BA.1 S ectodomain trimer with three S2X324 Fab fragments bound. SARS-CoV-2 S protomers are colored light blue, pink, and gold. S2X324 heavy chain and light chain variable domains are colored purple and magenta, respectively. Glycans are rendered as blue spheres. (**B**) Ribbon diagram of the S2X324-bound SARS-CoV-2 RBD. The N343 glycan is rendered as blue spheres. (**C**) Zoomed-in view of the contacts formed between S2X324 and the SARS-CoV-2 BA.1 RBD. Selected epitope residues are labeled, and electrostatic interactions are indicated with dotted lines. A few of the escape mutants identified are colored turquoise. (**D**) Superimposition of the S2X324 -bound (purple and magenta) and ACE2-bound [dark gray, PDB 6M0J (94)] SARS-CoV-2 RBD (light blue) structures showing steric overlap. The N343 glycan is rendered as blue spheres.

To understand the pan-variant S2X324 inhibitory activity, we determined a cryo-electron microscopy structure of the Omicron BA.1 S ectodomain trimer bound to the S2X324 Fab fragment at 3.1 Å resolution (Fig. 3A, Fig S16 and Table S5). In our structure, the BA.1 S trimer has three Fabs bound to one closed and two open RBDs. We used focused classification and local refinement of the closed RBD-S2X324 Fab complex to obtain a 3.3 Å structure revealing the molecular details of the binding interface.

S2X324 recognizes an RBD epitope partially overlapping with antigenic sites Ib and IV (Fig. 3A–B), explaining the observed competition with S2H14 (13) and S309 (sotrovimab parent) (41) mAbs (fig. S13B). S2X324 utilizes all six complementary-determining loops to recognize RBD residues T345, N439, K440, L441, S443, K444, V445, S446, G447, N448, Y449, N450, R498, P499, T500, Y501, G502, Q506 and R509 (Fig. 3C). In line with the competition assay, S2X324 overlaps with the receptor-binding motif on the RBD and sterically hinders receptor engagement (Fig 2E and Fig. 3D).

The structure explains how this mAb accommodates residues that are mutated in Omicron lineages relative to Wuhan-Hu-1: N440K (BA.1/BA.2/BA.3/BA.4/BA.5/BA.2.12.1/BA.2.75), G446S (BA.1/BA.3//BA.2.75), Q498R (BA.1/BA.2/BA.3/BA.4/BA.5/BA.2.12.1/BA.2.75) and N501Y (BA.1/BA.2/BA.3/BA.4/BA.5/BA.2.12.1/BA.2.75). Specifically, K440 forms a salt bridge with the VL E53 side chain, S446 forms van der Waals interactions with VH R60 and VL S96/S97 whereas R498 forms electrostatic interactions with the VL S96 backbone. Our structure further suggests that the tighter binding of S2X324 to the Wuhan-Hu-1 and BA.2 RBDs, relative to BA.1 (Fig. S13A), might be due to G446S as although the mutation is clearly accommodated, at least 1 out of 3 favored rotamers for S446 would clash with the Fab. The Y501 backbone forms van der Waals interactions with the VL N32 side chain which are independent of the RBD residue identity at position 501 (explaining retention of neutralization of all Y501-containing variants). S2X324 and LY-CoV1404 share 87% and 91% amino acid sequence identity in their heavy and light chains, respectively, likely explaining their similar binding mode (Fig. S17) (48), pan-variant neutralizing activity (49) and their comparable resilience to Omicron sublineage mutations thus far (Fig 2A).

## Identification of S2X324 viral escape mutants in vitro

To explore potential mutations that could promote escape from S2X324-mediated neutralization, we passaged a replication-competent VSV chimera harboring either SARS-CoV-2 Wu-G614 S (50) or Omicron BA.1 S in the presence of S2X324. Residue substitutions at three distinct sites emerged in both S backgrounds (Fig. 3C, Fig. S18A–B and Tables S6–S7): (i) K444N/T (Wu-G614 and BA.1 background) and K444E/M (BA.1 background), that would abrogate the salt bridges formed between the K444 side chain and the heavy chain D56 and D58 side chains; (ii) V445D (Wu-G614 background) and V445A/F (BA.1 background), which would disrupt Van der Waals contacts with S2X324; and (iii) P499R (Wu-G614 background) and P499S/H (BA.1 background) that might alter the local RBD backbone conformation and/or sterically hinder mAb binding. Furthermore, three additional mutations were detected in the BA.1 S background only: S446I, G447S, and N448K, which are positioned near the interface between the heavy and light chains (Fig. 3C, Fig. S18A–B and Tables S6–S7). The VSV chimera harboring SARS-CoV-2 Wu-G614 S outcompeted the chimeras harboring the K444T/N, V445D or P499R escape mutants after four rounds of passaging, suggesting reduced fitness in this replicating chimeric virus model system (Fig. S18C). Even though each of these mutations require a single nucleotide substitution, they are very rare and have been detected cumulatively only in 0.087% and 0.080% of Delta and Omicron genome sequences as of August 12^th^ 2022, respectively (Table S8 and Fig. S19) albeit the frequency of some of them is increasing. We further tested VSV pseudoviruses bearing Wu-G614, BA.1 or BA.2 S carrying K444E, K444D, K444N, K444T, V445D, and P449R/H and confirmed that these mutations abrogated or strongly reduced S2X324 neutralizing activity (Fig. S19 and Table S9). In addition, S2X324 neutralizing activity was abrogated when V445T/A/F was introduced in the BA.1 backbone (Table S9). S2X324 retained potent neutralizing activity against pseudoviruses bearing other mutations in the epitope found in known variants such as N439K, N440K and N501Y in the Wu-G614 S background (Table S9). Although the S2X324 escape mutants identified are rare, these data suggest that a mAb cocktail comprising S2X324 would increase the barrier for the emergence of resistance mutants even further compared to this single mAb.

## S2X324 protects hamsters against SARS-CoV-2 Delta, BA.2 and BA.5 variants

**Figure 4.**
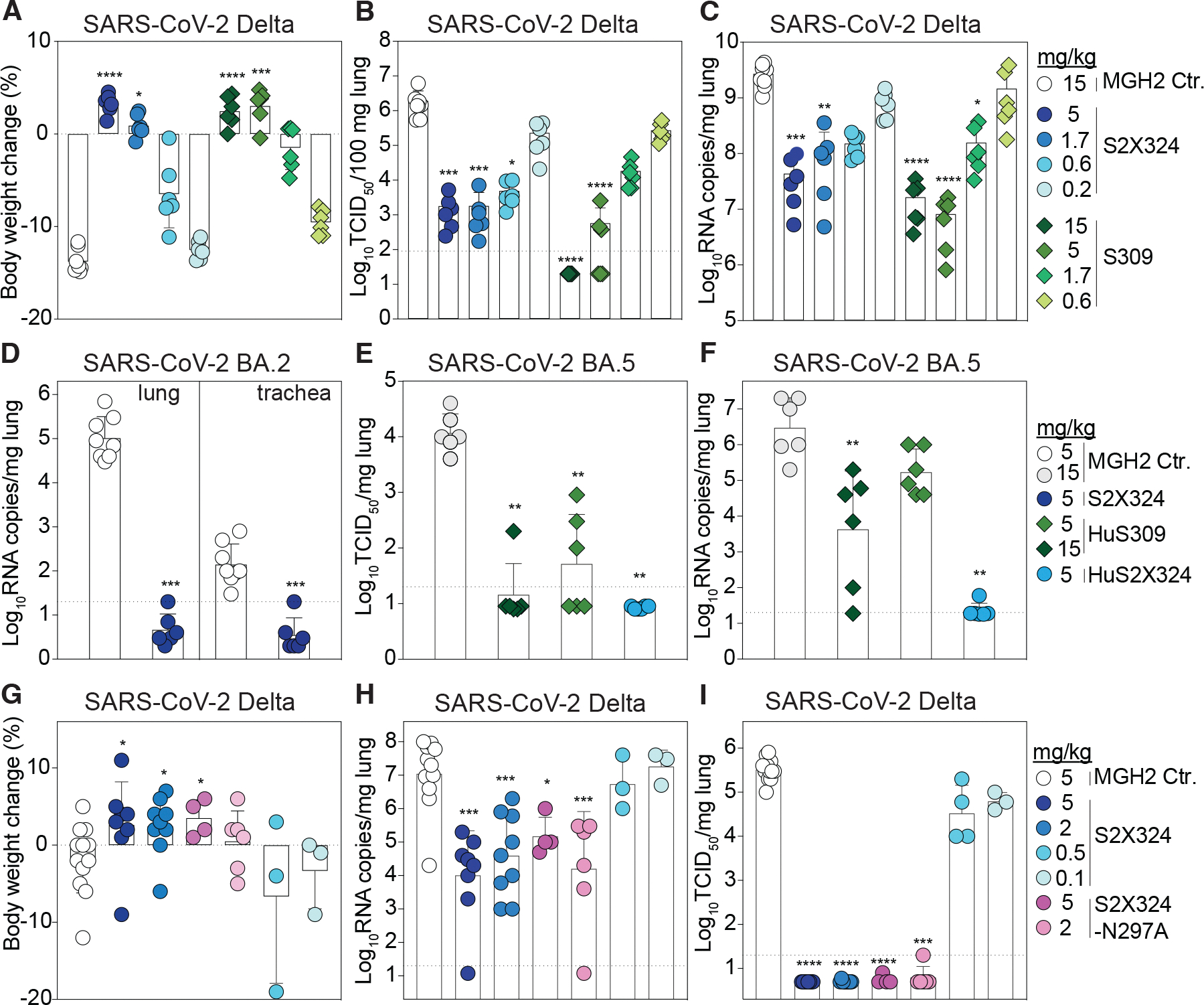
S2X324 protects hamsters against SARS-CoV-2 Delta, BA.2 and BA.5 challenge. **A-C,** Dose-dependent (expressed in mg of mAb/kg of body weight) prophylactic protection of S2X324 (blue circles) and S309 (green diamonds) hamster IgG2a (harboring hamster IgG2a constant regions) administered to animals one day before infection with SARS-CoV-2 Delta and evaluated 4 days post infection based on fraction of body weight change (A), replicating viral titers [50% tissue culture infectious dose (TCID_50_)] (B) and viral RNA load (C). (n=6 animals/dose) *, **, ***, **** p< 0.05, p< 0.01, 0.001, and 0.0001 relative to isotype control (MGH2 mAb against circumsporozoite protein of Plasmodium sporozoites), respectively (Kruskal-Wallis test followed by Dunn’s multiple comparison test). **D**, Quantification of viral RNA loads in the lung and trachea of Syrian hamsters 4 days after intranasal infection with SARS-CoV-2 Omicron BA.2, which was preceded one day prior by prophylactic intraperitoneal administration of S2X324 hamster IgG2a at 5 mg/kg of body weight. *** p<0.001 relative to control, respectively (Mann-Whitney 2-tail T test). **E-F**, Quantification of replicating virus titers (TCID_50_) (E) and viral RNA load (F) in the lung of Syrian hamsters 4 days after intranasal infection with SARS-CoV-2 Omicron BA.5, which was preceded 1 day prior by prophylactic intraperitoneal administration of S309 or S2X324 human IgG1 (HuS309 and HuS2X324) at 5 mg/kg of body weight. **G-I,** Dose-dependent protection in animals 4 days post infection with SARS-CoV-2 Delta by therapeutic intraperitoneal administration of S2X324 hamster IgG2a (blue symbols) or the S2X324 N297A mutant IgG2a (purple symbols) one day later at 5, 2, 0.5 or 0.1 mg/kg of body weight. *, **, ***, **** p< 0.05, p< 0.01, 0.001, and 0.0001 relative to control, respectively (Mann-Whitney 2-tail T test).

We investigated the in vivo prophylactic and therapeutic efficacy of S2X324 using Syrian hamsters challenged with SARS-CoV-2 variants. Prophylactic administration of S2X324 or S309 protected comparably hamsters challenged with SARS-CoV-2 Delta in a dose-dependent manner (Fig. 4A–C), despite a 20-fold difference in in vitro potency against SARS-CoV-2 Delta S VSV (Fig. 2B). These data support the lack of direct correlation between in vitro and in vivo potency as previously reported (51, 52). Moreover, prophylactic administration of S2X324 at 5 mg/kg decreased viral loads below detection levels in the lungs of hamsters challenged with BA.2 or BA.5 (Fig. 4D–F). In this model, S309 retained activity against BA.5, despite a 22.6-fold reduced in vitro potency relative to Wu-G614 (Fig. 2A–B). Therapeutic administration of hamster IgG2a S2X324 (one day after challenge with the SARS-CoV-2 Delta variant) at 2 and 5 mg/kg prevented body weight loss and reduced lung viral RNA loads by 2.5 and 3 orders of magnitude compared to the control group, respectively (Fig. 4G–H). Viral replication in the lungs was fully abrogated at 2 and 5 mg/kg of S2X324 and reduced by approximately one order of magnitude for animals treated with 0.1 and 0.5 mg/kg of S2X324 (Fig. 4I). No statistically significant differences were observed for animals receiving an Fc-silenced version of S2X324 (N297A) versus the groups receiving the same doses of Fc-competent S2X324, indicating limited contributions of Fc-mediated effector functions in these experimental conditions.

## Discussion

Immune imprinting, which is also defined as original antigenic sin, was described based on the observation that infections with influenza virus strains distinct from the strains that caused a prior infection, preferentially boosted antibody responses against epitopes shared with the original strain (53). Although this phenomenon is often considered detrimental, it can also be beneficial, as was the case at the time of the 2009 H1N1 pandemic during which initial antibody responses to infection with this newly emerged and antigenically shifted virus were dominated by antibodies targeting the conserved hemagglutinin stem region (54, 55). Subsequent exposures through vaccination or infection elicited antibody responses to the shifted variant (i.e., to “non-conserved” hemagglutinin epitopes) (54, 56). Moreover, several studies reported hemagglutinin stem-directed antibody-mediated protection against H5N1 and H7N9 zoonotic influenza strains through imprinting during childhood resulting from exposure to seasonal H1N1 and H3N2, respectively (55, 57). Similarly, we show that exposure to antigenically shifted Omicron strains primarily recalls existing memory B cells specific for epitopes shared by multiple SARS-CoV-2 variants rather than by priming naïve B cells recognizing Omicron-specific epitopes (at least up to 180 days post breakthrough infection), as also recently reported (58). Although immune imprinting may be beneficial for stimulating responses to cross-reactive SARS-CoV-2 S epitopes, antibody responses to some Omicron S-specific epitopes were hindered by prior antigenic exposure.

Currently, there is uncertainty regarding the need for vaccines matching dominant circulating SARS-CoV-2 variants (like those used for seasonal influenza) or if the repeated use of Wuhan-Hu-1-based vaccines will suffice. Recent work showed that boosting previously immunized macaques with Beta or Omicron mRNA S vaccines or with Beta RBD nanoparticle vaccines elicited comparably high titers of antibodies broadly neutralizing multiple variants relative to Wuhan-Hu-1-based vaccines (59–61). Furthermore, administration of Wuhan-Hu-1-based vaccine boosters in humans was shown to elicit appreciable titers of neutralizing antibodies and prevent severe disease associated with Omicron infections (11, 19, 62–65). The limited cross-variant neutralization elicited by Omicron primary infection in humans or Omicron-based vaccination of immunologically naïve animals and the data on the specificity of memory B cells presented here indicate that an Omicron-based vaccine might elicit antibody responses directed towards the vaccine-matched and closely related antigens, suggesting that a heterologous prime-boost or a multivalent approach might be preferable (59, 66–73). Omicron infection and Omicron S-based vaccination of previously immune subjects, however, recalls cross-reactive memory B cells (58, 74) which may further mature overtime to enhance their affinity and neutralizing potency against Omicron, but also to possibly broaden their neutralizing activity against past and future variants. Indeed, multiple studies showed that somatic hypermutations yield RBD-specific mAbs with increased affinity for the homotypic antigen and with augmented resilience to immune evasion of emerging heterotypic variants (40, 75–79). Finally, the recently introduced bivalent mRNA vaccine boosters encoding the Wuhan-Hu-1 and either the BA.1 or the BA.4/5 S glycoproteins have yielded encouraging results (80–82).

Understanding antibody responses elicited by and directed towards Omicron sublineages is key to inform public health policies and the design of SARS-CoV-2 and sarbecovirus vaccines (70, 71, 83–85). Our data show that Omicron breakthrough infections did not elicit high titers of pan-sarbecovirus neutralizing antibodies (e.g., directed against SARS-CoV), in agreement with recent data (86). These findings contrast with the observation that pre-existing immunity to SARS-CoV followed by SARS-CoV-2 vaccination was associated with elicitation of pan-sarbecovirus neutralizing antibodies (28). These different outcomes might be explained by the low frequency of memory B cells encoding for neutralizing antibodies targeting antigenic sites shared between pre-Omicron variants (Wuhan-Hu-1-related strains), Omicron and SARS-CoV, due to the genetic and antigenic distances between these three distinct viruses. For instance, Omicron BA.1 and BA.2 harbor variations of the RBD antigenic site II, which is the target of pan-sarbecovirus neutralizing antibodies such as S2X259 (34), DH1047 (87) and ADG2 (88), leading to resistance to neutralization mediated by some of these mAbs (3, 8, 18). This suggests that conservation of RBD antigenic sites across sarbecoviruses may have resulted (at least partially) from limited immune pressure rather than from functional or structural constraints (i.e., some mutations at these conserved sites may remain compatible with viral fitness) (86).

Finally, recent preclinical assessment of intranasally administered influenza and sarbecovirus vaccine candidates demonstrated the induction of lung-resident protective mucosal humoral and cellular immunity at the site of viral entry (89–92). These observations, along with our findings that SARS-CoV-2 breakthrough infections, but not vaccination-only, elicited neutralizing activity in the nasal mucosa motivate the development and evaluation of a next generation of vaccines administered intranasally.

## Supplementary Material

SuppMatt

DataS1

## Data Availability

The cryoEM map and coordinates have been deposited to the Electron Microscopy Databank and Protein Data Bank with accession numbers listed in Table S5. Materials generated in this study will be made available on request, but may require a completed materials transfer agreement signed with Vir Biotechnology Inc. or the University of Washington.

## References

[1] Viana R, Moyo S, Amoako DG, Tegally H, Scheepers C, Althaus CL, Anyaneji UJ, Bester PA, Boni MF, Chand M, Choga WT, Colquhoun R, Davids M, Deforche K, Doolabh D, du Plessis L, Engelbrecht S, Everatt J, Giandhari J, Giovanetti M, Hardie D, Hill V, Hsiao N-Y, Iranzadeh A, Ismail A, Joseph C, Joseph R, Koopile L, Kosakovsky Pond SL, Kraemer MUG, Kuate-Lere L, Laguda-Akingba O, Lesetedi-Mafoko O, Lessells RJ, Lockman S, Lucaci AG, Maharaj A, Mahlangu B, Maponga T, Mahlakwane K, Makatini Z, Marais G, Maruapula D, Masupu K, Matshaba M, Mayaphi S, Mbhele N, Mbulawa MB, Mendes A, Mlisana K, Mnguni A, Mohale T, Moir M, Moruisi K, Mosepele M, Motsatsi G, Motswaledi MS, Mphoyakgosi T, Msomi N, Mwangi PN, Naidoo Y, Ntuli N, Nyaga M, Olubayo L, Pillay S, Radibe B, Ramphal Y, Ramphal U, San JE, Scott L, Shapiro R, Singh L, Smith-Lawrence P, Stevens W, Strydom A, Subramoney K, Tebeila N, Tshiabuila D, Tsui J, van Wyk S, Weaver S, Wibmer CK, Wilkinson E, Wolter N, Zarebski AE, Zuze B, Goedhals D, Preiser W, Treurnicht F, Venter M, Williamson C, Pybus OG, Bhiman J, Glass A, Martin DP, Rambaut A, Gaseitsiwe S, von Gottberg A, de Oliveira T, Rapid epidemic expansion of the SARS-CoV-2 Omicron variant in southern Africa. Nature (2022), doi:10.1038/d41586-021-03832-5. PMC8942855 35042229

[2] Yu J, Collier A-RY, Rowe M, Mardas F, Ventura JD, Wan H, Miller J, Powers O, Chung B, Siamatu M, Hachmann NP, Surve N, Nampanya F, Chandrashekar A, Barouch DH, Comparable neutralization of the SARS-CoV-2 Omicron BA.1 and BA.2 variants. medRxiv (2022), doi:10.1101/2022.02.06.22270533. PMC9006770 35294809

[3] Cameroni E, Bowen JE, Rosen LE, Saliba C, Zepeda SK, Culap K, Pinto D, VanBlargan LA, De Marco A, di Iulio J, Zatta F, Kaiser H, Noack J, Farhat N, Czudnochowski N, Havenar-Daughton C, Sprouse KR, Dillen JR, Powell AE, Chen A, Maher C, Yin L, Sun D, Soriaga L, Bassi J, Silacci-Fregni C, Gustafsson C, Franko NM, Logue J, Iqbal NT, Mazzitelli I, Geffner J, Grifantini R, Chu H, Gori A, Riva A, Giannini O, Ceschi A, Ferrari P, Cippà PE, Franzetti-Pellanda A, Garzoni C, Halfmann PJ, Kawaoka Y, Hebner C, Purcell LA, Piccoli L, Pizzuto MS, Walls AC, Diamond MS, Telenti A, Virgin HW, Lanzavecchia A, Snell G, Veesler D, Corti D, Broadly neutralizing antibodies overcome SARS-CoV-2 Omicron antigenic shift. Nature (2021), doi:10.1038/d41586-021-03825-4. PMC9531318 35016195

[4] Bowen JE, Addetia A, Dang HV, Stewart C, Brown JT, Sharkey WK, Sprouse KR, Walls AC, Mazzitelli IG, Logue JK, Franko NM, Czudnochowski N, Powell AE, Dellota E Jr, Ahmed K, Ansari AS, Cameroni E, Gori A, Bandera A, Posavad CM, Dan JM, Zhang Z, Weiskopf D, Sette A, Crotty S, Iqbal NT, Corti D, Geffner J, Snell G, Grifantini R, Chu HY, Veesler D, Omicron spike function and neutralizing activity elicited by a comprehensive panel of vaccines. Science, eabq0203 (2022). 10.1126/science.abq0203 PMC9348749 35857529

[5] Tan C-W, Lim B-L, Young BE, Yeoh AY-Y, Yung C-F, Yap W-C, Althaus T, Chia W-N, Zhu F, Lye DC, Wang L-F, Comparative neutralisation profile of SARS-CoV-2 omicron subvariants BA.2.75 and BA.5. Lancet Microbe (2022), doi:10.1016/S2666-5247(22)00220-8. PMC9365316 35963276

[6] Desingu PA, Nagarajan K, Dhama K, Emergence of Omicron third lineage BA.3 and its importance. J. Med. Virol. 94, 1808–1810 (2022). 35043399 10.1002/jmv.27601 PMC9015590

[7] Tegally H, Moir M, Everatt J, Giovanetti M, Scheepers C, Wilkinson E, Subramoney K, Moyo S, Amoako DG, Baxter C, Althaus CL, Anyaneji UJ, Kekana D, Viana R, Giandhari J, Lessells RJ, Maponga T, Maruapula D, Choga W, Matshaba M, Mayaphi S, Mbhele N, Mbulawa MB, Msomi N, Naidoo Y, Pillay S, Sanko TJ, San JE, Scott L, Singh L, Magini NA, Smith-Lawrence P, Stevens W, Dor G, Tshiabuila D, Wolter N, Preiser W, Treurnicht FK, Venter M, Davids M, Chiloane G, Mendes A, McIntyre C, O’Toole A, Ruis C, Peacock TP, Roemer C, Williamson C, Pybus OG, Bhiman J, Glass A, Martin DP, Rambaut A, Gaseitsiwe S, von Gottberg A, de Oliveira T, NGS-SA consortium, Continued emergence and evolution of Omicron in South Africa: New BA.4 and BA.5 lineages. bioRxiv (2022), doi:10.1101/2022.05.01.22274406.

[8] Liu L, Iketani S, Guo Y, Chan JF-W, Wang M, Liu L, Luo Y, Chu H, Huang Y, Nair MS, Yu J, Chik KK-H, Yuen TT-T, Yoon C, To KK-W, Chen H, Yin MT, Sobieszczyk ME, Huang Y, Wang HH, Sheng Z, Yuen K-Y, Ho DD, Striking antibody evasion manifested by the Omicron variant of SARS-CoV-2. Nature (2021), doi:10.1038/d41586-021-03826-3. 35016198

[9] Planas D, Saunders N, Maes P, Guivel-Benhassine F, Planchais C, Buchrieser J, Bolland W-H, Porrot F, Staropoli I, Lemoine F, Péré H, Veyer D, Puech J, Rodary J, Baela G, Dellicour S, Raymenants J, Gorissen S, Geenen C, Vanmechelen B, Wawina-Bokalanga T, Martí-Carrerasi J, Cuypers L, Sève A, Hocqueloux L, Prazuck T, Rey F, Simon-Lorrière E, Bruel T, Mouquet H, André E, Schwartz O, Considerable escape of SARS-CoV-2 Omicron to antibody neutralization. Nature (2021), doi:10.1038/d41586-021-03827-2. 35016199

[10] Hoffmann M, Krüger N, Schulz S, Cossmann A, Rocha C, Kempf A, Nehlmeier I, Graichen L, Moldenhauer A-S, Winkler MS, Lier M, Dopfer-Jablonka A, Jäck H-M, Behrens GMN, Pöhlmann S, The Omicron variant is highly resistant against antibody-mediated neutralization – implications for control of the COVID-19 pandemic. bioRxiv (2021), doi:10.1101/2021.12.12.472286. PMC8702401 35026151

[11] Garcia-Beltran WF, St Denis KJ, Hoelzemer A, Lam EC, Nitido AD, Sheehan ML, Berrios C, Ofoman O, Chang CC, Hauser BM, Feldman J, Roederer AL, Gregory DJ, Poznansky MC, Schmidt AG, Iafrate AJ, Naranbhai V, Balazs AB, mRNA-based COVID-19 vaccine boosters induce neutralizing immunity against SARS-CoV-2 Omicron variant. Cell. 185, 457–466.e4 (2022). 34995482 10.1016/j.cell.2021.12.033 PMC8733787

[12] Gruell H, Vanshylla K, Korenkov M, Tober-Lau P, Zehner M, Münn F, Janicki H, Augustin M, Schommers P, Sander LE, Kurth F, Kreer C, Klein F, Delineating antibody escape from Omicron variants. bioRxiv (2022), doi:10.1101/2022.04.06.487257. PMC9260412 35921836

[13] Piccoli L, Park YJ, Tortorici MA, Czudnochowski N, Walls AC, Beltramello M, Silacci-Fregni C, Pinto D, Rosen LE, Bowen JE, Acton OJ, Jaconi S, Guarino B, Minola A, Zatta F, Sprugasci N, Bassi J, Peter A, De Marco A, Nix JC, Mele F, Jovic S, Rodriguez BF, Gupta SV, Jin F, Piumatti G, Lo Presti G, Pellanda AF, Biggiogero M, Tarkowski M, Pizzuto MS, Cameroni E, Havenar-Daughton C, Smithey M, Hong D, Lepori V, Albanese E, Ceschi A, Bernasconi E, Elzi L, Ferrari P, Garzoni C, Riva A, Snell G, Sallusto F, Fink K, Virgin HW, Lanzavecchia A, Corti D, Veesler D, Mapping Neutralizing and Immunodominant Sites on the SARS-CoV-2 Spike Receptor-Binding Domain by Structure-Guided High-Resolution Serology. Cell. 183, 1024–1042.e21 (2020). 32991844 10.1016/j.cell.2020.09.037 PMC7494283

[14] Bowen JE, Walls AC, Joshi A, Sprouse KR, Stewart C, Alejandra Tortorici M, Franko NM, Logue JK, Mazzitelli IG, Tiles SW, Ahmed K, Shariq A, Snell G, Iqbal NT, Geffner J, Bandera A, Gori A, Grifantini R, Chu HY, Van Voorhis WC, Corti D, Veesler D, SARS-CoV-2 spike conformation determines plasma neutralizing activity. bioRxiv (2021), p. 2021.12.19.473391, doi:10.1101/2021.12.19.473391.

[15] Stamatatos L, Czartoski J, Wan Y-H, Homad LJ, Rubin V, Glantz H, Neradilek M, Seydoux E, Jennewein MF, MacCamy AJ, Feng J, Mize G, De Rosa SC, Finzi A, Lemos MP, Cohen KW, Moodie Z, Juliana McElrath M, McGuire AT, mRNA vaccination boosts cross-variant neutralizing antibodies elicited by SARS-CoV-2 infection. Science (2021), p. eabg9175. 33766944 10.1126/science.abg9175 PMC8139425

[16] Greaney AJ, Loes AN, Gentles LE, Crawford KHD, Starr TN, Malone KD, Chu HY, Bloom JD, Antibodies elicited by mRNA-1273 vaccination bind more broadly to the receptor binding domain than do those from SARS-CoV-2 infection. Sci. Transl. Med. 13 (2021), doi:10.1126/scitranslmed.abi9915. PMC8369496 34103407

[17] McCallum M, De Marco A, Lempp FA, Tortorici MA, Pinto D, Walls AC, Beltramello M, Chen A, Liu Z, Zatta F, Zepeda S, di Iulio J, Bowen JE, Montiel-Ruiz M, Zhou J, Rosen LE, Bianchi S, Guarino B, Fregni CS, Abdelnabi R, Caroline Foo S-Y, Rothlauf PW, Bloyet L-M, Benigni F, Cameroni E, Neyts J, Riva A, Snell G, Telenti A, Whelan SPJ, Virgin HW, Corti D, Pizzuto MS, Veesler D, N-terminal domain antigenic mapping reveals a site of vulnerability for SARS-CoV-2. Cell (2021), doi:10.1016/j.cell.2021.03.028. PMC7962585 33761326

[18] McCallum M, Czudnochowski N, Rosen LE, Zepeda SK, Bowen JE, Walls AC, Hauser K, Joshi A, Stewart C, Dillen JR, Powell AE, Croll TI, Nix J, Virgin HW, Corti D, Snell G, Veesler D, Structural basis of SARS-CoV-2 Omicron immune evasion and receptor engagement. Science, eabn8652 (2022). 35076256 10.1126/science.abn8652 PMC9427005

[19] Walls AC, Sprouse KR, Bowen JE, Joshi A, Franko N, Navarro MJ, Stewart C, Cameroni E, McCallum M, Goecker EA, Degli-Angeli EJ, Logue J, Greninger A, Corti D, Chu HY, Veesler D, SARS-CoV-2 breakthrough infections elicit potent, broad, and durable neutralizing antibody responses. Cell (2022), doi:10.1016/j.cell.2022.01.011. PMC8769922 35123650

[20] Collier A-RY, Brown CM, McMahan KA, Yu J, Liu J, Jacob-Dolan C, Chandrashekar A, Tierney D, Ansel JL, Rowe M, Sellers D, Ahmad K, Aguayo R, Anioke T, Gardner S, Siamatu M, Bermudez Rivera L, Hacker MR, Madoff LC, Barouch DH, Characterization of immune responses in fully vaccinated individuals following breakthrough infection with the SARS-CoV-2 delta variant. Sci. Transl. Med, eabn6150 (2022). 35258323 10.1126/scitranslmed.abn6150 PMC8995036

[21] Bates TA, McBride SK, Winders B, Schoen D, Trautmann L, Curlin ME, Tafesse FG, Antibody Response and Variant Cross-Neutralization After SARS-CoV-2 Breakthrough Infection. JAMA. 327, 179–181 (2022). 34914825 10.1001/jama.2021.22898 PMC8678894

[22] Mlcochova P, Kemp S, Dhar MS, Papa G, Meng B, Mishra S, Whittaker C, Mellan T, Ferreira I, Datir R, Collier DA, Singh S, Pandey R, Marwal R, Datta M, Sengupta S, Ponnusamy K, Radhakrishnan VS, Abdullahi A, Goonawardne N, Brown J, Charles O, Chattopadhyay P, Devi P, Caputo D, Peacock T, Wattal C, Goel N, Vaishya R, Agarwal M, o. H. Lee, Barcla WS, Bhatt S, Flaxman S, James L, Rakshit P, Agrawal A, The Indian SARS-CoV-2 Genomics Consortium (INSACOG), CITIID-NIHR BioResource COVID-19 Collaboration, Mavousian A, Gupta RK, SARS-CoV-2 B.1.617.2 Delta variant emergence and vaccine breakthrough, doi:10.21203/rs.3.rs-637724/v1.

[23] McCallum M, Walls AC, Sprouse KR, Bowen JE, Rosen LE, Dang HV, De Marco A, Franko N, Tilles SW, Logue J, Miranda MC, Ahlrichs M, Carter L, Snell G, Pizzuto MS, Chu HY, Van Voorhis WC, Corti D, Veesler D, Molecular basis of immune evasion by the Delta and Kappa SARS-CoV-2 variants. Science, eabl8506 (2021). 10.1126/science.abl8506 PMC12240541 34751595

[24] Suzuki R, Yamasoba D, Kimura I, Wang L, Kishimoto M, Ito J, Morioka Y, Nao N, Nasser H, Uriu K, Kosugi Y, Tsuda M, Orba Y, Sasaki M, Shimizu R, Kawabata R, Yoshimatsu K, Asakura H, Nagashima M, Sadamasu K, Yoshimura K, Genotype to Phenotype Japan (G2P-Japan) Consortium, Sawa H, Ikeda T, Irie T, Matsuno K, Tanaka S, Fukuhara T, Sato K, Attenuated fusogenicity and pathogenicity of SARS-CoV-2 Omicron variant. Nature (2022), doi:10.1038/s41586-022-04462-1. PMC8942852 35104835

[25] Mlcochova P, Kemp S, Dhar MS, Papa G, Meng B, Ferreira IATM, Datir R, Collier DA, Albecka A, Singh S, Pandey R, Brown J, Zhou J, Goonawardane N, Mishra S, Whittaker C, Mellan T, Marwal R, Datta M, Sengupta S, Ponnusamy K, Radhakrishnan VS, Abdullahi A, Charles O, Chattopadhyay P, Devi P, Caputo D, Peacock T, Wattal DC, Goel N, Satwik A, Vaishya R, Agarwal M, Indian SARS-CoV-2 Genomics Consortium (INSACOG), Genotype to Phenotype Japan (G2P-Japan) Consortium, CITIID-NIHR BioResource COVID-19 Collaboration, Mavousian A, Lee JH, Bassi J, Silacci-Fegni C, Saliba C, Pinto D, Irie T, Yoshida I, Hamilton WL, Sato K, Bhatt S, Flaxman S, James LC, Corti D, Piccoli L, Barclay WS, Rakshit P, Agrawal A, Gupta RK, SARS-CoV-2 B.1.617.2 Delta variant replication and immune evasion. Nature (2021), doi:10.1038/s41586-021-03944-y. PMC8566220 34488225

[26] Crotty S, Hybrid immunity. Science. 372, 1392–1393 (2021).

[27] Starr TN, Czudnochowski N, Liu Z, Zatta F, Park Y-J, Addetia A, Pinto D, Beltramello M, Hernandez P, Greaney AJ, Marzi R, Glass WG, Zhang I, Dingens AS, Bowen JE, Tortorici MA, Walls AC, Wojcechowskyj JA, De Marco A, Rosen LE, Zhou J, Montiel-Ruiz M, Kaiser H, Dillen JR, Tucker H, Bassi J, Silacci-Fregni C, Housley MP, di Iulio J, Lombardo G, Agostini M, Sprugasci N, Culap K, Jaconi S, Meury M, Dellota E Jr, Abdelnabi R, Foo S-YC, Cameroni E, Stumpf S, Croll TI, Nix JC, Havenar-Daughton C, Piccoli L, Benigni F, Neyts J, Telenti A, Lempp FA, Pizzuto MS, Chodera JD, Hebner CM, Virgin HW, Whelan SPJ, Veesler D, Corti D, Bloom JD, Snell G, SARS-CoV-2 RBD antibodies that maximize breadth and resistance to escape. Nature. 597, 97–102 (2021). 34261126 10.1038/s41586-021-03807-6 PMC9282883

[28] Tan C-W, Chia W-N, Young BE, Zhu F, Lim B-L, Sia W-R, Thein T-L, Chen MI-C, Leo Y-S, Lye DC, Wang L-F, Pan-Sarbecovirus Neutralizing Antibodies in BNT162b2-Immunized SARS-CoV-1 Survivors. N. Engl. J. Med. 385, 1401–1406 (2021). 34407341 10.1056/NEJMoa2108453 PMC8422514

[29] Pinna D, Corti D, Jarrossay D, Sallusto F, Lanzavecchia A, Clonal dissection of the human memory B-cell repertoire following infection and vaccination. Eur. J. Immunol. 39, 1260–1270 (2009). 19404981 10.1002/eji.200839129 PMC3864550

[30] Khan K, Karim F, Ganga Y, Bernstein M, Jule Z, Reedoy K, Cele S, Lustig G, Amoako D, Wolter N, Samsunder N, Sivro A, San JE, Giandhari J, Tegally H, Pillay S, Naidoo Y, Mazibuko M, Miya Y, Ngcobo N, Manickchund N, Magula N, Karim QA, von Gottberg A, Abdool Karim SS, Hanekom W, Gosnell BI, Lessells RJ, de Oliveira T, Moosa M-YS, Sigal A, COMMIT-KZN Team, Omicron sub-lineages BA.4/BA.5 escape BA.1 infection elicited neutralizing immunity (2022), doi:10.1101/2022.04.29.22274477. PMC9364294 35948557

[31] Muik A, Lui BG, Bacher M, Wallisch A-K, Toker A, Finlayson A, Krüger K, Ozhelvaci O, Grikscheit K, Hoehl S, Ciesek S, Türeci Ö, Sahin U, Omicron BA.2 breakthrough infection enhances cross-neutralization of BA.2.12.1 and BA.4/BA.5. bioRxiv (2022), doi:10.1101/2022.08.02.502461. PMC9529054 36125366

[32] Tan CW, Chia WN, Qin X, Liu P, Chen MIC, Tiu C, Hu Z, Chen VC-W, Young BE, Sia WR, Tan Y-J, Foo R, Yi Y, Lye DC, Anderson DE, Wang L-F, A SARS-CoV-2 surrogate virus neutralization test based on antibody-mediated blockage of ACE2–spike protein–protein interaction. Nat. Biotechnol. (2020), doi:10.1038/s41587-020-0631-z. 32704169

[33] Tortorici MA, Beltramello M, Lempp FA, Pinto D, Dang HV, Rosen LE, McCallum M, Bowen J, Minola A, Jaconi S, Zatta F, De Marco A, Guarino B, Bianchi S, Lauron EJ, Tucker H, Zhou J, Peter A, Havenar-Daughton C, Wojcechowskyj JA, Case JB, Chen RE, Kaiser H, Montiel-Ruiz M, Meury M, Czudnochowski N, Spreafico R, Dillen J, Ng C, Sprugasci N, Culap K, Benigni F, Abdelnabi R, Foo SC, Schmid MA, Cameroni E, Riva A, Gabrieli A, Galli M, Pizzuto MS, Neyts J, Diamond MS, Virgin HW, Snell G, Corti D, Fink K, Veesler D, Ultrapotent human antibodies protect against SARS-CoV-2 challenge via multiple mechanisms. Science. 370, 950–957 (2020). 32972994 10.1126/science.abe3354 PMC7857395

[34] Tortorici MA, Czudnochowski N, Starr TN, Marzi R, Walls AC, Zatta F, Bowen JE, Jaconi S, Di Iulio J, Wang Z, De Marco A, Zepeda SK, Pinto D, Liu Z, Beltramello M, Bartha I, Housley MP, Lempp FA, Rosen LE, Dellota E Jr, Kaiser H, Montiel-Ruiz M, Zhou J, Addetia A, Guarino B, Culap K, Sprugasci N, Saliba C, Vetti E, Giacchetto-Sasselli I, Fregni CS, Abdelnabi R, Foo S-YC, Havenar-Daughton C, Schmid MA, Benigni F, Cameroni E, Neyts J, Telenti A, Virgin HW, Whelan SPJ, Snell G, Bloom JD, Corti D, Veesler D, Pizzuto MS, Broad sarbecovirus neutralization by a human monoclonal antibody. Nature. 597, 103–108 (2021). 34280951 10.1038/s41586-021-03817-4 PMC9341430

[35] Azzi L, Dalla Gasperina D, Veronesi G, Shallak M, Ietto G, Iovino D, Baj A, Gianfagna F, Maurino V, Focosi D, Maggi F, Ferrario MM, Dentali F, Carcano G, Tagliabue A, Maffioli LS, Accolla RS, Forlani G, Mucosal immune response in BNT162b2 COVID-19 vaccine recipients. EBioMedicine. 75, 103788 (2022). 34954658 10.1016/j.ebiom.2021.103788 PMC8718969

[36] Tang J, Zeng C, Cox TM, Li C, Son YM, Cheon IS, Wu Y, Behl S, Taylor JJ, Chakraborty R, Johnson AJ, Schiavo DN, Utz JP, Reisenauer JS, Midthun DE, Mullon JJ, Edell ES, Alameh MG, Borish L, Teague WG, Kaplan MH, Weissman D, Kern R, Hu H, Vassallo R, Liu S-L, Sun J, Respiratory mucosal immunity against SARS-CoV-2 following mRNA vaccination. Sci. Immunol, eadd4853 (2022). 35857583 10.1126/sciimmunol.add4853 PMC9348751

[37] Planas D, Staropoli I, Porot F, Guivel-Benhassine F, Handala L, Prot M, Bolland W-H, Puech J, Péré H, Veyer D, Sève A, Etienne-Simon-Lorière, Bruel T, Prazuck T, Stefic K, Hocqueloux L, Schwartz O, Duration of BA.5 neutralization in sera and nasal swabs from SARS-CoV-2 vaccinated individuals, with or without Omicron breakthrough infection. bioRxiv (2022), doi:10.1101/2022.07.22.22277885. PMC9533668 36228619

[38] Lim JME, Tan AT, Le Bert N, Hang SK, Low JGH, Bertoletti A, SARS-CoV-2 breakthrough infection in vaccinees induces virus-specific nasal-resident CD8+ and CD4+ T cells of broad specificity. J. Exp. Med. 219 (2022), doi:10.1084/jem.20220780. PMC9386509 35972472

[39] Dong J, Zost SJ, Greaney AJ, Starr TN, Dingens AS, Chen EC, Chen RE, Case JB, Sutton RE, Gilchuk P, Rodriguez J, Armstrong E, Gainza C, Nargi RS, Binshtein E, Xie X, Zhang X, Shi P-Y, Logue J, Weston S, McGrath ME, Frieman MB, Brady T, Tuffy KM, Bright H, Loo Y-M, McTamney PM, Esser MT, Carnahan RH, Diamond MS, Bloom JD, Crowe JE Jr, Genetic and structural basis for SARS-CoV-2 variant neutralization by a two-antibody cocktail. Nat Microbiol. 6, 1233–1244 (2021). 34548634 10.1038/s41564-021-00972-2 PMC8543371

[40] Park Y-J, De Marco A, Starr TN, Liu Z, Pinto D, Walls AC, Zatta F, Zepeda SK, Bowen JE, Sprouse KR, Joshi A, Giurdanella M, Guarino B, Noack J, Abdelnabi R, Foo S-YC, Rosen LE, Lempp FA, Benigni F, Snell G, Neyts J, Whelan SPJ, Virgin HW, Bloom JD, Corti D, Pizzuto MS, Veesler D, Antibody-mediated broad sarbecovirus neutralization through ACE2 molecular mimicry. Science. 375, 449–454 (2022). 34990214 10.1126/science.abm8143 PMC9400459

[41] Pinto D, Park Y-J, Beltramello M, Walls AC, Alejandra Tortorici M, Bianchi S, Jaconi S, Culap K, Zatta F, De Marco A, Peter A, Guarino B, Spreafico R, Cameroni E, Case JB, Chen RE, Havenar-Daughton C, Snell G, Telenti A, Virgin HW, Lanzavecchia A, Diamond MS, Fink K, Veesler D, Corti D, Cross-neutralization of SARS-CoV-2 by a human monoclonal SARS-CoV antibody. Nature. 583 (2020), pp. 290–295. 32422645 10.1038/s41586-020-2349-y

[42] Cameroni E, Saliba C, Bowen JE, Rosen LE, Culap K, Pinto D, De Marco A, Zepeda SK, di Iulio J, Zatta F, Kaiser H, Noack J, Farhat N, Czudnochowski N, Havenar-Daughton C, Sprouse KR, Dillen JR, Powell AE, Chen A, Maher C, Yin L, Sun D, Soriaga L, Gustafsson C, Franko NM, Logue J, Iqbal NT, Mazzitelli I, Geffner J, Grifantini R, Chu H, Gori A, Riva A, Giannini O, Ceschi A, Ferrari P, Franzetti-Pellanda A, Garzoni C, Hebner C, Purcell LA, Piccoli L, Pizzuto MS, Walls AC, Telenti A, Virgin HW, Lanzavecchia A, Veesler D, Snell G, Corti D, Broadly neutralizing antibodies overcome SARS-CoV-2 Omicron antigenic shift. bioRxiv (2021), doi:10.1101/2021.12.12.472269. PMC9531318 35016195

[43] Cathcart AL, Havenar-Daughton C, Lempp FA, Ma D, Schmid M, Agostini ML, Guarino B, Di iulio J, Rosen L, Tucker H, Dillen J, Subramanian S, Sloan B, Bianchi S, Wojcechowskyj J, Zhou J, Kaiser H, Chase A, Montiel-Ruiz M, Czudnochowski N, Cameroni E, Ledoux S, Colas C, Soriaga L, Telenti A, Hwang S, Snell G, Virgin HW, Corti D, Hebner CM, The dual function monoclonal antibodies VIR-7831 and VIR-7832 demonstrate potent in vitro and in vivo activity against SARS-CoV-2. bioRxiv (2021), doi:10.1101/2021.03.09.434607.

[44] Case JB, Mackin S, Errico JM, Chong Z, Madden EA, Whitener B, Guarino B, Schmid MA, Rosenthal K, Ren K, Dang HV, Snell G, Jung A, Droit L, Handley SA, Halfmann PJ, Kawaoka Y, Crowe JE Jr, Fremont DH, Virgin HW, Loo Y-M, Esser MT, Purcell LA, Corti D, Diamond MS, Resilience of S309 and AZD7442 monoclonal antibody treatments against infection by SARS-CoV-2 Omicron lineage strains. Nat. Commun. 13, 3824 (2022). 35780162 10.1038/s41467-022-31615-7 PMC9250508

[45] Stalls V, Lindenberger J, Gobeil SM-C, Henderson R, Parks R, Barr M, Deyton M, Martin M, Janowska K, Huang X, May A, Speakman M, Beaudoin E, Kraft B, Lu X, Edwards RJ, Eaton A, Montefiori DC, Williams WB, Saunders KO, Wiehe K, Haynes BF, Acharya P, Cryo-EM structures of SARS-CoV-2 Omicron BA.2 spike. Cell Rep. 39, 111009 (2022). 35732171 10.1016/j.celrep.2022.111009 PMC9174147

[46] Walls AC, Park YJ, Tortorici MA, Wall A, McGuire AT, Veesler D, Structure, Function, and Antigenicity of the SARS-CoV-2 Spike Glycoprotein. Cell. 181, 281–292.e6 (2020). 32155444 10.1016/j.cell.2020.02.058 PMC7102599

[47] Walls AC, Xiong X, Park YJ, Tortorici MA, Snijder J, Quispe J, Cameroni E, Gopal R, Dai M, Lanzavecchia A, Zambon M, Rey FA, Corti D, Veesler D, Unexpected Receptor Functional Mimicry Elucidates Activation of Coronavirus Fusion. Cell. 176, 1026–1039.e15 (2019). 30712865 10.1016/j.cell.2018.12.028 PMC6751136

[48] Westendorf K, Žentelis S, Wang L, Foster D, Vaillancourt P, Wiggin M, Lovett E, van der Lee R, Hendle J, Pustilnik A, Sauder JM, Kraft L, Hwang Y, Siegel RW, Chen J, Heinz BA, Higgs RE, Kallewaard NL, Jepson K, Goya R, Smith MA, Collins DW, Pellacani D, Xiang P, de Puyraimond V, Ricicova M, Devorkin L, Pritchard C, O’Neill A, Dalal K, Panwar P, Dhupar H, Garces FA, Cohen CA, Dye JM, Huie KE, Badger CV, Kobasa D, Audet J, Freitas JJ, Hassanali S, Hughes I, Munoz L, Palma HC, Ramamurthy B, Cross RW, Geisbert TW, Menacherry V, Lokugamage K, Borisevich V, Lanz I, Anderson L, Sipahimalani P, Corbett KS, Yang ES, Zhang Y, Shi W, Zhou T, Choe M, Misasi J, Kwong PD, Sullivan NJ, Graham BS, Fernandez TL, Hansen CL, Falconer E, Mascola JR, Jones BE, Barnhart BC, LY-CoV1404 (bebtelovimab) potently neutralizes SARS-CoV-2 variants. bioRxivorg (2022), doi:10.1101/2021.04.30.442182. PMC9035363 35568025

[49] Westendorf K, Žentelis S, Wang L, Foster D, Vaillancourt P, Wiggin M, Lovett E, van der Lee R, Hendle J, Pustilnik A, Sauder JM, Kraft L, Hwang Y, Siegel RW, Chen J, Heinz BA, Higgs RE, Kallewaard NL, Jepson K, Goya R, Smith MA, Collins DW, Pellacani D, Xiang P, de Puyraimond V, Ricicova M, Devorkin L, Pritchard C, O’Neill A, Dalal K, Panwar P, Dhupar H, Garces FA, Cohen CA, Dye JM, Huie KE, Badger CV, Kobasa D, Audet J, Freitas JJ, Hassanali S, Hughes I, Munoz L, Palma HC, Ramamurthy B, Cross RW, Geisbert TW, Menachery V, Lokugamage K, Borisevich V, Lanz I, Anderson L, Sipahimalani P, Corbett KS, Yang ES, Zhang Y, Shi W, Zhou T, Choe M, Misasi J, Kwong PD, Sullivan NJ, Graham BS, Fernandez TL, Hansen CL, Falconer E, Mascola JR, Jones BE, Barnhart BC, LY-CoV1404 (bebtelovimab) potently neutralizes SARS-CoV-2 variants. Cell Rep. 39, 110812 (2022). 35568025 10.1016/j.celrep.2022.110812 PMC9035363

[50] Case JB, Rothlauf PW, Chen RE, Liu Z, Zhao H, Kim AS, Bloyet LM, Zeng Q, Tahan S, Droit L, Ilagan MXG, Tartell MA, Amarasinghe G, Henderson JP, Miersch S, Ustav M, Sidhu S, Virgin HW, Wang D, Ding S, Corti D, Theel ES, Fremont DH, Diamond MS, Whelan SPJ, Neutralizing Antibody and Soluble ACE2 Inhibition of a Replication-Competent VSV-SARS-CoV-2 and a Clinical Isolate of SARS-CoV-2. Cell Host Microbe. 28, 475–485.e5 (2020). 32735849 10.1016/j.chom.2020.06.021 PMC7332453

[51] Schäfer A, Muecksch F, Lorenzi JCC, Leist SR, Cipolla M, Bournazos S, Schmidt F, Maison RM, Gazumyan A, Martinez DR, Baric RS, Robbiani DF, Hatziioannou T, Ravetch JV, Bieniasz PD, Bowen RA, Nussenzweig MC, Sheahan TP, Antibody potency, effector function, and combinations in protection and therapy for SARS-CoV-2 infection in vivo. J. Exp. Med. 218 (2021), doi:10.1084/jem.20201993. PMC7673958 33211088

[52] Case JB, Mackin S, Errico J, Chong Z, Madden EA, Guarino B, Schmid MA, Rosenthal K, Ren K, Jung A, Droit L, Handley SA, Halfmann PJ, Kawaoka Y, Crowe JE Jr, Fremont DH, Virgin HW, Loo Y-M, Esser MT, Purcell LA, Corti D, Diamond MS, Resilience of S309 and AZD7442 monoclonal antibody treatments against infection by SARS-CoV-2 Omicron lineage strains. bioRxiv (2022), doi:10.1101/2022.03.17.484787. PMC9250508 35780162

[53] Francis T, On the doctrine of original antigenic sin. Proc Am Philos Soc. 104, 572–578 (1960).

[54] Corti D, Voss J, Gamblin SJ, Codoni G, Macagno A, Jarrossay D, Vachieri SG, Pinna D, Minola A, Vanzetta F, Silacci C, Fernandez-Rodriguez BM, Agatic G, Bianchi S, Giacchetto-Sasselli I, Calder L, Sallusto F, Collins P, Haire LF, Temperton N, Langedijk JP, Skehel JJ, Lanzavecchia A, A neutralizing antibody selected from plasma cells that binds to group 1 and group 2 influenza A hemagglutinins. Science. 333, 850–856 (2011). 21798894 10.1126/science.1205669

[55] Wrammert J, Koutsonanos D, Li G-M, Edupuganti S, Sui J, Morrissey M, McCausland M, Skountzou I, Hornig M, Lipkin WI, Mehta A, Razavi B, Del Rio C, Zheng N-Y, Lee J-H, Huang M, Ali Z, Kaur K, Andrews S, Amara RR, Wang Y, Das SR, O’Donnell CD, Yewdell JW, Subbarao K, Marasco WA, Mulligan MJ, Compans R, Ahmed R, Wilson PC, Broadly cross-reactive antibodies dominate the human B cell response against 2009 pandemic H1N1 influenza virus infection. J. Exp. Med. 208, 181–193 (2011). 21220454 10.1084/jem.20101352 PMC3023136

[56] Cheung CS-F, Fruehwirth A, Paparoditis PCG, Shen C-H, Foglierini M, Joyce MG, Leung K, Piccoli L, Rawi R, Silacci-Fregni C, Tsybovsky Y, Verardi R, Wang L, Wang S, Yang ES, Zhang B, Zhang Y, Chuang G-Y, Corti D, Mascola JR, Shapiro L, Kwong PD, Lanzavecchia A, Zhou T, Identification and structure of a multidonor class of head-directed influenza-neutralizing antibodies reveal the mechanism for its recurrent elicitation. Cell Rep. 32, 108088 (2020). 32877670 10.1016/j.celrep.2020.108088

[57] Gostic KM, Ambrose M, Worobey M, Lloyd-Smith JO, Potent protection against H5N1 and H7N9 influenza via childhood hemagglutinin imprinting. Science. 354, 722–726 (2016). 27846599 10.1126/science.aag1322 PMC5134739

[58] Quandt J, Muik A, Salisch N, Lui BG, Lutz S, Krüger K, Wallisch A-K, Adams-Quack P, Bacher M, Finlayson A, Ozhelvaci O, Vogler I, Grikscheit K, Hoehl S, Goetsch U, Ciesek S, Türeci Ö, Sahin U, Omicron BA.1 breakthrough infection drives cross-variant neutralization and memory B cell formation against conserved epitopes. Sci. Immunol. 7, eabq2427 (2022). 35653438 10.1126/sciimmunol.abq2427 PMC9162083

[59] Gagne M, Moliva JI, Foulds KE, Andrew SF, Flynn BJ, Werner AP, Wagner DA, Teng I-T, Lin BC, Moore C, Jean-Baptiste N, Carroll R, Foster SL, Patel M, Ellis M, Edara V-V, Maldonado NV, Minai M, McCormick L, Honeycutt CC, Nagata BM, Bock KW, Dulan CNM, Cordon J, Flebbe DR, Todd J-PM, McCarthy E, Pessaint L, Van Ry A, Narvaez B, Valentin D, Cook A, Dodson A, Steingrebe K, Nurmukhambetova ST, Godbole S, Henry AR, Laboune F, Roberts-Torres J, Lorang CG, Amin S, Trost J, Naisan M, Basappa M, Willis J, Wang L, Shi W, Doria-Rose NA, Zhang Y, Yang ES, Leung K, O’Dell S, Schmidt SD, Olia AS, Liu C, Harris DR, Chuang G-Y, Stewart-Jones G, Renzi I, Lai Y-T, Malinowski A, Wu K, Mascola JR, Carfi A, Kwong PD, Edwards DK, Lewis MG, Andersen H, Corbett KS, Nason MC, McDermott AB, Suthar MS, Moore IN, Roederer M, Sullivan NJ, Douek DC, Seder RA, mRNA-1273 or mRNA-Omicron boost in vaccinated macaques elicits similar B cell expansion, neutralizing antibodies and protection against Omicron. Cell (2022), doi:10.1016/j.cell.2022.03.038. PMC8947944 35447072

[60] Corbett KS, Gagne M, Wagner DA, O’ Connell S, Narpala SR, Flebbe DR, Andrew SF, Davis RL, Flynn B, Johnston TS, Stringham CD, Lai L, Valentin D, Van Ry A, Flinchbaugh Z, Werner AP, Moliva JI, Sriparna M, O’Dell S, Schmidt SD, Tucker C, Choi A, Koch M, Bock KW, Minai M, Nagata BM, Alvarado GS, Henry AR, Laboune F, Schramm CA, Zhang Y, Yang ES, Wang L, Choe M, Boyoglu-Barnum S, Wei S, Lamb E, Nurmukhambetova ST, Provost SJ, Donaldson MM, Marquez J, Todd J-PM, Cook A, Dodson A, Pekosz A, Boritz E, Ploquin A, Doria-Rose N, Pessaint L, Andersen H, Foulds KE, Misasi J, Wu K, Carfi A, Nason MC, Mascola J, Moore IN, Edwards DK, Lewis MG, Suthar MS, Roederer M, McDermott A, Douek DC, Sullivan NJ, Graham BS, Seder RA, Protection against SARS-CoV-2 Beta variant in mRNA-1273 vaccine-boosted nonhuman primates. Science. 374, 1343–1353 (2021). 34672695 10.1126/science.abl8912

[61] Arunachalam PS, Feng Y, Ashraf U, Hu M, Walls AC, Edara VV, Zarnitsyna VI, Aye PP, Golden N, Miranda MC, Green KWM, Threeton BM, Maness NJ, Beddingfield BJ, Bohm RP, Scheuermann SE, Goff K, Dufour J, Russell-Lodrigue K, Kepl E, Fiala B, Wrenn S, Ravichandran R, Ellis D, Carter L, Rogers K, Shirreff LM, Ferrell DE, Deb Adhikary NR, Fontenot J, Hammond HL, Frieman M, Grifoni A, Sette A, O’Hagan DT, Van Der Most R, Rappuoli R, Villinger F, Kleanthous H, Rappaport J, Suthar MS, Veesler D, Wang TT, King NP, Pulendran B, Durable protection against the SARS-CoV-2 Omicron variant is induced by an adjuvanted subunit vaccine. Sci. Transl. Med. 14, eabq4130 (2022). 35976993 10.1126/scitranslmed.abq4130 PMC10466502

[62] Accorsi EK, Britton A, Fleming-Dutra KE, Smith ZR, Shang N, Derado G, Miller J, Schrag SJ, Verani JR, Association Between 3 Doses of mRNA COVID-19 Vaccine and Symptomatic Infection Caused by the SARS-CoV-2 Omicron and Delta Variants. JAMA. 327, 639–651 (2022). 35060999 10.1001/jama.2022.0470 PMC8848203

[63] Tseng HF, Ackerson BK, Luo Y, Sy LS, Talarico CA, Tian Y, Bruxvoort KJ, Tubert JE, Florea A, Ku JH, Lee GS, Choi SK, Takhar HS, Aragones M, Qian L, Effectiveness of mRNA-1273 against SARS-CoV-2 Omicron and Delta variants. Nat. Med. (2022), doi:10.1038/s41591-022-01753-y. PMC9117141 35189624

[64] Pajon R, Doria-Rose NA, Shen X, Schmidt SD, O’Dell S, McDanal C, Feng W, Tong J, Eaton A, Maglinao M, Tang H, Manning KE, Edara V-V, Lai L, Ellis M, Moore KM, Floyd K, Foster SL, Posavad CM, Atmar RL, Lyke KE, Zhou T, Wang L, Zhang Y, Gaudinski MR, Black WP, Gordon I, Guech M, Ledgerwood JE, Misasi JN, Widge A, Sullivan NJ, Roberts PC, Beigel JH, Korber B, Baden LR, El Sahly H, Chalkias S, Zhou H, Feng J, Girard B, Das R, Aunins A, Edwards DK, Suthar MS, Mascola JR, Montefiori DC, SARS-CoV-2 omicron variant neutralization after mRNA-1273 booster vaccination. N. Engl. J. Med. 386, 1088–1091 (2022). 35081298 10.1056/NEJMc2119912 PMC8809504

[65] Bowen JE, Sprouse KR, Walls AC, Mazzitelli IG, Logue JK, Franko NM, Ahmed K, Shariq A, Cameroni E, Gori A, Bori A, Posavad CM, Dan JM, Zhang Z, Weiskopf D, Sette A, Crotty S, Iqbal NT, Corti D, Geffner J, Grifantini R, Chu HY, Veesler D, Omicron BA.1 and BA.2 neutralizing activity elicited by a comprehensive panel of human vaccines, doi:10.1101/2022.03.15.484542. PMC9348749 35857529

[66] Rössler A, Knabl L, von Laer D, Kimpel J, Neutralization profile after recovery from SARS-CoV-2 omicron infection. N. Engl. J. Med. (2022), doi:10.1056/NEJMc2201607. PMC9006769 35320661

[67] Lee I-J, Sun C-P, Wu P-Y, Lan Y-H, Wang I-H, Liu W-C, Tseng S-C, Tsung S-I, Chou Y-C, Kumari M, Chang Y-W, Chen H-F, Lin Y-S, Chen T-Y, Chiu C-W, Hsieh C-H, Chuang C-Y, Lin C-C, Cheng C-M, Lin H-T, Chen W-Y, Chiang P-C, Lee C-C, Liao JC, Wu H-C, Tao M-H, Omicron-specific mRNA vaccine induced potent neutralizing antibody against Omicron but not other SARS-CoV-2 variants. bioRxiv (2022), doi:10.1101/2022.01.31.478406.

[68] Richardson SI, Madzorera VS, Spencer H, Manamela NP, van der Mescht MA, Lambson BE, Oosthuysen B, Ayres F, Makhado Z, Moyo-Gwete T, Mzindle N, Motlou T, Strydom A, Mendes A, Tegally H, de Beer Z, Roma de Villiers T, Bodenstein A, van den Berg G, Venter M, de Oliviera T, Ueckermann V, Rossouw TM, Boswell MT, Moore PL, SARS-CoV-2 Omicron triggers cross-reactive neutralization and Fc effector functions in previously vaccinated, but not unvaccinated, individuals. Cell Host Microbe (2022), doi:10.1016/j.chom.2022.03.029. PMC8947963 35436444

[69] Stiasny K, Medits I, Springer D, Graninger M, Camp J, Höltl E, Aberle S, Traugott M, Hoepler W, Deutsch J, Lammel O, Borsodi C, Zoufaly A, Weseslindtner L, Aberle J, Puchhammer-St&#x00F6 E, Human primary Omicron BA.1 and BA.2 infections result in sub-lineage-specific neutralization (2022), doi:10.21203/rs.3.rs-1536794/v1. PMC9344875 35928813

[70] Walls AC, Miranda MC, Schäfer A, Pham MN, Greaney A, Arunachalam PS, Navarro M-J, Tortorici MA, Rogers K, O’Connor MA, Shirreff L, Ferrell DE, Bowen J, Brunette N, Kepl E, Zepeda SK, Starr T, Hsieh C-L, Fiala B, Wrenn S, Pettie D, Sydeman C, Sprouse KR, Johnson M, Blackstone A, Ravichandran R, Ogohara C, Carter L, Tilles SW, Rappuoli R, Leist SR, Martinez DR, Clark M, Tisch R, O’Hagan DT, Van Der Most R, Van Voorhis WC, Corti D, McLellan JS, Kleanthous H, Sheahan TP, Smith KD, Fuller DH, Villinger F, Bloom J, Pulendran B, Baric R, King NP, Veesler D, Elicitation of broadly protective sarbecovirus immunity by receptor-binding domain nanoparticle vaccines. Cell (2021), doi:10.1016/j.cell.2021.09.015. PMC8440233 34619077

[71] Cohen AA, Gnanapragasam PNP, Lee YE, Hoffman PR, Ou S, Kakutani LM, Keeffe JR, Wu H-J, Howarth M, West AP, Barnes CO, Nussenzweig MC, Bjorkman PJ, Mosaic nanoparticles elicit cross-reactive immune responses to zoonotic coronaviruses in mice. Science. 371, 735–741 (2021). 33436524 10.1126/science.abf6840 PMC7928838

[72] Chalkias S, Eder F, Essink B, Khetan S, Nestorova B, Feng J, Chen X, Chang Y, Zhou H, Montefiori D, Edwards DK, Girard B, Pajon R, Leav B, Walsh SR, Baden LR, Miller JM, Das R, Safety, immunogenicity and antibody persistence of a bivalent Beta-containing booster vaccine. Research Square (2022), doi:10.21203/rs.3.rs-1555201/v1. PMC9671805 36202997

[73] Cheng SSM, Mok CKP, Li JKC, Ng SS, Lam BHS, Jeevan T, Kandeil A, Pekosz A, Chan KCK, Tsang LCH, Ko FW, Chen C, Yiu K, Luk LLH, Chan KKP, Webby RJ, Poon LLM, Hui DSC, Peiris M, Plaque-neutralizing antibody to BA.2.12.1, BA.4 and BA.5 in individuals with three doses of BioNTech or CoronaVac vaccines, natural infection and breakthrough infection. J. Clin. Virol, 105273 (2022). 36081282 10.1016/j.jcv.2022.105273 PMC9428331

[74] Alsoussi WB, Malladi SK, Zhou JQ, Liu Z, Ying B, Kim W, Schmitz AJ, Lei T, Horvath SC, Sturtz AJ, McIntire KM, Evavold B, Han F, Scheaffer SM, Fox IF, Parra-Rodriguez L, Nachbagauer R, Nestorova B, Chalkias S, Farnsworth CW, Klebert MK, Pusic I, Strnad BS, Middleton WD, Teefey SA, Whelan SPJ, Diamond MS, Paris R, O’Halloran JA, Presti RM, Turner JS, Ellebedy AH, SARS-CoV-2 Omicron boosting induces de novo B cell response in humans. bioRxivorg (2022), doi:10.1101/2022.09.22.509040. 37011668

[75] Gaebler C, Wang Z, Lorenzi JCC, Muecksch F, Finkin S, Tokuyama M, Cho A, Jankovic M, Schaefer-Babajew D, Oliveira TY, Cipolla M, Viant C, Barnes CO, Bram Y, Breton G, Hägglöf T, Mendoza P, Hurley A, Turroja M, Gordon K, Millard KG, Ramos V, Schmidt F, Weisblum Y, Jha D, Tankelevich M, Martinez-Delgado G, Yee J, Patel R, Dizon J, Unson-O’Brien C, Shimeliovich I, Robbiani DF, Zhao Z, Gazumyan A, Schwartz RE, Hatziioannou T, Bjorkman PJ, Mehandru S, Bieniasz PD, Caskey M, Nussenzweig MC, Evolution of antibody immunity to SARS-CoV-2. Nature. 591, 639–644 (2021). 33461210 10.1038/s41586-021-03207-w PMC8221082

[76] Wang Z, Muecksch F, Schaefer-Babajew D, Finkin S, Viant C, Gaebler C, Hoffmann H-H, Barnes CO, Cipolla M, Ramos V, Oliveira TY, Cho A, Schmidt F, Da Silva J, Bednarski E, Aguado L, Yee J, Daga M, Turroja M, Millard KG, Jankovic M, Gazumyan A, Zhao Z, Rice CM, Bieniasz PD, Caskey M, Hatziioannou T, Nussenzweig MC, Naturally enhanced neutralizing breadth against SARS-CoV-2 one year after infection. Nature. 595, 426–431 (2021). 34126625 10.1038/s41586-021-03696-9 PMC8277577

[77] Pinto D, Sauer MM, Czudnochowski N, Low JS, Alejandra Tortorici M, Housley MP, Noack J, Walls AC, Bowen JE, Guarino B, Rosen LE, di Iulio J, Jerak J, Kaiser H, Islam S, Jaconi S, Sprugasci N, Culap K, Abdelnabi R, Foo C, Coelmont L, Bartha I, Bianchi S, Silacci-Fregni C, Bassi J, Marzi R, Vetti E, Cassotta A, Ceschi A, Ferrari P, Cippà PE, Giannini O, Ceruti S, Garzoni C, Riva A, Benigni F, Cameroni E, Piccoli L, Pizzuto MS, Smithey M, Hong D, Telenti A, Lempp FA, Neyts J, Havenar-Daughton C, Lanzavecchia A, Sallusto F, Snell G, Virgin HW, Beltramello M, Corti D, Veesler D, Broad betacoronavirus neutralization by a stem helix–specific human antibody. Science (2021), doi:10.1126/science.abj3321. PMC9268357 34344823

[78] Low JS, Jerak J, Tortorici MA, McCallum M, Pinto D, Cassotta A, Foglierini M, Mele F, Abdelnabi R, Weynand B, Noack J, Montiel-Ruiz M, Bianchi S, Benigni F, Sprugasci N, Joshi A, Bowen JE, Walls AC, Jarrossay D, Morone D, Paparoditis P, Garzoni C, Ferrari P, Ceschi A, Neyts J, Purcell LA, Snell G, Corti D, Lanzavecchia A, Veesler D, Sallusto F, ACE2 engagement exposes the fusion peptide to pan-coronavirus neutralizing antibodies. bioRxiv (2022), doi:10.1101/2022.03.30.486377. PMC9348755 35857703

[79] Marzi R, Bassi J, Silacci-Fregni C, Bartha I, Muoio F, Culap K, Sprugasci N, Lombardo G, Saliba C, Cameroni E, Cassotta A, Low JS, Walls AC, McCallum M, Tortorici MA, Bowen JE, Dellota EA, Dillen JR, Czudnochowski N, Pertusini L, Terrot T, Lepori V, Tarkowski M, Riva A, Biggiogero M, Pellanda AF, Garzoni C, Ferrari P, Ceschi A, Giannini O, Havenar-Daughton C, Telenti A, Arvin A, Virgin HW, Sallusto F, Veesler D, Lanzavecchia A, Corti D, Piccoli L, Maturation of SARS-CoV-2 Spike-specific memory B cells drives resilience to viral escape. bioRxivorg (2022), doi:10.1101/2022.09.30.509852. PMC9721160 36507220

[80] Chalkias S, Harper C, Vrbicky K, Walsh SR, Essink B, Brosz A, McGhee N, Tomassini JE, Chen X, Chang Y, Sutherland A, Montefiori DC, Girard B, Edwards DK, Feng J, Zhou H, Baden LR, Miller JM, Das R, A bivalent omicron-containing booster vaccine against covid-19. N. Engl. J. Med. 387, 1279–1291 (2022). 36112399 10.1056/NEJMoa2208343 PMC9511634

[81] Scheaffer SM, Lee D, Whitener B, Ying B, Wu K, Jani H, Martin P, Amato NJ, Avena LE, Berrueta DM, Schmidt SD, O’Dell S, Nasir A, Chuang G-Y, Stewart-Jones G, Koup RA, Doria-Rose NA, Carfi A, Elbashir SM, Thackray LB, Edwards DK, Diamond MS, Bivalent SARS-CoV-2 mRNA vaccines increase breadth of neutralization and protect against the BA.5 Omicron variant. bioRxiv (2022), doi:10.1101/2022.09.12.507614. PMC11752949 36265510

[82] Muik A, Lui BG, Bacher M, Wallisch A-K, Toker A, Couto CIC, Güler A, Mampilli V, Schmitt GJ, Mottl J, Ziegenhals T, Fesser S, Reinholz J, Wernig F, Schraut K-G, Hefesha H, Cai H, Yang Q, Walzer KC, Grosser J, Strauss S, Finlayson A, Krüger K, Ozhelvaci O, Grikscheit K, Kohmer N, Ciesek S, Swanson KA, Vogel AB, Türeci Ö, Sahin U, Exposure to BA.4/BA.5 Spike glycoprotein drives pan-Omicron neutralization in vaccine-experienced humans and mice (2022), doi:10.1101/2022.09.21.508818. PMC9765452 36378074

[83] Cohen AA, van Doremalen N, Greaney AJ, Andersen H, Sharma A, Starr TN, Keeffe JR, Fan C, Schulz JE, Gnanapragasam PNP, Kakutani LM, West AP, Saturday G, Lee YE, Gao H, Jette CA, Lewis MG, Tan TK, Townsend AR, Bloom JD, Munster VJ, Bjorkman PJ, Mosaic RBD nanoparticles protect against multiple sarbecovirus challenges in animal models. bioRxivorg (2022), doi:10.1101/2022.03.25.485875. PMC9273039 35857620

[84] Martinez DR, Schäfer A, Leist SR, De la Cruz G, West A, Atochina-Vasserman EN, Lindesmith LC, Pardi N, Parks R, Barr M, Li D, Yount B, Saunders KO, Weissman D, Haynes BF, Montgomery SA, Baric RS, Chimeric spike mRNA vaccines protect against Sarbecovirus challenge in mice. Science (2021), doi:10.1126/science.abi4506. PMC8899822 34214046

[85] Li D, Martinez DR, Schäfer A, Chen H, Barr M, Sutherland LL, Lee E, Parks R, Mielke D, Edwards W, Newman A, Bock KW, Minai M, Nagata BM, Gagne M, Douek D, DeMarco CT, Denny TN, Oguin TH, Brown A, Rountree W, Wang Y, Mansouri K, Edwards RJ, Ferrari G, Sempowski GD, Eaton A, Tang J, Cain DW, Santra S, Pardi N, Weissman D, Tomai M, Fox C, Moore IN, Andersen H, Lewis MG, Golding H, Khurana S, Seder R, Baric RS, Montefiori DC, Saunders KO, Haynes BF, Breadth of SARS-CoV-2 Neutralization and Protection Induced by a Nanoparticle Vaccine. bioRxiv (2022), doi:10.1101/2022.01.26.477915. PMC9588772 36274085

[86] Wang L-F, Tan CW, Chia WN, Zhu F, Young B, Chantasrisawad N, Hwa S-H, Yeoh AY-Y, Lim BL, Yap WC, Pada SK, Tan SY, Jantarabenjakul W, Chen S, Zhang J, Mah YY, Chen V, Chen M, Wacharapluesadee S, Team C-K, Putcharoen O, Lye D, Differential escape of neutralizing antibodies by SARS-CoV-2 Omicron and pre-emergent sarbecoviruses. Res Sq (2022), doi:10.21203/rs.3.rs-1362541/v1.

[87] Martinez DR, Schäfer A, Gobeil S, Li D, De la Cruz G, Parks R, Lu X, Barr M, Stalls V, Janowska K, Beaudoin E, Manne K, Mansouri K, Edwards RJ, Cronin K, Yount B, Anasti K, Montgomery SA, Tang J, Golding H, Shen S, Zhou T, Kwong PD, Graham BS, Mascola JR, Montefiori DC, Alam SM, Sempowski GD, Khurana S, Wiehe K, Saunders KO, Acharya P, Haynes BF, Baric RS, A broadly cross-reactive antibody neutralizes and protects against sarbecovirus challenge in mice. Sci. Transl. Med, eabj7125 (2021). 10.1126/scitranslmed.abj7125 PMC8899823 34726473

[88] Rappazzo CG, Tse LV, Kaku CI, Wrapp D, Sakharkar M, Huang D, Deveau LM, Yockachonis TJ, Herbert AS, Battles MB, O’Brien CM, Brown ME, Geoghegan JC, Belk J, Peng L, Yang L, Hou Y, Scobey TD, Burton DR, Nemazee D, Dye JM, Voss JE, Gunn BM, McLellan JS, Baric RS, Gralinski LE, Walker LM, Broad and potent activity against SARS-like viruses by an engineered human monoclonal antibody. Science. 371, 823–829 (2021). 33495307 10.1126/science.abf4830 PMC7963221

[89] Mao T, Israelow B, Suberi A, Zhou L, Reschke M, Peña-Hernández MA, Dong H, Homer RJ, Saltzman WM, Iwasaki A, Unadjuvanted intranasal spike vaccine booster elicits robust protective mucosal immunity against sarbecoviruses. bioRxiv (2022), doi:10.1101/2022.01.24.477597. PMC9798903 36302057

[90] Oh JE, Song E, Moriyama M, Wong P, Zhang S, Jiang R, Strohmeier S, Kleinstein SH, Krammer F, Iwasaki A, Intranasal priming induces local lung-resident B cell populations that secrete protective mucosal antiviral IgA. Science Immunology. 6, eabj5129 (2021). 34890255 10.1126/sciimmunol.abj5129 PMC8762609

[91] Langel SN, Johnson S, Martinez CI, Tedjakusuma SN, Peinovich N, Dora EG, Kuehl PJ, Irshad H, Barrett EG, Werts A, Tucker SN, Adenovirus type 5 SARS-CoV-2 vaccines delivered orally or intranasally reduced disease severity and transmission in a hamster model. Sci. Transl. Med, eabn6868 (2022). 35511920 10.1126/scitranslmed.abn6868 PMC9097881

[92] Hassan AO, Feldmann F, Zhao H, Curiel DT, Okumura A, Tang-Huau T-L, Case JB, Meade-White K, Callison J, Chen RE, Lovaglio J, Hanley PW, Scott DP, Fremont DH, Feldmann H, Diamond MS, A single intranasal dose of chimpanzee adenovirus-vectored vaccine protects against SARS-CoV-2 infection in rhesus macaques. Cell Rep Med. 2, 100230 (2021). 33754147 10.1016/j.xcrm.2021.100230 PMC7969912

[93] Lempp FA, Soriaga L, Montiel-Ruiz M, Benigni F, Noack J, Park Y-J, Bianchi S, Walls AC, Bowen JE, Zhou J, Kaiser H, Joshi A, Agostini M, Meury M, Dellota E Jr, Jaconi S, Cameroni E, Martinez-Picado J, Vergara-Alert J, Izquierdo-Useros N, Virgin HW, Lanzavecchia A, Veesler D, Purcell L, Telenti A, Corti D, Lectins enhance SARS-CoV-2 infection and influence neutralizing antibodies. Nature (2021), doi:10.1038/s41586-021-03925-1. 34464958

[94] Lan J, Ge J, Yu J, Shan S, Zhou H, Fan S, Zhang Q, Shi X, Wang Q, Zhang L, Wang X, Structure of the SARS-CoV-2 spike receptor-binding domain bound to the ACE2 receptor. Nature (2020), doi:10.1038/s41586-020-2180-5. 32225176

[95] McCulloch DJ, Kim AE, Wilcox NC, Logue JK, Greninger AL, Englund JA, Chu HY, Comparison of unsupervised home self-collected midnasal swabs with clinician-collected nasopharyngeal swabs for detection of SARS-CoV-2 infection. JAMA Netw. Open. 3, e2016382 (2020). 32697321 10.1001/jamanetworkopen.2020.16382 PMC7376392

[96] Weil AA, Luiten KG, Casto AM, Bennett JC, O’Hanlon J, Han PD, Gamboa LS, McDermot E, Truong M, Gottlieb GS, Acker Z, Wolf CR, Magedson A, Chow EJ, Lo NK, Pothan LC, McDonald D, Wright TC, McCaffrey KM, Figgins MD, Englund JA, Boeckh M, Lockwood CM, Nickerson DA, Shendure J, Bedford T, Hughes JP, Starita LM, Chu HY, Genomic surveillance of SARS-CoV-2 Omicron variants on a university campus. Nat. Commun. 13, 5240 (2022). 36068236 10.1038/s41467-022-32786-z PMC9446629

[97] Olmedillas E, Mann CJ, Peng W, Wang YT, Avalos RD, Structure-based design of a highly stable, covalently-linked SARS-CoV-2 spike trimer with improved structural properties and immunogenicity. bioRxiv (2021) (available at https://www.biorxiv.org/content/10.1101/2021.05.06.441046v1.abstract).

[98] Crawford KHD, Eguia R, Dingens AS, Loes AN, Malone KD, Wolf CR, Chu HY, Tortorici MA, Veesler D, Murphy M, Pettie D, King NP, Balazs AB, Bloom JD, Protocol and Reagents for Pseudotyping Lentiviral Particles with SARS-CoV-2 Spike Protein for Neutralization Assays. Viruses. 12 (2020), doi:10.3390/v12050513. PMC7291041 32384820

[99] Suloway C, Pulokas J, Fellmann D, Cheng A, Guerra F, Quispe J, Stagg S, Potter CS, Carragher B, Automated molecular microscopy: the new Leginon system. J. Struct. Biol. 151, 41–60 (2005). 15890530 10.1016/j.jsb.2005.03.010

[100] Tegunov D, Cramer P, Real-time cryo-electron microscopy data preprocessing with Warp. Nat. Methods. 16, 1146–1152 (2019). 31591575 10.1038/s41592-019-0580-y PMC6858868

[101] Punjani A, Rubinstein JL, Fleet DJ, Brubaker MA, cryoSPARC: algorithms for rapid unsupervised cryo-EM structure determination. Nat. Methods. 14, 290–296 (2017). 28165473 10.1038/nmeth.4169

[102] Zivanov J, Nakane T, Forsberg BO, Kimanius D, Hagen WJ, Lindahl E, Scheres SH, New tools for automated high-resolution cryo-EM structure determination in RELION-3. Elife. 7 (2018), doi:10.7554/eLife.42166. PMC6250425 30412051

[103] Punjani A, Zhang H, Fleet DJ, Non-uniform refinement: adaptive regularization improves single-particle cryo-EM reconstruction. Nat. Methods. 17, 1214–1221 (2020). 33257830 10.1038/s41592-020-00990-8

[104] Zivanov J, Nakane T, Scheres SHW, A Bayesian approach to beam-induced motion correction in cryo-EM single-particle analysis. IUCrJ. 6, 5–17 (2019). 10.1107/S205225251801463X PMC6327179 30713699

[105] Chen S, McMullan G, Faruqi AR, Murshudov GN, Short JM, Scheres SH, Henderson R, High-resolution noise substitution to measure overfitting and validate resolution in 3D structure determination by single particle electron cryomicroscopy. Ultramicroscopy. 135, 24–35 (2013). 23872039 10.1016/j.ultramic.2013.06.004 PMC3834153

[106] Rosenthal PB, Henderson R, Optimal determination of particle orientation, absolute hand, and contrast loss in single-particle electron cryomicroscopy. J. Mol. Biol. 333, 721–745 (2003). 14568533 10.1016/j.jmb.2003.07.013

[107] Pettersen EF, Goddard TD, Huang CC, Couch GS, Greenblatt DM, Meng EC, Ferrin TE, UCSF Chimera--a visualization system for exploratory research and analysis. J. Comput. Chem. 25, 1605–1612 (2004). 15264254 10.1002/jcc.20084

[108] Emsley P, Lohkamp B, Scott WG, Cowtan K, Features and development of Coot. Acta Crystallogr. D Biol. Crystallogr. 66, 486–501 (2010). 20383002 10.1107/S0907444910007493 PMC2852313

[109] Frenz B, Rämisch S, Borst AJ, Walls AC, Adolf-Bryfogle J, Schief WR, Veesler D, DiMaio F, Automatically Fixing Errors in Glycoprotein Structures with Rosetta. Structure. 27, 134–139.e3 (2019). 30344107 10.1016/j.str.2018.09.006 PMC6616339

[110] Wang RY, Song Y, Barad BA, Cheng Y, Fraser JS, DiMaio F, Automated structure refinement of macromolecular assemblies from cryo-EM maps using Rosetta. Elife. 5 (2016), doi:10.7554/eLife.17219. PMC5115868 27669148

[111] De Melo GD, Lazarini F, Levallois S, Hautefort C, Michel V, Larrous F, Verillaud B, Aparicio C, Wagner S, Gheusi G, Kergoat L, Kornobis E, Cokelaer T, Hervochon R, Madec Y, Roze E, Salmon D, Bourhy H, Lecuit M, Lledo P-M, COVID-19-associated olfactory dysfunction reveals SARS-CoV-2 neuroinvasion and persistence in the olfactory system. bioRxiv (2020), doi:10.1101/2020.11.18.388819.

[112] Boudewijns R, Thibaut HJ, Kaptein SJF, Li R, Vergote V, Seldeslachts L, Van Weyenbergh J, De Keyzer C, Bervoets L, Sharma S, Liesenborghs L, Ma J, Jansen S, Van Looveren D, Vercruysse T, Wang X, Jochmans D, Martens E, Roose K, De Vlieger D, Schepens B, Van Buyten T, Jacobs S, Liu Y, Martí-Carreras J, Vanmechelen B, Wawina-Bokalanga T, Delang L, Rocha-Pereira J, Coelmont L, Chiu W, Leyssen P, Heylen E, Schols D, Wang L, Close L, Matthijnssens J, Van Ranst M, Compernolle V, Schramm G, Van Laere K, Saelens X, Callewaert N, Opdenakker G, Maes P, Weynand B, Cawthorne C, Vande Velde G, Wang Z, Neyts J, Dallmeier K, STAT2 signaling restricts viral dissemination but drives severe pneumonia in SARS-CoV-2 infected hamsters. Nat. Commun. 11, 5838 (2020). 33203860 10.1038/s41467-020-19684-y PMC7672082

[113] Sanchez-Felipe L, Vercruysse T, Sharma S, Ma J, Lemmens V, Van Looveren D, Arkalagud Javarappa MP, Boudewijns R, Malengier-Devlies B, Liesenborghs L, Kaptein SJF, De Keyzer C, Bervoets L, Debaveye S, Rasulova M, Seldeslachts L, Li L-H, Jansen S, Yakass MB, Verstrepen BE, Böszörményi KP, Kiemenyi-Kayere G, van Driel N, Quaye O, Zhang X, Ter Horst S, Mishra N, Deboutte W, Matthijnssens J, Coelmont L, Vandermeulen C, Heylen E, Vergote V, Schols D, Wang Z, Bogers W, Kuiken T, Verschoor E, Cawthorne C, Van Laere K, Opdenakker G, Vande Velde G, Weynand B, Teuwen DE, Matthys P, Neyts J, Jan Thibaut H, Dallmeier K, A single-dose live-attenuated YF17D-vectored SARS-CoV-2 vaccine candidate. Nature. 590, 320–325 (2021). 33260195 10.1038/s41586-020-3035-9

[114] Kaptein SJF, Jacobs S, Langendries L, Seldeslachts L, Ter Horst S, Liesenborghs L, Hens B, Vergote V, Heylen E, Barthelemy K, Maas E, De Keyzer C, Bervoets L, Rymenants J, Van Buyten T, Zhang X, Abdelnabi R, Pang J, Williams R, Thibaut HJ, Dallmeier K, Boudewijns R, Wouters J, Augustijns P, Verougstraete N, Cawthorne C, Breuer J, Solas C, Weynand B, Annaert P, Spriet I, Vande Velde G, Neyts J, Rocha-Pereira J, Delang L, Favipiravir at high doses has potent antiviral activity in SARS-CoV-2-infected hamsters, whereas hydroxychloroquine lacks activity. Proc. Natl. Acad. Sci. U. S. A. 117, 26955–26965 (2020). 33037151 10.1073/pnas.2014441117 PMC7604414

[115] Corman VM, Landt O, Kaiser M, Molenkamp R, Meijer A, Chu DK, Bleicker T, Brünink S, Schneider J, Schmidt ML, Mulders DG, Haagmans BL, van der Veer B, van den Brink S, Wijsman L, Goderski G, Romette J-L, Ellis J, Zambon M, Peiris M, Goossens H, Reusken C, Koopmans MP, Drosten C, Detection of 2019 novel coronavirus (2019-nCoV) by real-time RT-PCR. Euro Surveill. 25 (2020), doi:10.2807/1560-7917.ES.2020.25.3.2000045. PMC6988269 31992387

[116] Lindenbach BD, Measuring HCV infectivity produced in cell culture and in vivo. Methods Mol. Biol. 510, 329–336 (2009). 19009272 10.1007/978-1-59745-394-3_24

[117] Reed LJ, Muench H, A SIMPLE METHOD OF ESTIMATING FIFTY PER CENT ENDPOINTS12. Am. J. Epidemiol. 27, 493–497 (1938).

[118] Starr TN, Greaney AJ, Hannon WW, Loes AN, Hauser K, Dillen JR, Ferri E, Farrell AG, Dadonaite B, McCallum M, Matreyek KA, Corti D, Veesler D, Snell G, Bloom JD, Shifting mutational constraints in the SARS-CoV-2 receptor-binding domain during viral evolution. bioRxiv (2022), doi:10.1101/2022.02.24.481899. PMC9273037 35762884

[119] Thomson EC, Rosen LE, Shepherd JG, Spreafico R, da Silva Filipe A, Wojcechowskyj JA, Davis C, Piccoli L, Pascall DJ, Dillen J, Lytras S, Czudnochowski N, Shah R, Meury M, Jesudason N, De Marco A, Li K, Bassi J, O’Toole A, Pinto D, Colquhoun RM, Culap K, Jackson B, Zatta F, Rambaut A, Jaconi S, Sreenu VB, Nix J, Zhang I, Jarrett RF, Glass WG, Beltramello M, Nomikou K, Pizzuto M, Tong L, Cameroni E, Croll TI, Johnson N, Di Iulio J, Wickenhagen A, Ceschi A, Harbison AM, Mair D, Ferrari P, Smollett K, Sallusto F, Carmichael S, Garzoni C, Nichols J, Galli M, Hughes J, Riva A, Ho A, Schiuma M, Semple MG, Openshaw PJM, Fadda E, Baillie JK, Chodera JD, ISARIC4C Investigators, COVID-19 Genomics UK (COG-UK) Consortium, Rihn SJ, Lycett SJ, Virgin HW, Telenti A, Corti D, Robertson DL, Snell G, Circulating SARS-CoV-2 spike N439K variants maintain fitness while evading antibody-mediated immunity. Cell. 184, 1171–1187.e20 (2021). 33621484 10.1016/j.cell.2021.01.037 PMC7843029

[120] Croll TI, ISOLDE: a physically realistic environment for model building into low-resolution electron-density maps. Acta Crystallogr D Struct Biol. 74, 519–530 (2018). 29872003 10.1107/S2059798318002425 PMC6096486

[121] Maier JA, Martinez C, Kasavajhala K, Wickstrom L, Hauser KE, Simmerling C, Ff14SB: Improving the accuracy of protein side chain and backbone parameters from ff99SB. J. Chem. Theory Comput. 11, 3696–3713 (2015). 26574453 10.1021/acs.jctc.5b00255 PMC4821407

[122] Kirschner KN, Yongye AB, Tschampel SM, González-Outeiriño J, Daniels CR, Foley BL, Woods RJ, GLYCAM06: a generalizable biomolecular force field. Carbohydrates. J. Comput. Chem. 29, 622–655 (2008). 17849372 10.1002/jcc.20820 PMC4423547

[123] Jorgensen WL, Chandrasekhar J, Madura JD, Impey RW, Klein ML, Comparison of simple potential functions for simulating liquid water. J. Chem. Phys. 79, 926–935 (1983).

[124] Joung IS, Cheatham TE 3rd, Determination of alkali and halide monovalent ion parameters for use in explicitly solvated biomolecular simulations. J. Phys. Chem. B. 112, 9020–9041 (2008). 18593145 10.1021/jp8001614 PMC2652252

[125] Roe DR, Cheatham TE 3rd, Parallelization of CPPTRAJ enables large scale analysis of molecular dynamics trajectory data. J. Comput. Chem. 39, 2110–2117 (2018). 30368859 10.1002/jcc.25382 PMC7313716

[126] Humphrey W, Dalke A, Schulten K, VMD: visual molecular dynamics. J. Mol. Graph. 14, 33–8, 27–8 (1996). 8744570 10.1016/0263-7855(96)00018-5

