# Supplementary material for: Imprinted antibody responses against SARS-CoV-2 Omicron sublineages
Source: Science. Author manuscript; Available in PMC 2026 Feb 26. (PMC12945441; doi:10.1126/science.adc9127)
Supplement: SuppMatt [file NIHMS2104669-supplement-SuppMatt.docx]

Supplementary Materials for

**Imprinted antibody responses against SARS-CoV-2 Omicron sublineages**

Young-Jun-Park^1,2^*, Dora Pinto^3^*, Alexandra C. Walls^1,2^*, Zhuoming Liu^4^*, Anna De Marco^3^, Fabio Benigni^3^, Fabrizia Zatta^3^, Chiara Silacci-Fregni^3^, Jessica Bassi^3^, Kaitlin R. Sprouse^1^, Amin Addetia^1^, John E. Bowen^1^, Cameron Stewart^1^, Martina Giurdanella^3^, Christian Saliba^3^, Barbara Guarino^3^, Michael A. Schmid^3^, Nicholas M. Franko^5^, Jennifer K. Logue^5^, Ha V. Dang^6^, Kevin Hauser^6^, Julia di Iulio^6^, William Rivera^6^, Gretja Schnell^6^, Anushka Rajesh^6^, Jiayi Zhou^6^, Nisar Farhat^6^, Hannah Kaiser^6^, Martin Montiel-Ruiz^6^, Julia Noack^6^, Florian A. Lempp^6^, Javier Janer^4^, Rana Abdelnabi^7^, Piet Maes^7^, Paolo Ferrari^8,9, 10^, Alessandro Ceschi^8,11,12,13^, Olivier Giannini^8,14^, Guilherme Dias de Melo^15^, Lauriane Kergoat^15^, Hervé Bourhy^15^, Johan Neyts^7^, Leah Soriaga^6^, Lisa A. Purcell^6^, Gyorgy Snell^6^, Sean P.J. Whelan^4^, Antonio Lanzavecchia^3^, Herbert W. Virgin^6,16,17^, Luca Piccoli^3^, Helen Y. Chu^5^, Matteo Samuele Pizzuto^3^, Davide Corti^3#^, and David Veesler^1,2#^

^1^Department of Biochemistry, University of Washington, Seattle, WA 98195, USA.

^2^Howard Hughes Medical Institute, University of Washington, Seattle, WA 98195, USA.

^3^Humabs Biomed SA, a subsidiary of Vir Biotechnology, 6500 Bellinzona, Switzerland.

^4^Department of Molecular Microbiology, Washington University School of Medicine, St. Louis, MO 63110, USA

^5^Division of Allergy and Infectious Diseases, University of Washington, Seattle, WA 98195, USA.

^6^Vir Biotechnology, San Francisco, CA 94158, USA

^7^KU Leuven Department of Microbiology, Immunology and Transplantation, Rega Institute for Medical Research, Laboratory of Virology and Chemotherapy, B-3000 Leuven, Belgium

^8^Faculty of Biomedical Sciences, Università della Svizzera italiana, Lugano, Switzerland.

^9^Division of Nephrology, Ente Ospedaliero Cantonale, Lugano, Switzerland.

^10^Clinical School, University of New South Wales, Sydney, New South Wales, Australia.

^11^Clinical Trial Unit, Ente Ospedaliero Cantonale, Lugano, Switzerland.

^12^Division of Clinical Pharmacology and Toxicology, Institute of Pharmacological Sciences of Southern Switzerland, Ente Ospedaliero Cantonale, Lugano, Switzerland.

^13^Department of Clinical Pharmacology and Toxicology, University Hospital Zurich, Zurich, Switzerland

^14^Department of Medicine, Ente Ospedaliero Cantonale, Bellinzona, Switzerland

^15^Institut Pasteur, Université de Paris Cité, Lyssavirus Epidemiology and Neuropathology Unit, Paris, F-75015, France

^16^Department of Pathology and Immunology, Washington University School of Medicine, St Louis, MO, USA

^17^Department of Internal Medicine, UT Southwestern Medical Center, Dallas, TX, USA

*These authors contributed equally

Correspondence: [dveesler@uw.edu](mailto:dveesler@uw.edu), [dcorti@vir.bio](mailto:dcorti@vir.bio)

**This PDF file includes:**

Materials and Methods

Fig S1 – S18

Table S1 – S9

**Materials and Methods**

**Cell lines**

Cell lines used in this study were obtained from ATCC (HEK293T and VeroE6), Thermo Fisher Scientific (Expi-CHO-S cells, FreeStyle 293-F cells and Expi293F cells) or Takara (Lenti-X 293T cells). VeroE6 cells stably expressing the TMPRSS2 protease (VeroE6-TMPRSS2 cells) were generated at Vir (*93*). None of the cell lines used were authenticated nor tested for mycoplasma contamination.

**Authentic SARS-CoV-2 strains**

SARS-CoV-2 isolates used in this study were obtained from BEI (WT: SARS-CoV-2 isolate USA-WA1/2020, BEI ref. NR-52281; BA.1: hCoV-19/USA/MD-HP20874/2021, BEI ref. NR-56461; BA.2: hCoV-19/USA/MD-HP24556/2022, BEI ref. NRS-56511; BA.4: hCoV-19/USA/MD-HP30386/2022, BEI ref. NRS-56803; BA.5: hCoV-19/USA/COR-22-063113/2022, BEI ref. NRS-58616); BA.2.12.1: USA/NY-MSHSPSPPV56475/2022, BEI ref. NR-56782 and propagated in house. The genomic sequences of all strains were confirmed by Sanger and NGS sequencing.

**Sample donors**

Blood samples were collected from participants as part of the Hospitalized or Ambulatory Adults with Respiratory Viral Infections (HAARVI) study and the Healthy Adult Specimen Repository study, which were approved by the University of Washington Human Subjects Division Institutional Review Board (STUDY00000959 and STUDY00002929). Baseline socio-demographic and clinical data for these individuals are summarized in Data S1. Midnasal swabs were self-collected with a Copan FLOQSwab and stored in 3mL of Copan Universal Viral Transport Medium (UTM). Infected participants were enrolled within 5 days of a positive RT-PCR test for SARS-CoV-2, an uninfected, vaccinated cohort was simultaneously enrolled. Consent and baseline questionnaires were completed electronically, and collection kits were sent for remote self-collection (*95*). Completed swab kits were transported to the lab same-day by courier, and aliquoted and heat inactivated before storage at -80˚C. Acutely ill individuals collected 8-9 swabs over 14 days post enrollment, controls completed 2 swabs at days 1 and 14 post enrollment. Samples were obtained from SARS-CoV-2 convalescent and vaccinated individuals under study protocols approved by the local institutional review boards (Canton Ticino Ethics Committee, Switzerland, Comitato Etico Milano Area 1). All donors provided written informed consent for the use of blood and blood derivatives (such as peripheral blood mononuclear cells, sera or plasma) for research.

**Viral sequencing for Breakthrough cases**

Sequencing of SARS-CoV-2 variants was performed as previously described (*19*, *96*).

**Recombinant Protein Expression and purification for nasal swab ELISAs**

The SARS-CoV-2 S VFLIP ectodomain (*97*) was produced in Expi293F Cells (ThermoFisher Scientific) grown in suspension using Expi293 Expression Medium (ThermoFisher Scientific) at 37°C in a humidified 8% CO_2_ incubator with constant rotation at 130 rpm. Cells grown to a density of 3 million cells per mL were transfected using the ExpiFectamine 293 Transfection Kit (ThermoFisher Scientific) and cultivated for four days. SARS-CoV-2 S VFLIP was purified from the clarified supernatant using a HisTrapHP column (Cytiva) and washed with 10 column volumes of 25 mM sodium phosphate pH 8.0 and 150 mM NaCl before elution on a gradient up to 500 mM imidazole. Purified protein was buffer exchanged into 20 mM Tris-HCl pH 8.0 and 100 mM NaCl, concentrated using 100 kDa MWCO centrifugal filters (Amicon Ultra) to 1-2 mg/mL and flash frozen.

**Antigen-specific memory B cell repertoire analysis (AMBRA) of secreted IgGs**

Replicate cultures of total unfractionated PBMCs obtained from SARS-CoV-2 infected and/or vaccinated individuals were seeded in 96 U-bottom plates (Corning) in RPMI1640 supplemented with 10% Hyclone, sodium pyruvate, MEM non-essential amino acids, stable glutamine and Penicillin-Streptomycin. Memory B cell stimulation and differentiation was induced by adding 2.5 μg/ml R848 (3 M) and 1000 U/ ml human recombinant IL-2 at 37 °C and 5% CO_2_. After 10 days, the cell culture supernatants were collected for ELISA analysis.

**Analysis of IgGs secreted by plasma cells**

Plasma cells from peripheral blood were stained with PE-conjugated anti-CD138 (BD-Pharmingen), enriched by magnetic separation with anti-PE microbeads (Miltenyi) and finally purified by cell sorting with a Sony cell sorter. Cells were seeded at 0.5 cell/well in multiple 384 well plates in 50 μl complete RPMI 1640 medium supplemented with 10% FCS (Hyclone) and 10 ng/ml human r-IL6 (R&D). After three days, 20 μl of culture supernatants were collected using an automated liquid handling equipment (Perkin Elmer) and tested in parallel micro-ELISA (5 μl per test) for the presence of IgG antibodies recognizing SARS-CoV-2 variant RBDs. PBS was used as negative control.

**Generation of VSV pseudovirus**. For analysis described in Figure 1A, D, and E, SARS-CoV-2 G614 S (D614G) with C terminal 21 residues deleted, Delta S (mutations T19, G142D, E156G, 157-158del, T478K, D614G, D950N) with C terminal 21 residues deleted, BA.1 S (A67V, del69/70, T95I, G142D, del143/145, del211, L212I, ins214EPE, G339D, S371L, S373P, S375F, K417N, N440K, G446S, S477N, T478K, E484A, Q493R, G496S, Q498R, N501Y, Y505H, T547K, D614G, H655Y, N679K, P681H, N764K, D796Y, N856K, Q954H, N969K L981F) with C terminal 21 residues deleted, BA.2 S (T19I, L24S, del25/27, G142D, V213G, G339D, S371F, S373P, S375F, T376A, D405N, R408S, K417N, N440K, S477N, T478K, E484A, Q493R, Q498R, N501Y, Y505H, D614G, H655Y, N679K, P681H, N764K, D796Y, Q954H, N969K) with C terminal 21 residues deleted, BA.2.12.1 S (T19I, L24S, del25/27, G142D, V213G, G339D, S371F, S373P, S375F, T376A, D405N, R408S, K417N, N440K, L452Q, S477N, T478K, E484A, Q493R, Q498R, N501Y, Y505H, D614G, H655Y, N679K, P681H, S704L, N764K, D796Y, Q954H, N969K) with C terminal 21 residues deleted, BA.4/5 S (T19I, L24S, del25/27, del69/70, G142D, V213G, G339D, S371F, S373P, S375F, T376A, D405N, R408S, K417N, N440K, L452R, S477N, T478K, E484A, F486V, Q498R, N501Y, Y505H, D614G, H655Y, N679K, P681H, N764K, D796Y, Q954H, N969K) with C terminal 21 residues deleted, and SARS-CoV S with C terminal 21 residues deleted pseudotyped VSV viruses were prepared as described previously (*98*). Briefly, HEK293T cells in DMEM supplemented with 10% FBS, 1% PenStrep seeded in 10-cm dishes were transfected with the plasmid encoding for the corresponding S glycoprotein using lipofectamine 2000 (Life Technologies) following the manufacturer’s instructions. One day post-transfection, cells were infected with VSV(G^∗^ΔG-luciferase) and after 2 hours were washed five times with DMEM before adding medium supplemented with anti-VSV-G antibody (I1- mouse hybridoma supernatant, CRL- 2700, ATCC). Virus pseudotypes were harvested 18-24 h post-inoculation, clarified by centrifugation at 2,500 x g for 10 minutes, filtered through a 0.45 μm cut off membrane, concentrated 10 fold with a 30 kDa cut off membrane, aliquoted and stored at -80°C.

All other analysis proceeded as follows: replication defective VSV pseudovirus expressing SARS-CoV-2 spike proteins corresponding to the ancestral Wuhan-Hu-1 virus and the VOCs were generated as previously described with some modifications (*3*). Lenti-X 293T cells (Takara) were seeded in 15-cm^2^ dishes at a density of 10 × 10^6^ cells per dish and the following day were transfected with 25 µg of S expression plasmid with TransIT-Lenti (Mirus, 6600) according to the manufacturer’s instructions. One day after transfection, cells were infected with VSV-luc (VSV-ΔG) with a multiplicity of infection (MOI) of 3 for 1 h, rinsed three times with PBS containing Ca^2+^ and Mg^2+^, then incubated for an additional 24 h in complete medium at 37 °C. The cell supernatant was clarified by centrifugation, aliquoted, and frozen at −80 °C. For pseudoviruses expressing S substitutions that resulted in decreased infectivity using Vero E6 cells, Vero E6-TMPRSS2 cells were substituted as target cells to determine pseudovirus titers. S expression plasmids used for the generation of VSV pseudoviruses harbored a a C-terminal 19 residue deletion and the following mutations: BA.1: A67V, Δ69-70, T95I, G142D, Δ143-145, Δ211, L212I, ins214EPE, G339D, S371L, S373P, S375F, K417N, N440K, G446S, S477N, T478K, E484A, Q493R, G496S, Q498R, N501Y, Y505H, T547K, D614G, H655Y, N679K, P681H, N764K, D796Y, N856K, Q954H, N969K, L981F; BA.2: T19I, L24-, P25-, P26-, A27S, G142D, V213G, G339D, S371L, S373P, S375F, D405N, R408S, K417N, N440K, S477N, T478K, E484A, Q493R, Q498R, N501Y, Y505H, D614G, H655Y, N679K, P681H, N764K, D796Y/ N856K/ Q954H/ N969K; BA.3: A67V, H69-, V70-, T95I, G142D, V143-, Y144-, Y145-, N211-, L212I, G339D, S371F, S373P S375F, D405N, K417N, N440K, G446S, S477N, T478K, E484A, Q493R, Q498R, N501Y, Y505H, D614G, H655Y, N679K, P681H, N764K, D796Y, Q954H, N969K; BA.4-N658S: T19I, L24-, P25-, P26-, A27S, G142D, V213G, Δ69-70, G339D, S371F, S373P, S375F, T376A, D405N, R408S, K417N, N440K, L452R, S477N, T478K, E484A, F486V, Q498R, N501Y, Y505H, D614G, H655Y, N658S, N679K, P681H, N764K, D796Y, Q954H, N969K; BA.4-V3G: V3G, T19I, L24-, P25-, P26-, A27S, G142D, V213G, Δ69-70, G339D, S371F, S373P, S375F, T376A, D405N, R408S, K417N, N440K, L452R, S477N, T478K, E484A, F486V, Q498R, N501Y, Y505H, D614G, H655Y, N679K, P681H, N764K, D796Y, Q954H, N969K; BA.5: T19I, L24-, P25-, P26-, A27S, G142D, V213G, Δ69-70, G339D, S371F, S373P, S375F, T376A, D405N, R408S, K417N, N440K, L452R, S477N, T478K, E484A, F486V, Q498R, N501Y, Y505H, D614G, H655Y, N679K, P681H, N764K, D796Y, Q954H, N969K; BA.2.12.1: T19I, L24-, P25-, P26-, A27S, G142D, V213G, G339D, S371F, S373P, S375F, T376A, D405N, R408S, K417N, N440K, L452Q, S477N, T478K, E484A, Q493R, Q498R, N501Y, Y505H, D614G, H655Y, N679K, P681H, S704L, N764K, D796Y, Q954H, N969K. BA.2.75: T19I, L24-, P25-, P26-, A27S, G142D, K147E, W152R, F157L, I210V, V213G, G257S, G339H, S371F, S373P, S375F, T376A, D405N, R408S, K417N, N440K, G446S, N460K, S477N, T478K, E484A, Q498R, N501Y, Y505H, D614G, H655Y, N679K, P681H, N764K, D796Y, Q954H, N969K.

**VSV pseudovirus neutralization**. For the analyses described in Figure 1A, D, and E, VeroE6-TMPRSS2 (*93*) cells were grown in DMEM supplemented with 10% FBS, 1% PenStrep, and 8 μg/mL puromycin (to ensure retention of TMPRSS2) with 5% CO_2_ in a 37°C incubator (ThermoFisher). Cells were trypsinized using 0.05% trypsin and plated to 40,000 cells/well. 16 hours later cells were checked to be at 80% confluence. In an empty half-area 96-well plate, a 1:3 serial dilution of sera was made in DMEM and diluted pseudovirus was then added and incubated at room temperature for 30-60 min before addition of the sera-virus mixture to the VeroE6-TMPRSS2 cells at 37°C. 2 hours later, 40 μL of a DMEM solution containing 20% FBS and 2% PenStrep was added to each well. After 17-20 hours, 40 μL/well of One-Glo-EX substrate (Promega) was added to the cells and incubated in the dark for 5-10 min prior to reading on a BioTek plate reader. Measurements were done at least in duplicates using two distinct batches of pseudoviruses and one representative experiment is shown. Relative luciferase units were plotted and normalized in Prism (GraphPad). Nonlinear regression of log(inhibitor) versus normalized response was used to determine IC_50_ values from curve fits.

For all other analyses, VeroE6 cells were grown in DMEM supplemented with 10% FBS and seeded into clear bottom white 96 well plates (PerkinElmer, 6005688) at a density of 20,000 cells per well. The next day, monoclonal antibodies were serially diluted in pre-warmed complete medium, mixed with pseudoviruses and incubated for 1 h at 37 °C in round bottom polypropylene plates. Medium from cells was aspirated and 50 µl of virus–monoclonal antibody complexes were added to cells, which were then incubated for 1 h at 37 °C. An additional 100 µl of pre-warmed complete medium was then added on top of complexes and cells were incubated for an additional 16–24 h. Conditions were tested in duplicate wells on each plate and eight wells per plate contained untreated infected cells (defining the 0% of neutralization, ‘MAX RLU’ value) and infected cells in the presence of S309 and S2X259v50 (affinity-matured version of S2X259) at 20 µg/ml each (defining the 100% of neutralization, ‘MIN RLU’ value). For sotrovimab experiments, conditions were tested in triplicate wells starting at 10 µg/ml sotrovimab; cells only and untreated infected cells were included on each plate. Virus–monoclonal antibody-containing medium was then aspirated from cells and 100 µl of a 1:2 dilution of SteadyLite Plus (PerkinElmer, 6066759) in PBS with Ca^2+^ and Mg^2+^ was added to cells. Plates were incubated for 15 min at room temperature and then analyzed on the Synergy-H1 (Biotek). The average relative light units (RLUs) of untreated infected wells (MAX RLUave) were subtracted by the average of MIN RLU (MIN RLUave) and used to normalize percentage of neutralization of individual RLU values of experimental data according to the following formula: (1 − (RLUx – MIN RLUave)/ (MAX RLUave – MIN RLUave)) × 100. Data were analyzed with Prism (v.9.1.0). IC_50_ values were calculated from the interpolated value from the log(inhibitor) versus response, using variable slope (four parameters) nonlinear regression with an upper constraint of ≤100, and a lower constrain equal to 0. Each neutralization experiment was conducted as two independent experiments – that is, biological replicates – in which each biological replicate contains a technical duplicate or triplicate. IC_50_ values across biological replicates are presented as geometric mean ± s.d. For sotrovimab, IC_50_ values were calculated as described above using an upper constraint of <100. For pseudoviruses expressing spike substitutions that resulted in decreased infectivity using VeroE6 cells, Vero E6-TMPRSS2 cells were substituted as target cells for neutralization assays.

**Neutralization of authentic SARS-CoV-2 viruses**

Vero-TMPRSS2 cells were seeded into black-walled, clear-bottom 96-well plates at 2×10^4^ cells/well and cultured overnight at 37°C. The next day, 9-point 4-fold serial dilutions of mAbs were prepared in growth media (DMEM + 10% FBS). The different SARS-CoV-2 strains were diluted in infection media (DMEM + 2% BSA) at a final MOI of 0.01 PFU/cell, added to the mAb dilutions and incubated for 30 min at 37°C. Media was removed from the cells, mAb-virus complexes were added and incubated at 37°C for 18h (WA-1), 21±3h (BA.4, BA.5), 24h (BA.2, BA.2.12.1) or 30h (BA.1). Cells were fixed with 4% PFA (Electron Microscopy Sciences, #15714S), permeabilized with Triton X-100 (SIGMA, #X100-500ML) and stained with an antibody against the viral nucleocapsid protein (Sino Biologicals, #40143-R001) followed by a staining with the nuclear dye Hoechst 33342 (Fisher Scientific, # H1399) and a goat anti-rabbit Alexa Fluor 647 antibody (Invitrogen, #A-21245). Plates were imaged on a Cytation5 plate reader. Whole well images were acquired (12 images at 4X magnification per well) and nucleocapsid-positive cells were counted using the manufacturer’s software.

**Enzyme-linked immunosorbent assay (ELISA)**

Ninety-six half area well-plates (Corning, 3690) were coated overnight at 4 °C with 25 μl of sarbecovirus RBD proteins of SARS-CoV-2 (YP_009724390.1), RaTG13 (QHR63300.2), Pangolin_Guangdong-2019 (EPI_ISL_410721), Pangolin_Guangxi (QIA48623.1), SARS-CoV Urbani (AAP13441.1) and WIV1 (AGZ48831.1), prepared at 5 μg/ml in PBS pH 7.2. Plates were then blocked with PBS 1% BSA (Sigma-Aldrich, A3059) and subsequently incubated with mAb serial dilutions for 1 h at room temperature. After 4 washing steps with PBS 0.05% Tween 20 (PBS-T) (Sigma-Aldrich, 93773), goat anti-human IgG secondary antibody (Southern Biotech, 2040-04) was added and incubated for 1 h at room temperature. Plates were then washed four times with PBS-T and 4-nitrophenyl phosphate (pNPP, Sigma-Aldrich, 71768) substrate was added. After 30 min incubation, absorbance at 405 nm was measured by a plate reader (Biotek) and data were plotted using Prism GraphPad 9.1.0.

**Nasal Swab ELISA**

For anti-S ELISA, 50 μL of 2 μg/mL SARS-CoV-2 S VFLIP was plated onto 384-well Nunc Maxisorp (ThermoFisher) plates in PBS and sealed overnight at room temperature. The next day plates were washed 4 × in Tris Buffered Saline Tween (TBST- 20mM Tris pH 8, 150mM NaCl, 0.1% Tween) using a plate washer (BioTek) and blocked with Casein (ThermoFisher) for 1 h at 37°C. Plates were washed 4 × in TBST and 1:5 serial dilutions of nasal swab in universal transport media were made in 50 μL TBST and incubated at 37°C for 1 h. Plates were washed 4 × in TBST, then anti-human (Invitrogen) horseradish peroxidase-conjugated antibodies were diluted 1:5,000 and 50 μL added to each well and incubated at 37°C for 1 h. Plates were washed 4 × in TBST and 50 μL of TMB (SeraCare) was added to every well for 3 min at room temperature. The reaction was quenched with the addition of 50 μL of 1 N HCl. Plates were immediately read at 450 nm on a Biotek Neo2 plate reader and data plotted and fit in Prism (GraphPad) using nonlinear regression sigmoidal, 4PL, X is log(concentration) to determine EC_50_ values from curve fits. For curves that did not reach an OD450 of 4, a constraint of OD450 4 was placed on the upper bounds of the fit.

**AMBRA ELISA**

Spectraplate-384 with high protein binding treatment (custom made from Perkin Elmer) were coated overnight at 4°C with 1 µg/ml of RBD (produced in house), SARS-CoV RBD (produced in house), Omicron BA.1, BA.2 and BA.4/5 RBD (produced in house) in PBS pH 7.2 or PBS alone as control. Plates were subsequently blocked with Blocker Casein (1%) in PBS (Thermo Fisher Scientific, 37528) supplemented with 0.05% Tween 20 (Sigma Aldrich, 93773-1KG). The coated plates were incubated with diluted B cell supernatant for 1h at RT. Plates were washed with PBS containing 0.05 % Tween20 (PBS-T), and binding was revealed using secondary goat anti-human IgG-AP (Southern Biotech, 2040-04). After washing, pNPP substrate (Sigma-Aldrich, 71768-25G) was added and plates were read at 405 nm after 1 h or 30 minutes.

**Recombinant protein production for SPR binding assays and AMBRA ELISA**

SARS-CoV-2 RBD constructs contained residues 328–531 of the spike protein from GenBank NC_045512.2 with an N-terminal signal peptide and a C-terminal thrombin cleavage site-Twin-Strep-8×His-tag. For SPR binding assays, proteins were expressed in Expi293F cells (Thermo Fisher Scientific) at 37 °C and 8% CO2. Transfections were performed using the ExpiFectamine 293 Transfection Kit (Thermo Fisher Scientific). Cell culture supernatants were collected three to five days after transfection and supplemented with 10x PBS to a final concentration of 2.5x PBS (342.5 mM NaCl, 6.75 mM KCl and 29.75 mM phosphates). SARS-CoV-2 RBDs were purified using a Cobalt affinity column (HisTALON Superflow column from Takara or HiTrap TALON crude column from Cytiva) followed by buffer exchange into PBS using a HiPrep 26/10 desalting column (Cytiva) or, for the Omicron BA.1 and BA.2 RBDs, a Superdex 200 Increase 10/300 GL column (Cytiva).

**Surface plasmon resonance (SPR) assays to measure binding of the S2X324 Fab to RBDs**

Measurements were performed using a Biacore T200 instrument. A CM5 chip with covalently immobilized StrepTactin XT was used for surface capture of Twin-Strep Tag-containing RBDs. Running buffer was HBS-EP+ pH 7.4 (Cytiva) and measurements were performed at 25 ̊C. Experiments were performed with a 3-fold dilution series of monomeric S2X324 Fab at 300, 100, 33 and 11 nM and were run as single-cycle kinetics. Data were double reference-subtracted and fit to a binding model using Biacore Evaluation software. The 1:1 binding model was used to estimate the kinetics parameters. The experiment was performed twice with two biological replicates for each ligand (RBDs). K_D_ values are reported as the average of two replicates with the corresponding standard deviation.

**Competition assays by biolayer interferometry**

Biolayer interferometry was used to assess S2X324 competition with S2K146 and sotrovimab using an Octet HTX (Sartorius). All reagents were prepared in kinetics buffer (PBS 0.01% BSA) at the indicated concentrations. His-tagged SARS-CoV-2 RBD was prepared at 8 μg/ml and loaded on prehydrated anti-penta-HIS biosensors (Sartorius) for 3 min. Biosensors were then moved into a solution containing S2X324 mAb (20 μg/ml) and association was recorded for 7 min. A second association step was subsequently performed into S2K146, S2H14, S2X259, S304 and S309 mAbs solutions at 20 μg/ml while maintaining S2X324 at a constant concentration of 20 μg/ml and recorded for 7 min. Association with S2X324 was used as positive control. Response values were exported and plotted using GraphPad Prism 9.1.0.

**Blockade of SARS-CoV-2 binding to ACE2**

SARS-CoV-2 mouse/rabbit Fc-tagged RBDs (final concentration 20 ng/ml) were incubated with serially diluted recombinant mAbs (from 25 µg/ml) and incubated for 1 h 37°C. The complex RBD:mAbs was then added to a pre-coated hACE2 (2 µg/ml in PBS) 96-well plate MaxiSorp (Nunc) and incubated 1 hour at room temperature. Subsequently, the plates were washed, and a goat anti-mouse/rabbit IgG (Southern Biotech) coupled to alkaline phosphatase (Jackson Immunoresearch) added to detect mouse Fc-tagged RBDs binding. After further washing, the substrate (p-NPP, Sigma) was added, and plates read at 405 nm using a microplate reader (Biotek). The percentage of inhibition was calculated as follow: (1-((OD sample-OD neg ctr)/(OD pos. ctr-OD neg. ctr))*100. Blockade of binding by AMBRA supernatants was determined for OD values <2.

**Blockade of mAb binding to RBD**

Anti-RBD site-specific mAbs S2E12, S2X324, S2X259, S309 (parent antibody of sotrovimab) and S2H97 were produced with a hamster Fc and added at 20 µg/ml (final concentration) at room temperature for 30 min to ELISA 384-well shallow plates (Perkin Elmer) previously coated overnight at 4°C with 1 µg/ml RBD in PBS and blocked in Blocker Casein (1%) in PBS. Controls wells without mAbs were included. After 30 min, AMBRA supernatants were added at room temperature for 30 min to both wells with and without mAbs. Plates were washed and antibody binding was revealed using 1/2000 alkaline phosphatase-conjugated anti human-IgG Fc-specific antibody (Jackson ImmunoResearch, 109-055-098) for 45 min. After washing, pNPP substrate (Sigma-Aldrich) was added and plates were read at 405 nm after 1 h. Blockade of binding by a site-specific mAb was determined when a 2-fold reduction of OD values in the wells with the mAbs was observed when compared to the corresponding OD values in the well without the mAbs.

**Cell-surface mAb-mediated S_1_ shedding**

CHO cells stably expressing the prototypic SARS-CoV-2 S were harvested, washed in wash buffer (PBS 1% BSA 2 mM EDTA) and resuspended in PBS. Cells were then counted and 90,000 cells/well were dispensed into a round-bottom 96 well plate (Corning) to be treated with 10 µg/ml TPCK-Trypsin (Worthington Biochem) for 30 min at 37°C. After a washing step, cells were incubated with 15 µg/ml mAbs solution for 180, 120, 60, 30 or 5 min at 37°C. The Mcrb17 mAb, which binds the S_2_ subunit in the pre- and post- fusion conformation, was included to rule out S internalization over time. After the incubation for the allotted time, cells were washed with ice-cold wash buffer and stained with 1.5 µg/ml Alexa Fluor647-labeled Goat Anti-Human IgG secondary Ab (Jackson Immunoresearch) for 30 min on ice in the dark. Cells were then washed twice with cold wash buffer and analyzed using a ZE5 cytometer (Biorad) with acquisition chamber T= 4°C. Binding at each time point (MFI) was determined normalizing to the MFI at 5 minutes time point and data plotted using GraphPad Prism v. 9.1.1

**Negative-stain electron microscopy imaging of refolding of soluble S trimers**

The SARS-CoV-2 S ectodomain trimer (residues 14-1221) containing the D614G mutation, a mu-phospohatase signal peptide, and a mutated S1/S2 cleavage site (SGAR) followed by a TEV cleavage site, a fold-on timerization motif, and an 8x His tag was recombinantly expressed in Expi293 cells and purified as previously described (*93*). 1 µM of S protein was incubated with 1.5 µM of Fab for 1 hour at room temperature. The S-Fab complexes were diluted to 0.01 mg/mL and added to freshly glow-discharged carbon-coated copper grids and stained with 2% uranyl formate. Data were acquired using a 120 kV FEI Tecnai G2 Spirit with a Gatan Ultrascan 4000 4k x 4k CCD camera at 67,000x nominal magnification using Leginon (*99*). The defocus ranged from 1.0 to 2.0 µm and the pixel size was 1.6 Å.

**CryoEM sample preparation, data collection and data processing**

Recombinantly expressed and purified S2X324 Fab and SARS-CoV-2 Omicron BA.1 S (*18*) were incubated at 1 mg/ml (for UltraAuFoil grids) or 0.1mg/ml (for lacey thin carbon grids) with a 1.2 molar excess of S2X324 Fab at 4°C for 1 hr. Three microliters of the complex mixture were loaded onto freshly glow discharged R 2/2 UltrAuFoil grids (200 mesh) or lacey grids covered with a thin layer of home-made carbon, prior to plunge freezing using a Vitrobot MarkIV (ThermoFisher Scientific) with a blot force of 0 and 6 sec blot time (for the UltrAuFoil grids) or with a blot force of -1 and 3 sec blot time (for the lacey thin carbon grids) at 100 % humidity and 22°C.

Data were acquired using both types of grids on an FEI Titan Krios transmission electron microscope operated at 300 kV and equipped with a Gatan K3 direct detector and Gatan Quantum GIF energy filter, operated in zero-loss mode with a slit width of 20 eV. Automated data collection was carried out using Leginon (*99*) at a nominal magnification of 105,000x with a pixel size of 0.843 Å. The dose rate was adjusted to 15 counts/pixel/s, and each movie was acquired in super-resolution mode fractionated in 75 frames of 40 ms. 10,842 micrographs were collected with a defocus range comprised between -0.5 and -2.5 μm. Movie frame alignment, estimation of the microscope contrast-transfer function parameters, particle picking, and extraction were carried out using Warp (*100*).

Two rounds of reference-free 2D classification were performed using CryoSPARC (*101*) to select well-defined particle images. These selected particles were subjected to two rounds of 3D classification with 50 iterations each (angular sampling 7.5˚ for 25 iterations and 1.8˚ with local search for 25 iterations), using our previously reported closed SARS-CoV-2 S structure as initial model (PDB 6VXX) (*46*) using Relion (*102*). 3D refinements were carried out using non-uniform refinement along with per-particle defocus refinement in CryoSPARC (*103*). Selected particle images were subjected to the Bayesian polishing procedure (*104*) implemented in Relion3.0 before performing another round of non-uniform refinement in CryoSPARC followed by per-particle defocus refinement and again non-uniform refinement. To improve the density of the S/S2X324 interface, the particles from the non-uniform refinement were subjected to focused 3D classification without refining angles and shifts using a soft mask on the closed RBD and bound S2X324 variable domains with a tau value of 60 in Relion. Particles belonging to classes with the best resolved local density were selected and subjected to local refinement using CryoSPARC. Local resolution estimation, filtering, and sharpening were carried out using CryoSPARC. Reported resolutions are based on the gold-standard Fourier shell correlation (FSC) of 0.143 criterion and Fourier shell correlation curves were corrected for the effects of soft masking by high-resolution noise substitution (*105*, *106*).

**Model building and refinement**

UCSF Chimera (*107*) and Coot (*108*) were used to fit atomic models into the cryoEM map. The Omicron BA.1 S/S2X324 and RBD/S2X324 models were refined and relaxed using Rosetta using sharpened and unsharpened maps (*109*, *110*).

**Selection of SARS-CoV-2 monoclonal antibody escape mutants**

A VSV-SARS-CoV-2 Wuhan-Hu-1 D614G S and Omicron BA.1 S chimera were used to select for mAb resistant mutants, as previously described (*35*). Briefly, mutants were recovered by plaque isolation on VeroE6 cells with the indicated mAb in the overlay. The concentration of mAb in the overlay was determined by neutralization assays at a multiplicity of infection (MOI) of 100. Escape clones were plaque-purified on Vero E6 cells in the presence of mAb, and plaques in agarose plugs were amplified on MA104 cells with the mAb present in the medium. Viral stocks were amplified on MA104 cells at an MOI of 0.01 in Medium 199 containing 2% FBS and 20 mM HEPES pH 7.7 (Millipore Sigma) at 34°C. Viral supernatants were harvested upon extensive cytopathic effect and clarified of cell debris by centrifugation at 1,000 x g for 5 min. Aliquots were maintained at -80°C. Viral RNA was extracted from VSV-SARS-CoV-2 S mutant viruses using RNeasy Mini kit (Qiagen), and the S gene was amplified using OneStep RT-PCR Kit (Qiagen). The mutations were identified by Sanger sequencing (GENEWIZ). Their resistance was verified by subsequent virus infection in the presence or absence of mAb. VeroE6 cells were seeded into 12 well plates overnight. The virus was serially diluted using DMEM and cells were infected at 37°C for 1 h. Cells were cultured with an agarose overlay in the presence or absence of mAb at 34°C for 2 days. Plates were scanned on a biomolecular imager and expression of eGFP monitored at 48 hours post-infection.

**Viral replication fitness assays**

VeroE6 cells were seeded at 1×10^6^ cells per well in 6-well plates. Cells were infected with MOI of 0.02, with VSV-SARS-CoV-2 Wuhan-Hu-1 D614G and four escape mutants mixed at equal frequencies. Following 1 h incubation, cell monolayers were washed three times with HBBS and cultures were incubated for 72 h in humidified incubators at 34°C. To passage the progeny viruses, virus mixture was continuously passaged four times in VeroE6 cells at MOI of 0.02. Cellular RNA samples from each passage were extracted using RNeasy Mini kit (QIAGEN) and subjected to next-generation sequencing as described previously to confirm the introduction and frequency of substitutions (*50*).

**Measurement of Fc-effector functions**

S2X324-dependent activation of human FcγRIIa and IIIa was performed with a bioluminescent reporter assay. CHO cells stably expressing full-length wild-type SARS-CoV-2 S (target cells) were incubated with different amounts of mAbs. After a 25-minute incubation, Jurkat cells stably expressing FcγRIIIa receptor (V158 variant) or FcγRIIa receptor (H131 variant) and NFAT-driven luciferase gene (effector cells) were added at an effector to target ratio of 6:1 for FcγRIIIa and 5:1 for FcγRIIa. Signaling was quantified by the luciferase signal produced as a result of NFAT pathway activation. Luminescence was measured after 22 hours of incubation at 37˚C with 5% CO_2_ with a luminometer using the Bio-Glo-TM Luciferase Assay Reagent according to the manufacturer’s instructions (Promega).

ADCC assays were performed using SARS-CoV2 CHO-K1 cells (genetically engineered to stably express a HaloTag-HiBit-tagged) as target cells and PBMC as effector cells at a 30:1 ratio. HiBit-cells were seeded at 3,000 cells/well and incubated for 16 hours at 37°C, while PBMCs isolated from fresh blood were cultivated overnight at 37°C 5% CO2 in the presence of 5 ng/ml of IL-2. The day after, media was removed from the target cells and titrated concentrations of mAbs were added before the addition of PBMCs at 100,000 cells/well. Digitonin at 100 µg/ml was used as 100% specific lysis control. After 4 hours of incubation at 37°C, ADCC was measured with Nano-Glo HiBiT Extracellular Detection System (Promega; Cat. Nr.: N2421) using a luminometer (Integration Time 00:30). The assays were performed on 3 different donors and AUC (Area Under the Curves) were calculated.

ADCP assay was performed using CHO cells stably expressing full-length wild-type SARS-CoV-2 S (target cells) and labelled with PKH67 (Sigma Aldrich) as targets. PMBCs from healthy donor were labelled with CellTrace Violet (Invitrogen) and used as source of phagocytic effector cells. Target cells (10,000/well) were incubated with titrated concentrations of mAbs for 10 min and then mixed with PBMCs (200,000/well). The next day, cells were stained with APC-labelled anti-CD14 mAb (BD Pharmingen), BV605-labelled anti-CD16 mAb (Biolegend), BV711-labelled anti-CD19 mAb (Biolegend), PerCP/Cy5.5-labelled anti-CD3 mAb (Biolegend), APC/Cy7-labelled anti-CD56 mAb (Biolegend) for the identification of CD14+ monocytes. After 20 minutes, cells were washed and fixed with 4% paraformaldehyde before acquisition on a ZE5 Cell Analyzer (Biorad). Data were analyzed using FlowJo software. The % ADCP was calculated as % of monocytes (CD3- CD19- CD14+ cells) positive for PKH67. The assays were performed on 2 different donors and AUC (Area Under the Curves) were calculated.

**Hamster challenge experiment**

Animal experiments were carried out by two independent laboratories. For the results shown in Fig.4A-C, 64 male golden Syrian hamsters (*Mesocricetus auratus;* RjHan:AURA) of 5-6 weeks of age (average weight 60-80 grams) were purchased from Janvier Laboratories (Le Genest-Saint-Isle, France) and handled under specific pathogen-free conditions. The animals were housed and manipulated in isolators in a Biosafety level-3 facility, with *ad libitum* access to water and food. Before manipulation, animals underwent an acclimation period of one week. Twenty-four hours before infection, the hamsters received an intraperitoneal injection of different concentrations of the ‘hamsterized’ monoclonal antibodies (mAb) S309 (0.6, 1.7, 5 and 15 mg/kg), S2X324 (0.2, 0.6, 1.7 and 5 mg/kg) or the control isotype MGH2 (15 mg/kg). ‘Hamsterization’ of human antibodies was based on the use of human VH and VL regions fused with the heavy and light chain constant regions of *Mesocricetus auratus IgG2a:*

CH:ATTTAPSVYPLAPGGTPDSTTVTLGCLVKGYFPEPVTVSWNSGALTSGVHTFPSVLHSGLYSLSSSVTVPSSTWPSQTVTCNVAHPASSTKVDKKIEPRSCTSLPTLCPKCPAPDLLGGPSVFIFPPNPKDVLTISLTPKVTCVVVDVSEDEPDVQFNWFVNNVEVKTAETQPRQQQFNSTYRVVSSLPIQHQDWLSSKEFKCKVNNKALPSPIEKTISKPRGQARIPQVYTLPPPTEQMTQKVVSLTCMITGFFPADVHVEWEKNGQPEQNYKNTSPVLDTDGSYFMYSKLNVPKSSWEQGNIYVCSVLHEALRNHHTTKAISRSLGN

CK:RSDAKPTVSIFPPSSEQLQSGSASLVCFVNNFYPKDINVKWKVDGSEKRDGVLQSITDQDSKDSTYSLSSTLTLTKGDYDSHNLYACEVTHKTSSTPIVKSLNKNEC

CL:GQPSAAPSVTLFPPSSEELKTNQATLVCMIKEFYPSDVKVTWESDGIPITQGVKTTQPSKRDNKYLATSFLTMTAEAWKSRNSISCQVTHGGTTVEKSLSPAACF

Animal infection was performed as previously described (*111*). Briefly, the animals were anesthetized with an intraperitoneal injection of 200 mg/kg ketamine (Imalgène 1000, Merial) and 10 mg/kg xylazine (Rompun, Bayer), and 100 µL of physiological solution containing 6x10^4^ PFU of SARS-CoV-2/VoC delta (GISAID ID : EPI_ISL_2029113, supplied by Dr Olivier Schwartz, Institut Pasteur, Paris, France) was then administered intranasally to each animal (50 µL/nostril). Mock-infected animals received the physiological solution only. Infected and mock-infected hamsters were housed in separated isolators and were followed-up daily, for four days, when the body weight and the clinical score were noted. At day 4 post-inoculation, the animals were euthanized with an excess of anesthetics (ketamine and xylazine) and exsanguination. Blood samples were collected by cardiac puncture; after coagulation, the tubes were centrifuged at 2,000 x g during 10 min at 4°C, the serum was collected and frozen at -80°C until further analyses. The lungs were collected, weighted and frozen at -80°C until further analyses.

For the results shown in Fig.4D-I, the hamster infection model of SARS-CoV-2 including the associated analytical procedures, have been described before (*112*, *113*). Briefly, female Syrian hamsters (Mesocricetus auratus) of 6-8 weeks old were anesthetized with ketamine/xylazine/atropine and inoculated intranasally with 50-100 μL containing 1×10^4^ TCID_50_ Delta (B.1.617.2; EPI_ISL_2425097), BA.2, or BA.5 (*114*). Animals were treated once by intraperitoneal injection 24h before or after SARS-CoV-2 challenge (i.e., prophylactic vs therapeutic administration) with ‘hamsterized’ or human S2X324 mAb. Hamsters were monitored for appearance, behavior and weight. At day 4 pi, hamsters were euthanized by i.p. injection of 500 μL Dolethal (200 mg/ml sodium pentobarbital, Vétoquinol SA). Lungs (and trachea for BA.2-infected animals) were collected for viral RNA and infectious virus quantification by RT-qPCR and end-point virus titration, respectively. Serum samples were collected at day 4 pi for analysis of Ab levels. Animals with circulating Ab levels below the detection limit, indicating misdosing, were excluded.

**SARS-CoV-2 RT-qPCR**

For data shown in Fig.4C, frozen lungs fragments were weighted and homogenized with 1 mL of ice-cold DMEM (31966021, Gibco) supplemented with 1% penicillin/streptomycin (15140148, Thermo Fisher) in Lysing Matrix M 2 mL tubes (116923050-CF, MP Biomedicals) using the FastPrep-24™ system (MP Biomedicals), and the following scheme: homogenization at 4.0 m/s during 20 sec, incubation at 4°C during 2 min, and new homogenization at 4.0 m/s during 20 sec. The tubes were centrifuged at 10.000 x g during 1 min at 4°C. Afterwards, 125 µL of the tissue homogenate supernatant were mixed with 375 µL of Trizol LS (10296028, Invitrogen) and the total RNA was extracted using the Direct-zol RNA MiniPrep Kit (R2052, Zymo Research). The presence of SARS-CoV-2 RNA in these samples was evaluated by one-step RT-qPCR in a final volume of 12.5 μL per reaction in 384-wells PCR plates using a thermocycler (QuantStudio 6 Flex, Applied Biosystems). Briefly, 2.5 μL of RNA were added to 10 μL of a master mix containing 6.25 μL of 2X reaction mix, 0.2 µL of MgSO_4_ (50 mM), 0.5 µL of Superscript III RT/Platinum Taq Mix (2 UI/µL) and 3.05 μL of nuclease-free water containing the nCoV_IP2 primers (nCoV_IP2-12669Fw: 5’-ATGAGCTTAGTCCTGTTG-3'; nCoV_IP2-12759Rv: 5’-CTCCCTTTGTTGTGTTGT-3’) at a final concentration of 400 nM, and the nCoV_IP2 probe (5’-FAM-AGATGTCTTGTGCTGCCGGTA-3'-TAMRA) at a final concentration of 200 nM (*115*).The amplification conditions were as follows: 55°C for 20 min, 95°C for 3 min, 50 cycles of 95°C for 15 s and 58°C for 30 s, and a last step of 40°C for 30 s. Viral load quantification (expressed as RNA copy number/g of tissue) was assessed by linear regression using a standard curve of six known quantities of RNA transcripts containing the *RdRp* sequence (ranging from 10^7^ to 10^2^ copies).

For data shown in Fig. 4D, 4I and 4F, hamster lung and trachea tissues were collected after sacrifice and were homogenized using bead disruption (Precellys) in 350 µL TRK lysis buffer (E.Z.N.A.® Total RNA Kit, Omega Bio-tek) and centrifuged (10.000 rpm, 5 min) to pellet the cell debris. RNA was extracted according to the manufacturer’s instructions. RT-qPCR was performed on a LightCycler96 platform (Roche) using the iTaq Universal Probes One-Step RT-qPCR kit (BioRad) with N2 primers and probes targeting the nucleocapsid (*112*). Standards of SARS-CoV-2 cDNA (IDT) were used to express viral genome copies per mg tissue.

**End-point virus titrations**

For data shown in Fig. 4A, frozen lung fragments were weighted and homogenized with 1 mL of ice-cold DMEM supplemented with 1% penicillin/streptomycin (15140148, Thermo Fisher) in Lysing Matrix M 2 mL tubes (116923050-CF, MP Biomedicals) using the FastPrep-24™ system (MP Biomedicals), and the following scheme: homogenization at 4.0 m/s during 20 sec, incubation at 4°C during 2 min, and new homogenization at 4.0 m/s during 20 sec. The tubes were then centrifuged at 10.000 x g during 2 min at 4°C, and the supernatants were collected. Viral titers were obtained by classical TCID_50_ method on VeroE6 cells over 72 hours (*116*). For data shown in Fig. 4E and 4H, lung tissues were homogenized using bead disruption (Precellys) in 350 µL minimal essential medium and centrifuged (10,000 rpm, 5min, 4°C) to pellet the cell debris. To quantify infectious SARS-CoV-2 particles, endpoint titrations were performed on confluent Vero E6 or A549-Dual^TM^ hACE2-TMPRSS2 cells in 96- well plates for Delta, and BA.5, respectively. Viral titers were calculated by the Reed and Muench method (*117*) using the Lindenbach calculator and were expressed as 50% tissue culture infectious dose (TCID_50_) per mg tissue.

**Molecular Dynamics (MD) simulations**

Two structures of the BA.2 RBD and one of the Wuhan-Hu-1 RBD were prepared as previously described (*118*, *119*). The coordinates of the first RBD (based on the BA.1 structure) were obtained from PDB 7TN0 (*18*) for which the glycan at position 343 was extended as previously described (*27*) using ISOLDE (*120*) to visually place, link and minimize each monosaccharide beyond the *N*-acetylglucosamine which was modeled in the X-ray structure. Positions 371, 376, 405, and 408 were mutated to the corresponding BA.2 residues, followed by optimization of rotamers at these positions using Repack in Protein Builder (MOE v2020.0901). The resulting model was then prepared using QuickPrep (MOE v2020.0901, <https://www.chemcomp.com>). The coordinates of the second BA.2 RBD (based on the experimental structure of BA.2) were obtained from PDB 7UB0 (*45*) with the glycan at position 343 from the above structure superposed via the N343 residue and GlcNAc, which were locally minimized, and the model prepared using QuickPrep. The coordinates of the Wuhan-Hu-1 RBD were obtained from PDB 6M0J (*94*) for which ACE2 was removed and the glycan at position 343 added using ISOLDE (*120*) as previously described (*27*).

The RBD structures were each parameterized using tleap with the Amber ff14SB force field (*121*) for the protein, GLYCAM_06j-1 for the glycan (*122*), TIP3P for water (truncated octahedral cell with 18 Å buffer around solute) (*123*); the Joung & Cheatham parameters (*124*) were used for the ions neutralizing the charged solute and the additional 0.15 M excess Na+ and Cl- ions.

We generated 4.8 μs of aggregate molecular dynamics for each of the 3 RBDs by performing eight independent simulations seeded with different initial velocities. Each of these 24 simulations was subjected to a nine-stage minimization and equilibration procedure as previously described (*118*). This involved 10,000 steps of restrained minimization with a 100 kcal/mol Å^2^ force constant applied to all heavy atoms resolved in the structure followed by 100 ps of restrained MD heating to 300 K, 100 ps of restrained MD at 300 K, 250 ps of restrained MD with a 10-fold reduction in restraint force constant at 300 K, 10,000 steps of backbone-restrained minimization, 100 ps of backbone-restrained MD, 100 ps of backbone-restrained MD at 300 K, 100 ps of backbone-restrained MD with a further 10-fold reduction in restraint force constant, 100 ps of backbone-restrained MD with another 10-fold reduction in restraint force constant (0.1 kcal/mol Å^2^), and 2.5 ns of unrestrained MD at 300 K. An additional 0.6 μs of unrestrained MD simulation was performed for each of the 24 independent simulations.

Simulations were processed using cpptraj (*125*) to image the coordinates, RMS-align RBD C⍺ atoms to the S309:RBD complex (PDB 7TN0 (*18*)), and to strip solvent and ions. Volumetric density maps were computed using cpptraj with grid points spaced 0.25 Å over a grid with dimensions 279 Å, 296 Å, 307 Å. This resulted in Cartesian coordinates of grid points and the probability of finding any glycan atom at those grid points. This procedure was repeated for S309 from the S309:RBD complex used to align RBDs above. The maximum probability (isovalue) at which no glycan atom from RBD Wuhan-Hu-1 occupied a grid point also occupied by S309 was 0.057 and this isovalue was used for subsequent analysis with the two BA.2 RBDs. Overlap was calculated by counting the grid points occupied by S309 (probabilities were one or zero because it was a static structure) and any glycan atom if the probability for the glycan was 0.057 or less at that grid point. Smaller isovalues increase the volume size because ephemeral glycan conformations are included in the map whereas a larger isovalue (occupancy probability) reduces the volume size because only persistent glycan conformations are included in the map. The total volume housed by a volumetric density map for a glycan was computed by counting the grid points occupied by the glycan with an isovalue of 0.057. Renderings for volumetric density maps of the glycan were generated using VMD (*126*).


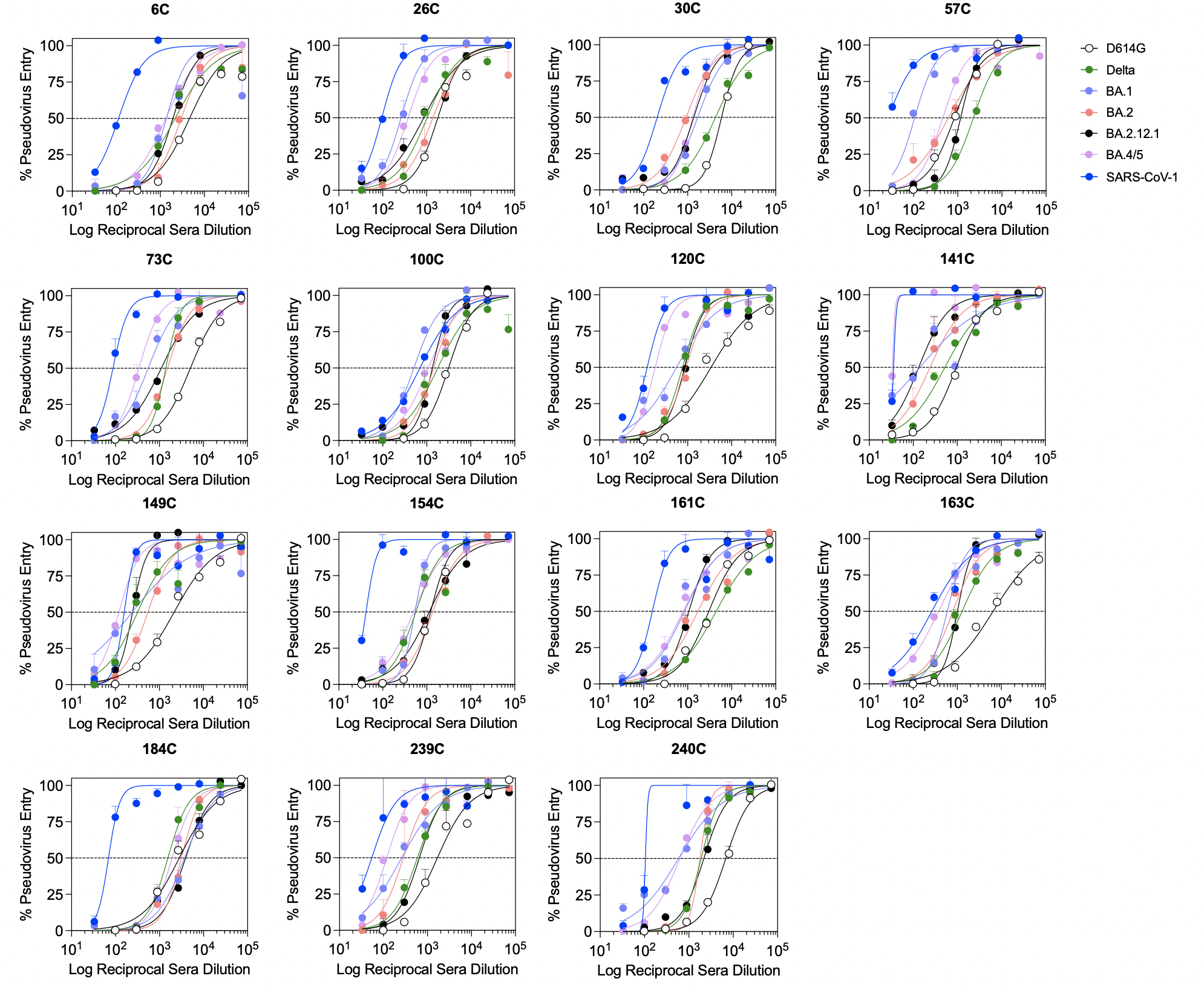


**Fig. S1A**. Normalized dose-response neutralization curves using plasma from individuals previously infected in 2020 (with a Washington-1-like SARS-CoV-2 strain) and then vaccinated twice (‘Infected-vaccinated 2 doses’) using VSV pseudovirus harboring the Wu-G614 S (white circles), Delta S (green circles), Omicron BA.1 S (light blue circles), Omicron BA.2 S (orange circles), Omicron BA.2.12.1 S (black circles), Omicron BA.4/5 S (pink circles) and SARS-CoV S (dark blue circles). One representative experiment out of at least two biological replicates is shown.


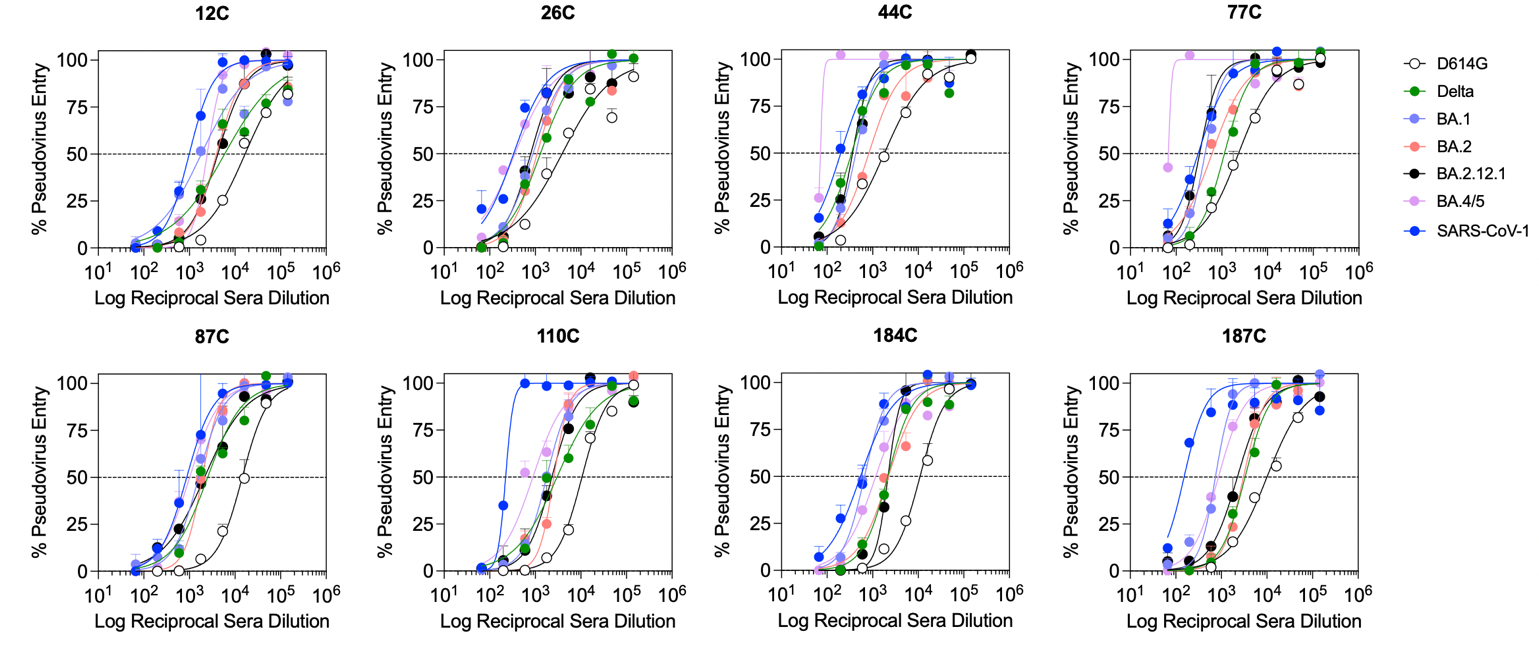


**Fig. S1B**. Normalized dose-response neutralization curves using plasma from individuals previously infected in 2020 (with a Washington-1-like SARS-CoV-2 strain) and then vaccinated three times (‘Infected-vaccinated 3 doses’) using VSV pseudovirus harboring the Wu-G614 S (white circles), Delta S (green circles), Omicron BA.1 S (light blue circles), Omicron BA.2 (orange circles), Omicron BA.2.12.1 S (black circles), Omicron BA.4/5 S (pink circles) and SARS-CoV S (dark blue circles). One representative experiment out of at least two biological replicates is shown.


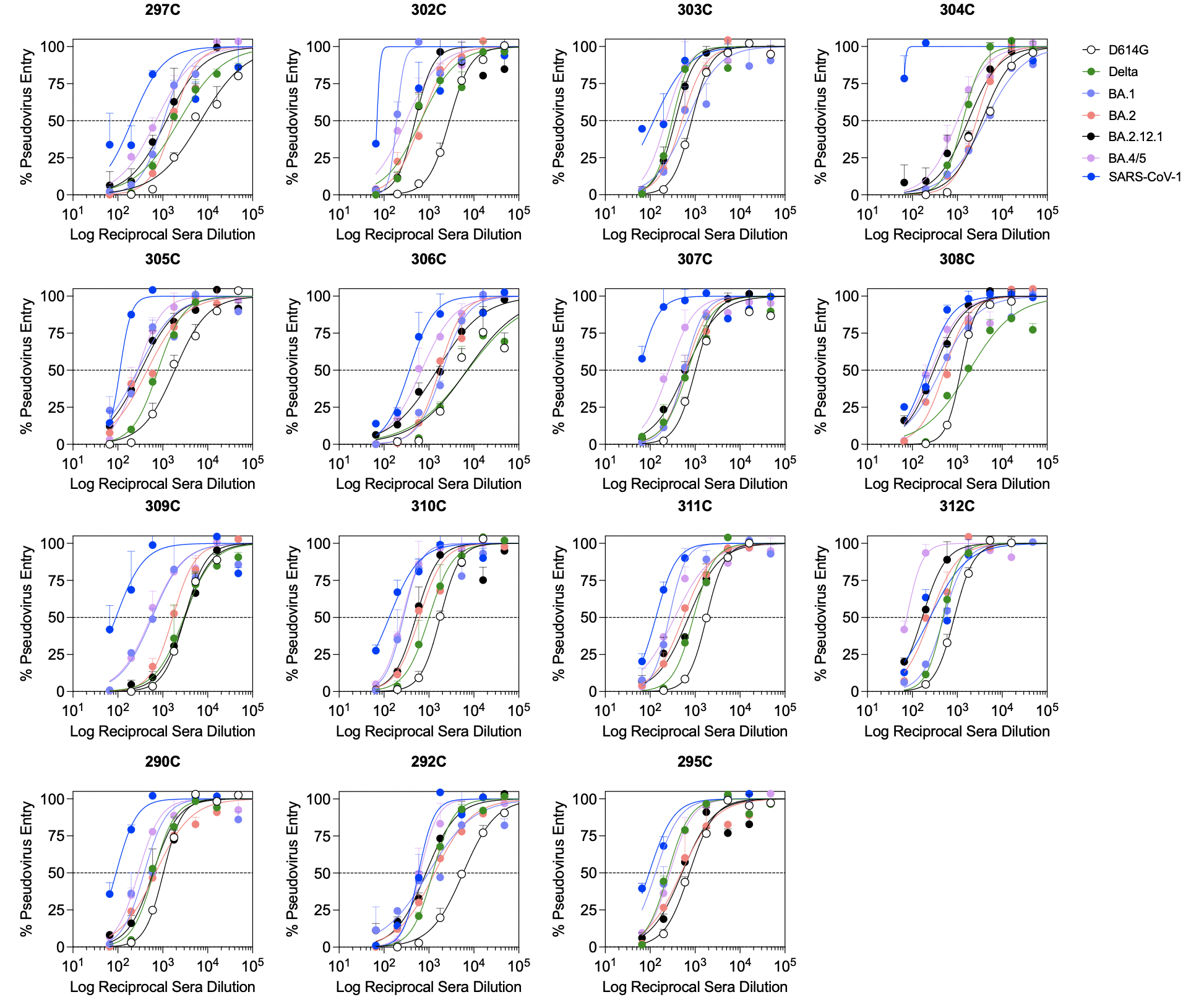


**Fig. S1C**. Normalized dose-response neutralization curves using plasma from Delta Breakthrough cases (‘Delta breakthrough 3 doses’) using VSV pseudovirus harboring the Wu-G614 S (white circles), Delta S (green circles), Omicron BA.1 S (light blue circles), Omicron BA.2 (orange circles), Omicron BA.2.12.1 S (black circles), Omicron BA.4/5 S (pink circles) and SARS-CoV S (dark blue circles). One representative experiment out of at least two biological replicates is shown.


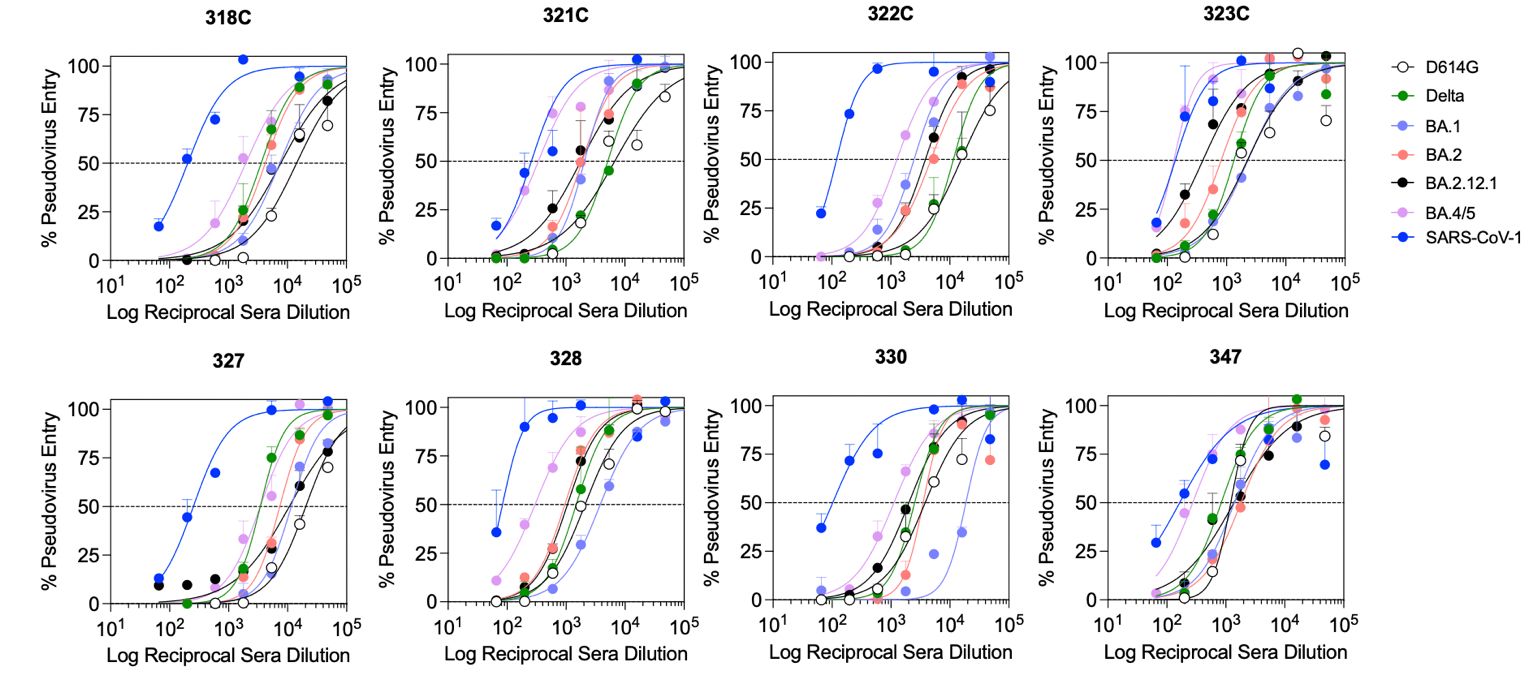


**Fig. S1D**. Normalized dose-response neutralization curves using plasma from BA.1 breakthrough cases ‘(BA.1 breakthrough 2 doses’) using VSV pseudovirus harboring the Wu-G614 S (white circles), Delta S (green circles), Omicron BA.1 S (light blue circles), Omicron BA.2 (orange circles), Omicron BA.2.12.1 S (black circles), Omicron BA.4/5 S (pink circles) and SARS-CoV S (dark blue circles). One representative experiment out of at least two biological replicates is shown.


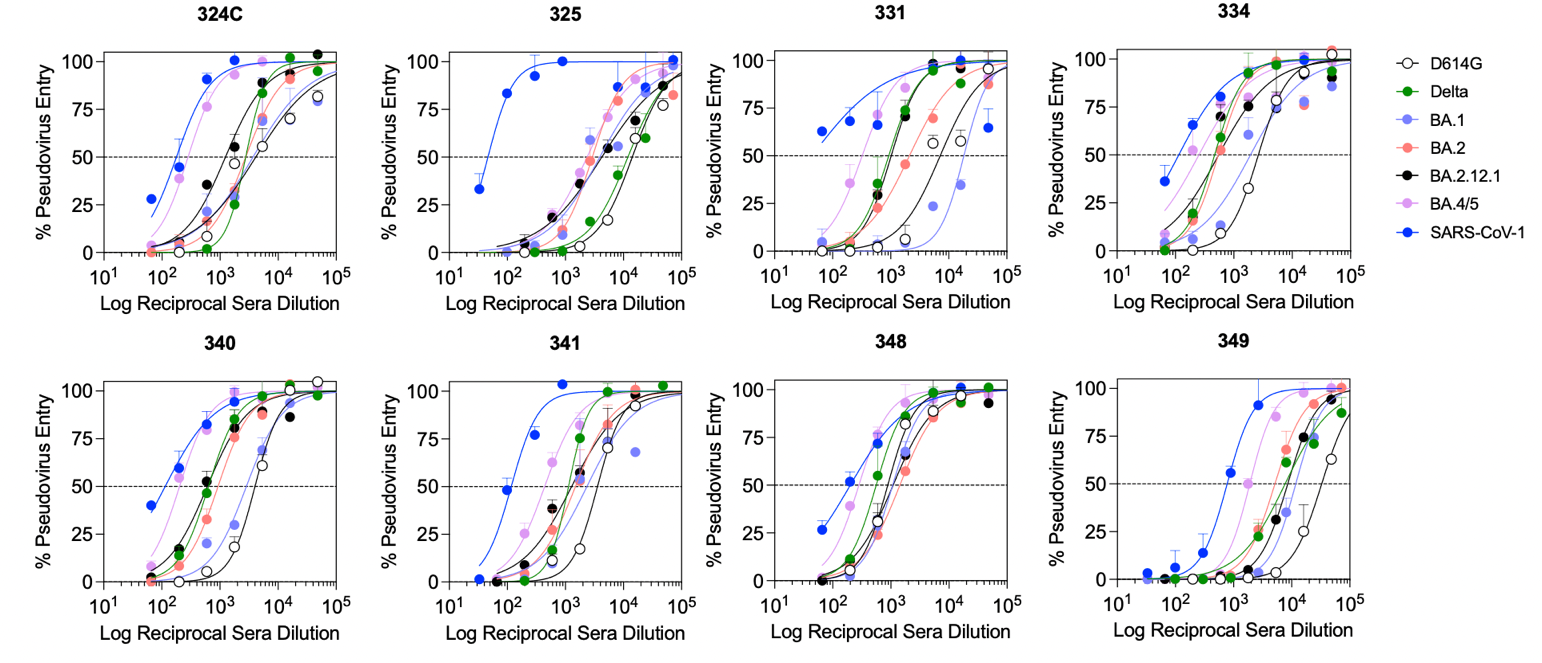


**Fig. S1E**. Normalized dose-response neutralization curves using plasma from BA.1 breakthrough cases ‘(BA.1 breakthrough 3 doses’) using VSV pseudovirus harboring the Wu-G614 S (white circles), Delta S (green circles), Omicron BA.1 S (light blue circles), Omicron BA.2 (orange circles), Omicron BA.2.12.1 S (black circles), Omicron BA.4/5 S (pink circles) and SARS-CoV S (dark blue circles). One representative experiment out of at least two biological replicates is shown.


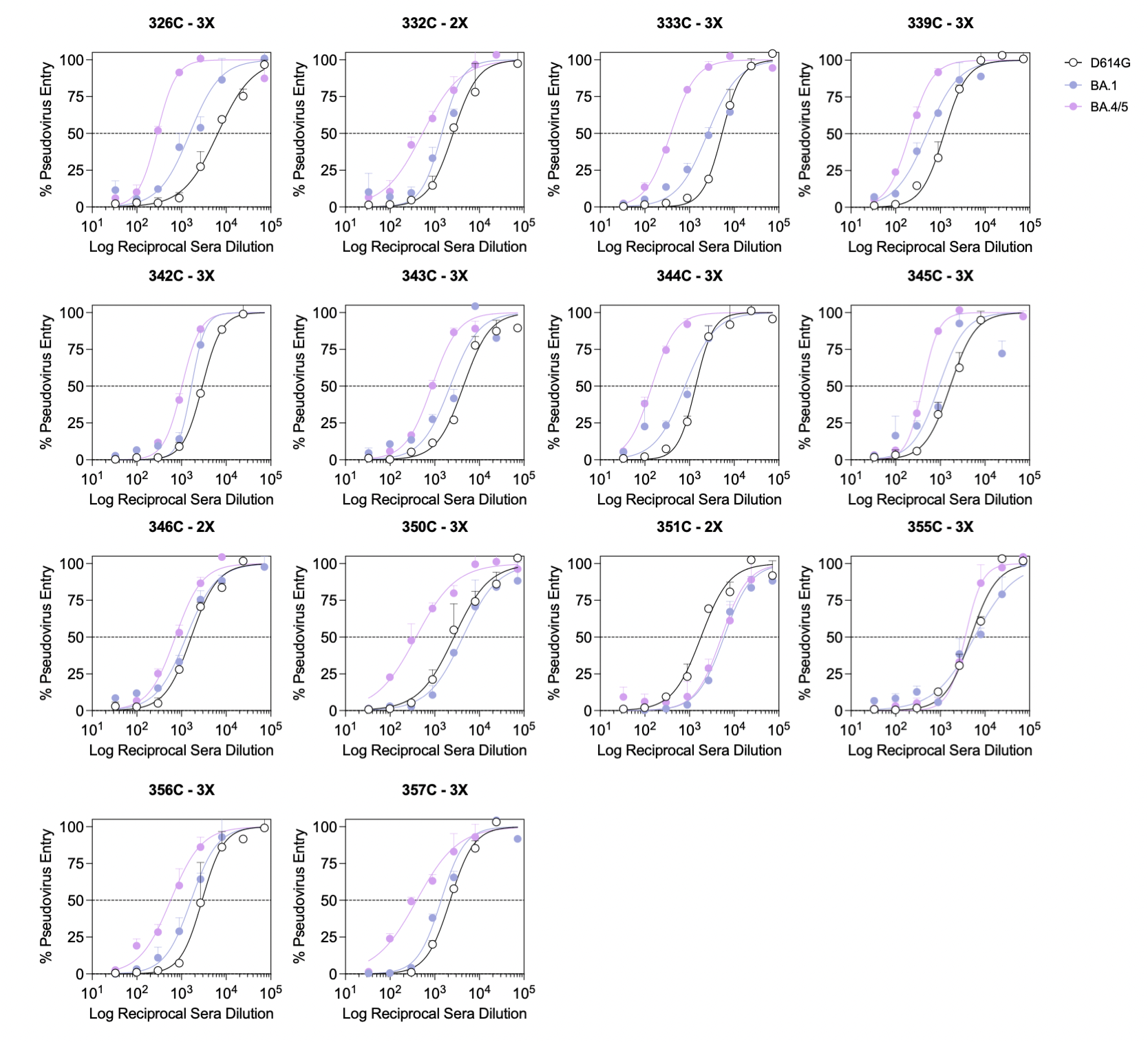


**Fig. S1F**. Normalized dose-response neutralization curves using plasma from BA.1 breakthrough cases from whom nasal swab samples were obtained. ‘(BA.1 breakthrough 2/3 doses’) using VSV pseudovirus harboring the Wu-G614 S (white circles), Omicron BA.1 S (light blue circles), and Omicron BA.4/5 S (pink circles). One representative experiment out of at least two biological replicates is shown.


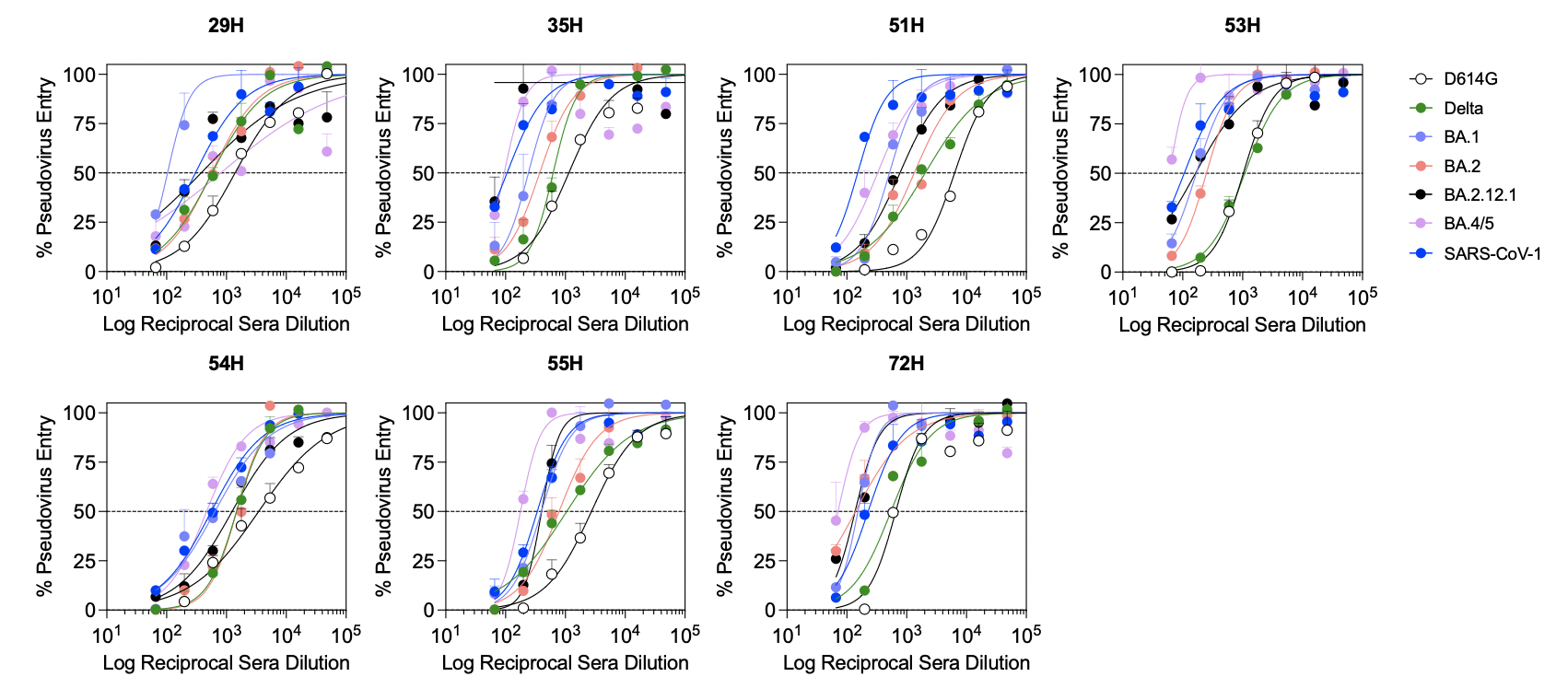


**Fig. S1G**. Normalized dose-response neutralization curves using plasma from vaccinated-only individuals (‘vaccinated-only 3 doses’) using VSV pseudovirus harboring the Wu-G614 S (white circles), Delta S (green circles), Omicron BA.1 S (light blue circles), Omicron BA.2 (orange circles), Omicron BA.2.12.1 S (black circles), Omicron BA.4/5 S (pink circles) and SARS-CoV S (dark blue circles). One representative experiment out of at least two biological replicates is shown.

**Fig. S2.** **Variant prevalence in Switzerland at the end of 2021 and early 2022**


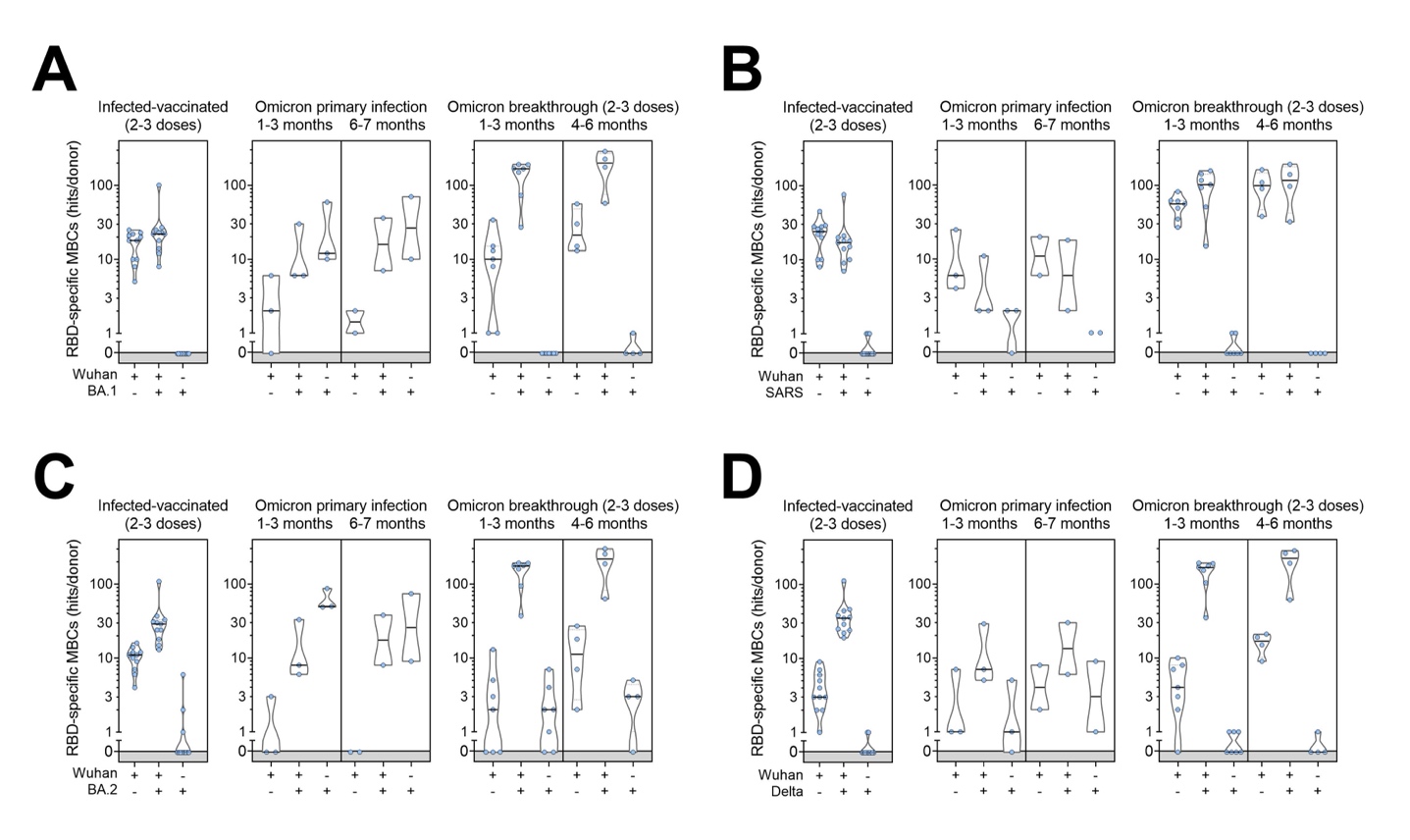


**Fig. S3.** Cross-reactivity with the Wuhan-Hu-1 and the BA.1 RBDs (A), the Wuhan-Hu-1 and SARS-CoV RBDs (B), the Wuhan-Hu-1 and BA.2 RBDs (C) or the Wuhan-Hu-1 and Delta RBDs (D) of IgGs secreted from memory B cells obtained from infected-vaccinated individuals (n=11), subjects who experienced a primary infection with Omicron (n=3 samples collected at 1-3 months and n=2 samples collected at 6-7 months post infection) or a breakthrough infection in January-March 2022 (n=7 samples collected at 1-3 months and n=4 samples collected at 4-6 months post infection). Individual dots correspond to number of positive hits as shown in Fig. 1B and fig. S4A-B relative to individual donors.
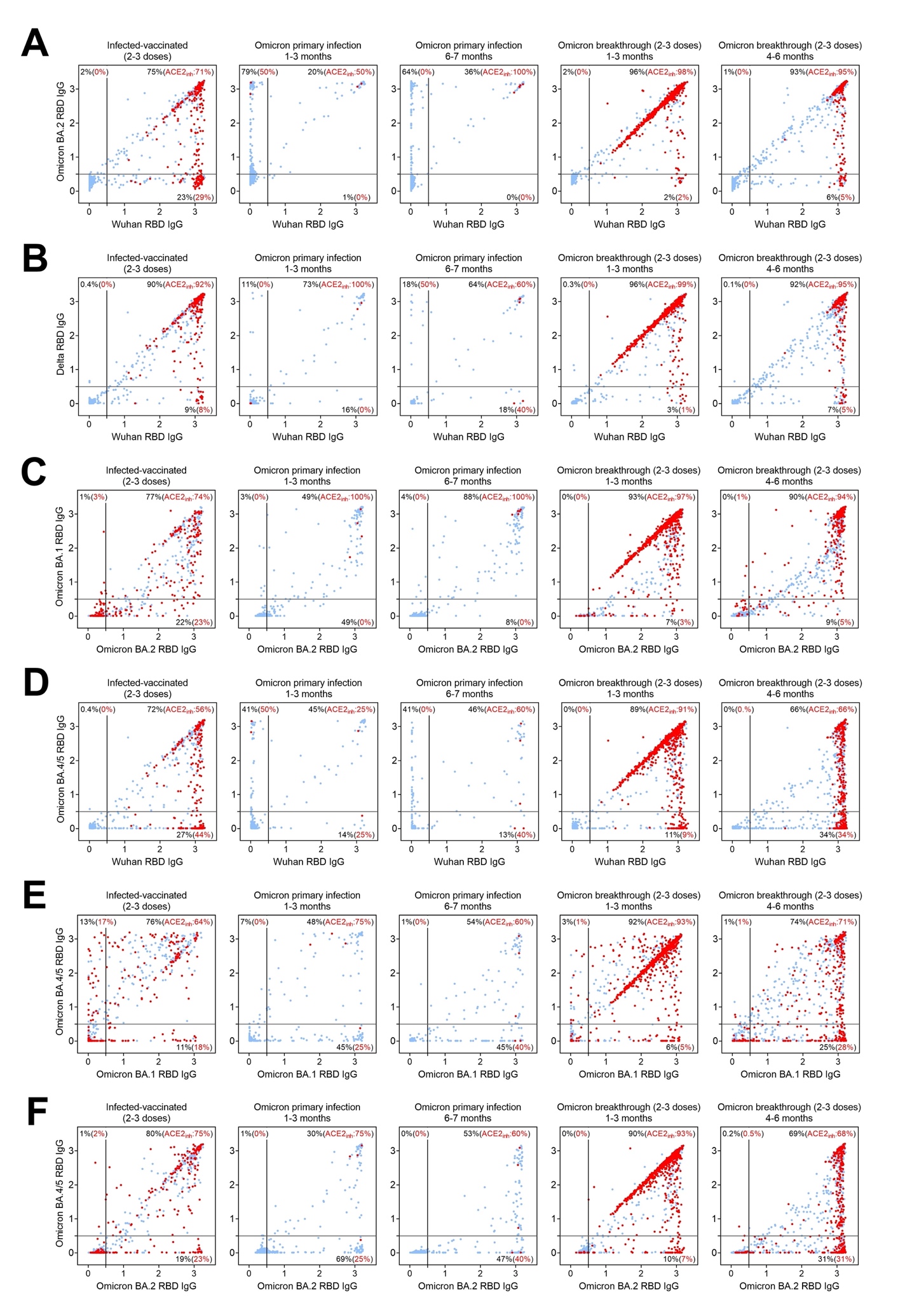


**Fig. S4.** **Cross-reactivity of IgG antibodies secreted by in vitro stimulated memory B cells.** **A-F**, Antigen-specific memory B cell repertoire analysis (AMBRA) on PBMCs obtained from infected-vaccinated individuals (n=11), primary SARS-CoV-2 infection (n=3 samples collected at 1-3 months and n=2 samples collected at 6-7 months) or breakthrough cases (n=7 samples collected at 1-3 months and n=4 samples collected at 4-6 months) occurring in January-March 2022 (Data S1). Each dot represents a well containing oligoclonal B cell supernatant screened for the presence of secreted IgGs binding to SARS-CoV-2 Wuhan-Hu-1 and BA.2 RBDs (A), Wuhan-Hu-1 and Delta RBDs (B), BA.2 and BA.1 RBDs (C), Wuhan-Hu-1 and BA.4/5 RBDs (D), BA.1 and BA.4/5 RBDs (E) or BA.2 and BA.4/5 RBDs (F) using ELISA. Red dots indicate inhibition of the interaction with ACE2 (using Wuhan-Hu-1 RBD target antigen) as determined in a separate assay (Fig. S6). Percentages are calculated relative to the total of positive hits against any of the antigen tested in a given figure panel.

**Fig. S5. Cross-reactivity of antibodies obtained from single-plasma cell cultures.** Circulating CD138+ cells isolated from 3 vaccinated donors (A to C) approximately 1 week after Omicron breakthrough infection and cultured at 0.5 cells/well for 3 days. Culture supernatants were tested by ELISA for the presence of IgG binding to the SARS-CoV-2 Wuhan-Hu-1, BA.1 and SARS-CoV RBDs. Each dot represents the optical density (OD) values of individual cultures for each antigen tested.


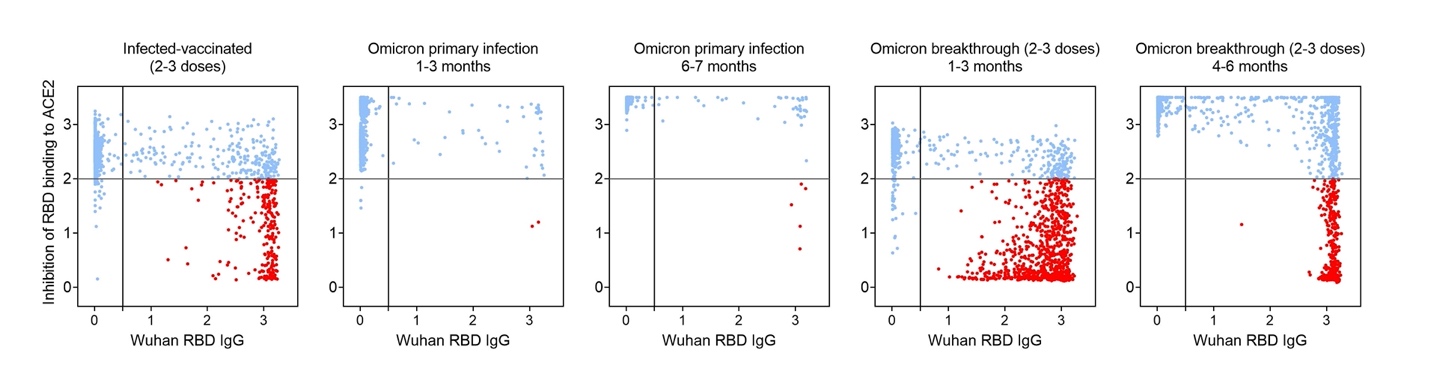


**Fig. S6. Inhibition of RBD binding to human ACE2 by memory B cell-derived secreted IgG antibodies.** Antibody-mediated inhibition of SARS-CoV-2 Wuhan-Hu-1 RBD binding to solid phase ACE2 in function of IgG binding to matched RBD, as determined by ELISA. Each dot represents the optical density (OD) values of individual cultures. Red: cultures with antibodies inhibiting ACE2 binding to the RBD (as defined by a y-axis cutoff of OD≤2)


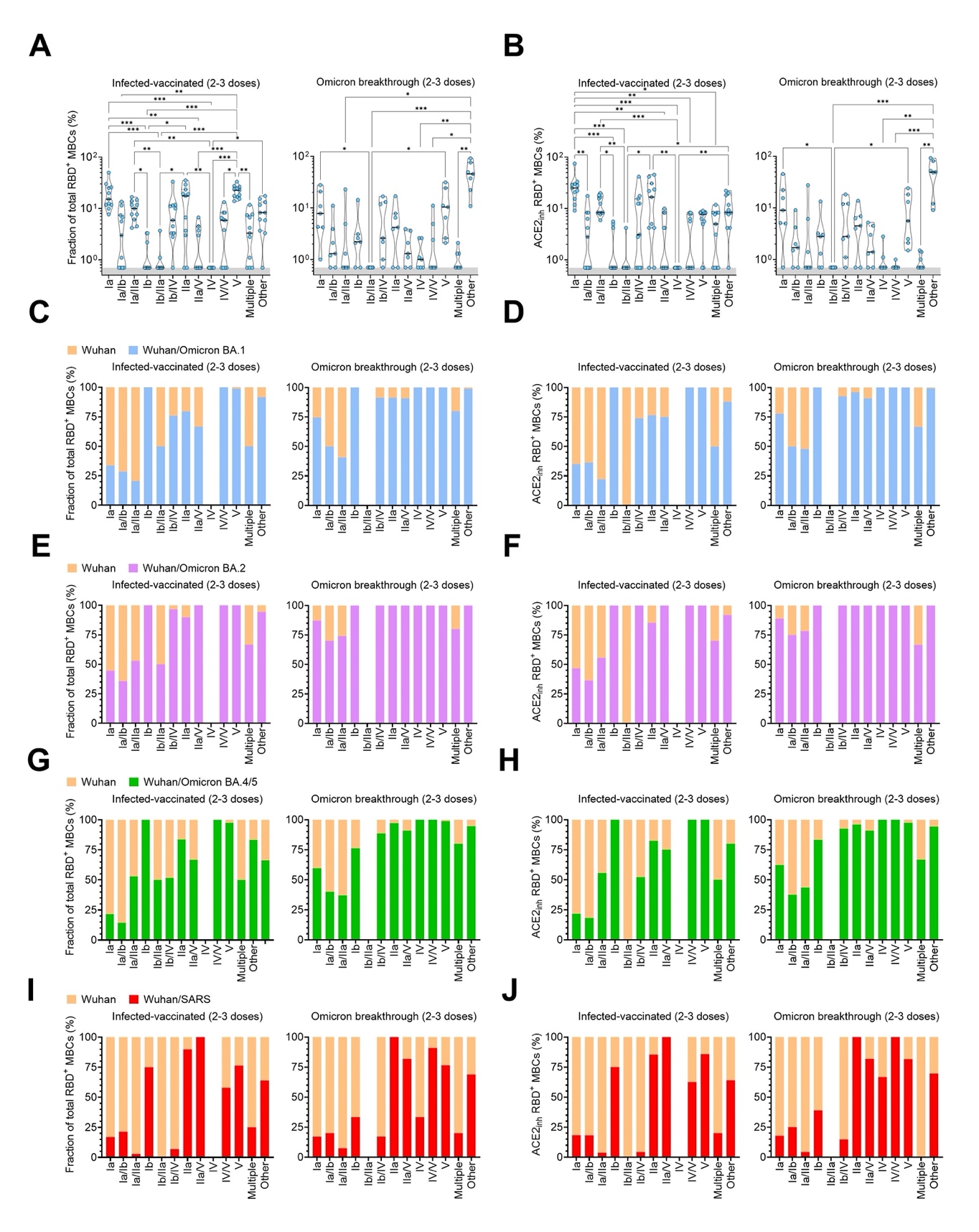


**Fig. S7.** **Epitope mapping and frequency analysis of RBD-directed IgG antibodies derived from memory B cells.** **A-J,** Frequency analysis of site-specific IgG antibodies derived from memory B cells, as defined by a blockade-of-binding assay using mAbs specific for sites Ia (S2E12), Ib (S2X324), IIa (S2X259), IV (S309; parent of sotrovimab) and V (S2H97). Hybrid sites Ia/Ib, Ia/IIa, Ib/IIa, Ib/IV, IIa/V and IV/V were defined by competition with the two corresponding mAbs. Hybrid sites exhibiting competition with more than 2 mAbs are indicated as “Multiple”. Lack of competition is indicated as “Other”. The graphs show the frequencies of total RBD-directed IgGs (A, C, E, G, I) and those inhibiting binding of RBD to human ACE2 (B, D, F, H, J). Violin plots (A, B) show percentages of site-specific IgGs in individual donors analyzed. Bars (C-J) show cumulative frequencies of site-specific IgG antibodies filtered for their binding to Wuhan-Hu-1 and Omicron BA.1 RBDs (C, D), Wuhan-Hu-1 and BA.2 RBDs (E, F), Wuhan-Hu-1 and BA.4/5 RBDs (G, H), and Wuhan-Hu-1 and SARS-CoV RBDs (I, J).


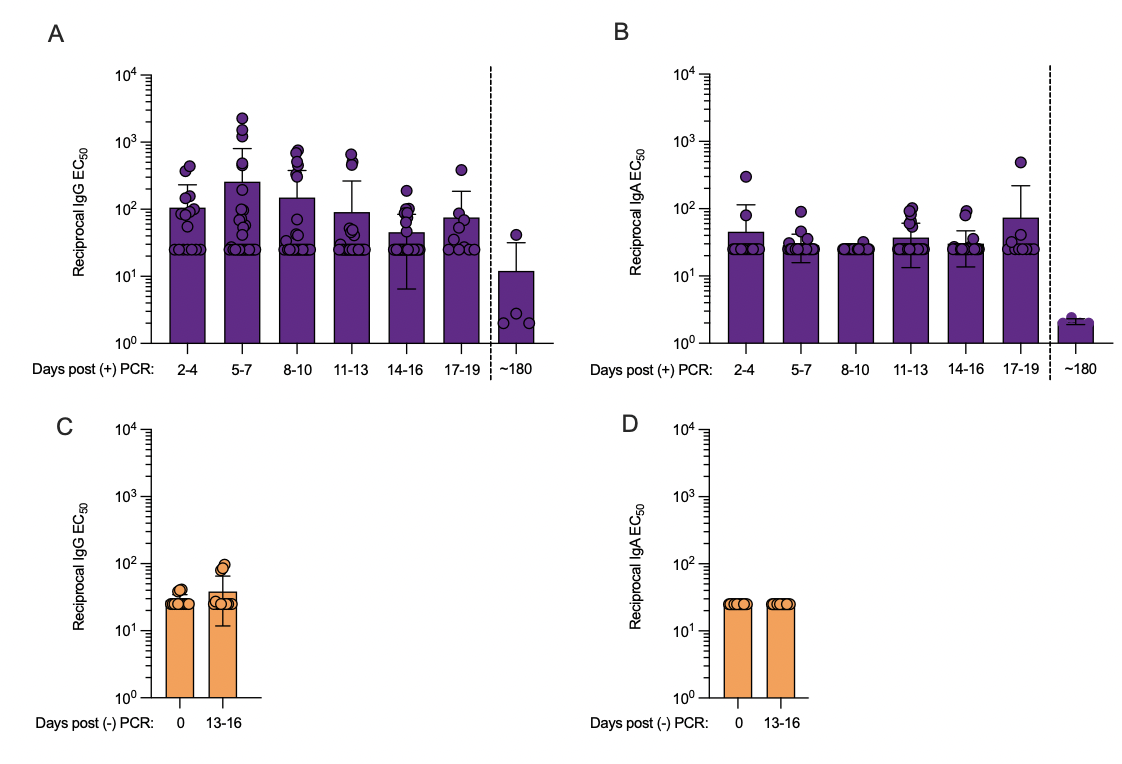


**Fig. S8.** IgG (A, C) or IgA (B,D) binding titers evaluated by ELISA using the prefusion SARS-CoV-2 S V-FLIP in nasal swabs obtained longitudinally upon BA.1 breakthrough infection following positive PCR (post (+) PCR, A-B) or in vaccinated-only individuals following a negative PCR test (post (-) PCR, C-D).


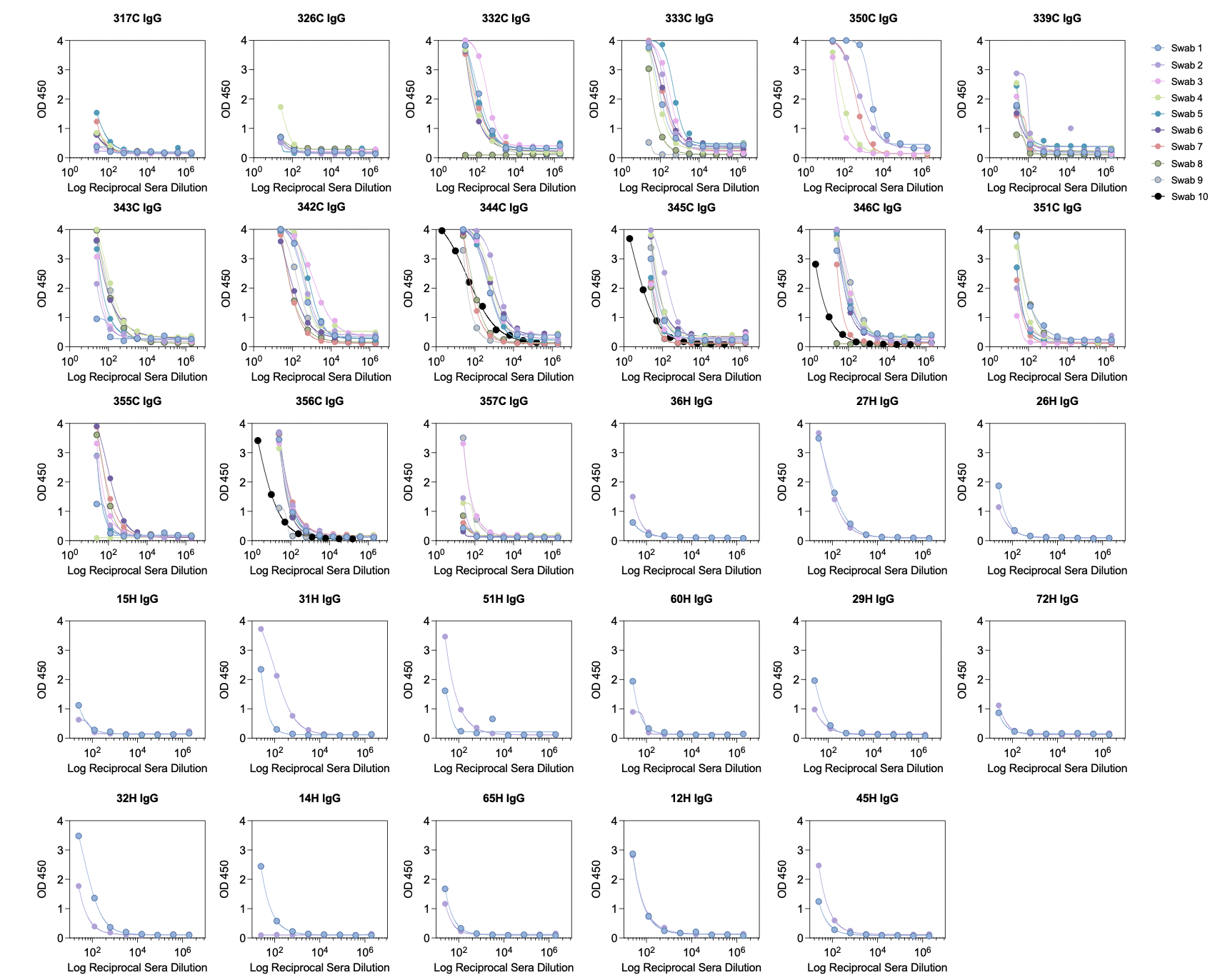


**Fig. S9A.** Dose-response curves of IgG binding to prefusion SARS-CoV-2 S VFLIP analyzed by ELISA using samples obtained from nasal swabs of BA.1 breakthrough cases (C) and vaccinated-only subjects (H). Each swab in the series is rendered with a distinct color and individual time points can be found in Data S1.


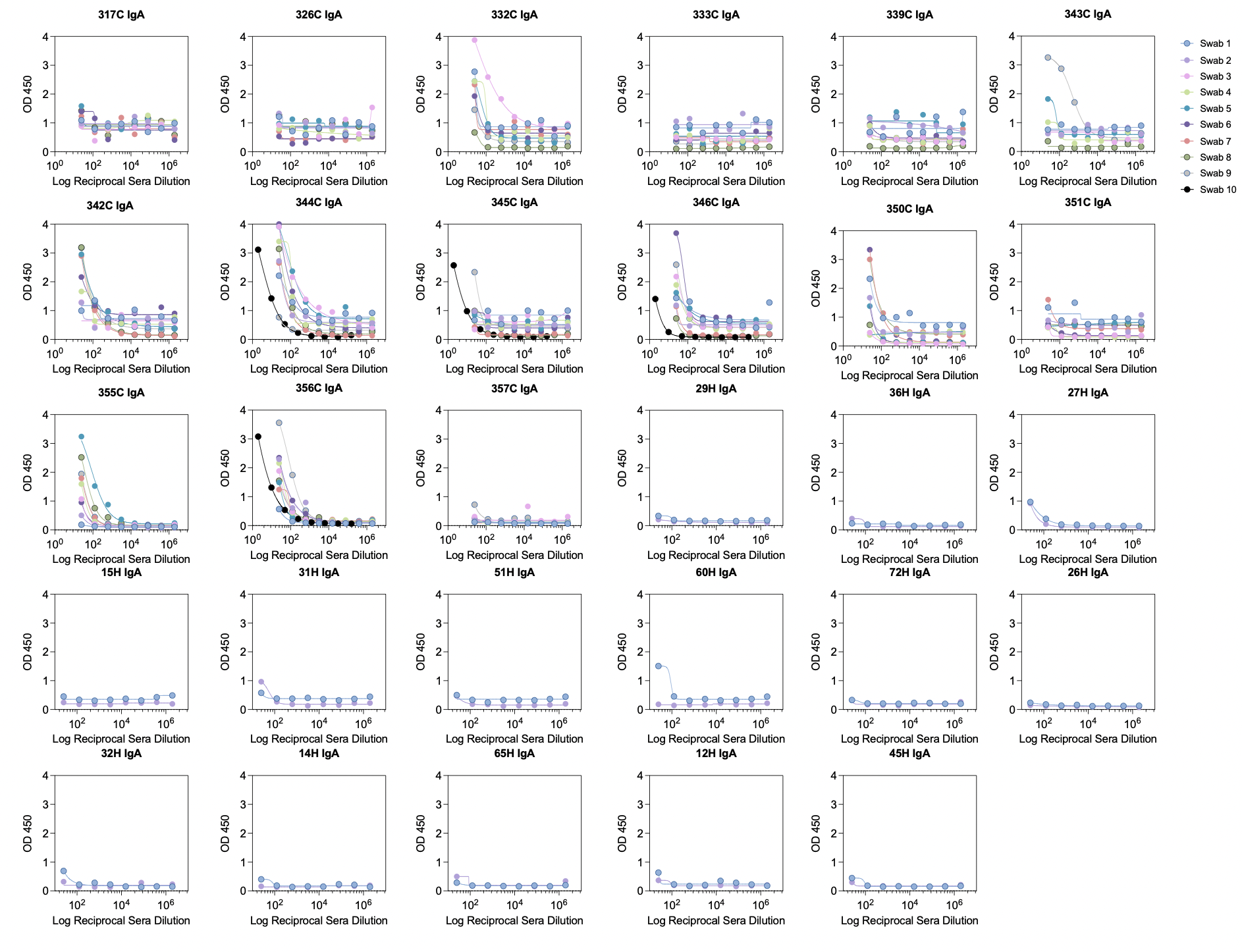


**Fig. S9B.** Dose-response curves of IgA binding to prefusion SARS-CoV-2 S VFLIP analyzed by ELISA using samples obtained from nasal swabs of BA.1 breakthrough cases (C) and vaccinated-only subjects (H). Each swab in the series is rendered with a distinct color and individual time points can be found in Data S1.


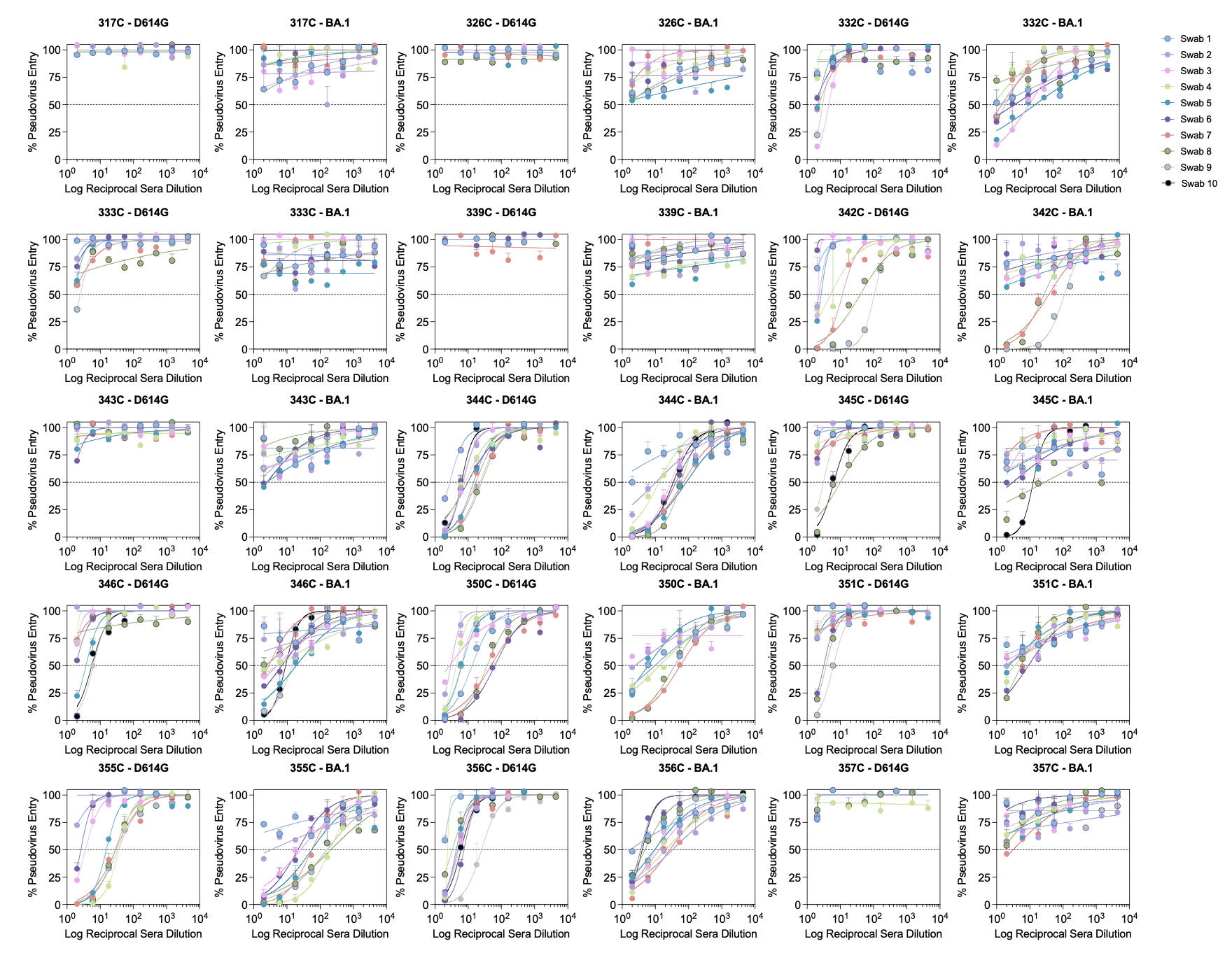


**Fig. S9C.** Normalized dose-response neutralization curves using nasal swab samples obtained from BA.1 breakthrough cases using VSV pseudovirus harboring Wu-G614 S or Omicron BA.1 S. Each swab in the series is rendered with a distinct color and individual time points can be found in Data S1. One representative experiment out of at least two biological replicates is shown.


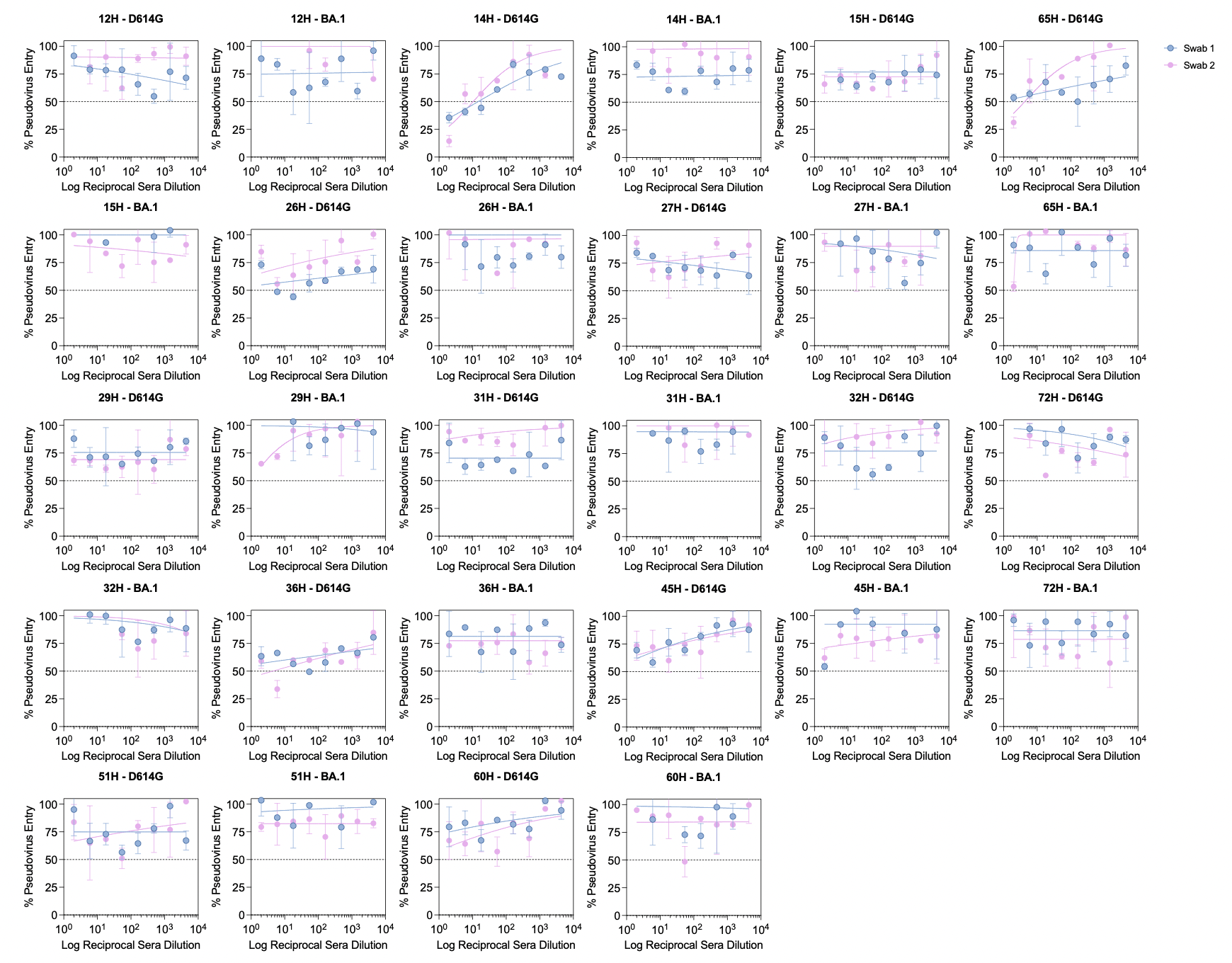


**Fig. S9D.** Normalized dose-response neutralization curves using nasal swab samples obtained from vaccinated-only individuals using VSV pseudovirus harboring Wu-G614 S or Omicron BA.1 S. Each swab in the series is rendered with a distinct color and individual time points can be found in Data S1. One representative experiment out of at least two biological replicates is shown.


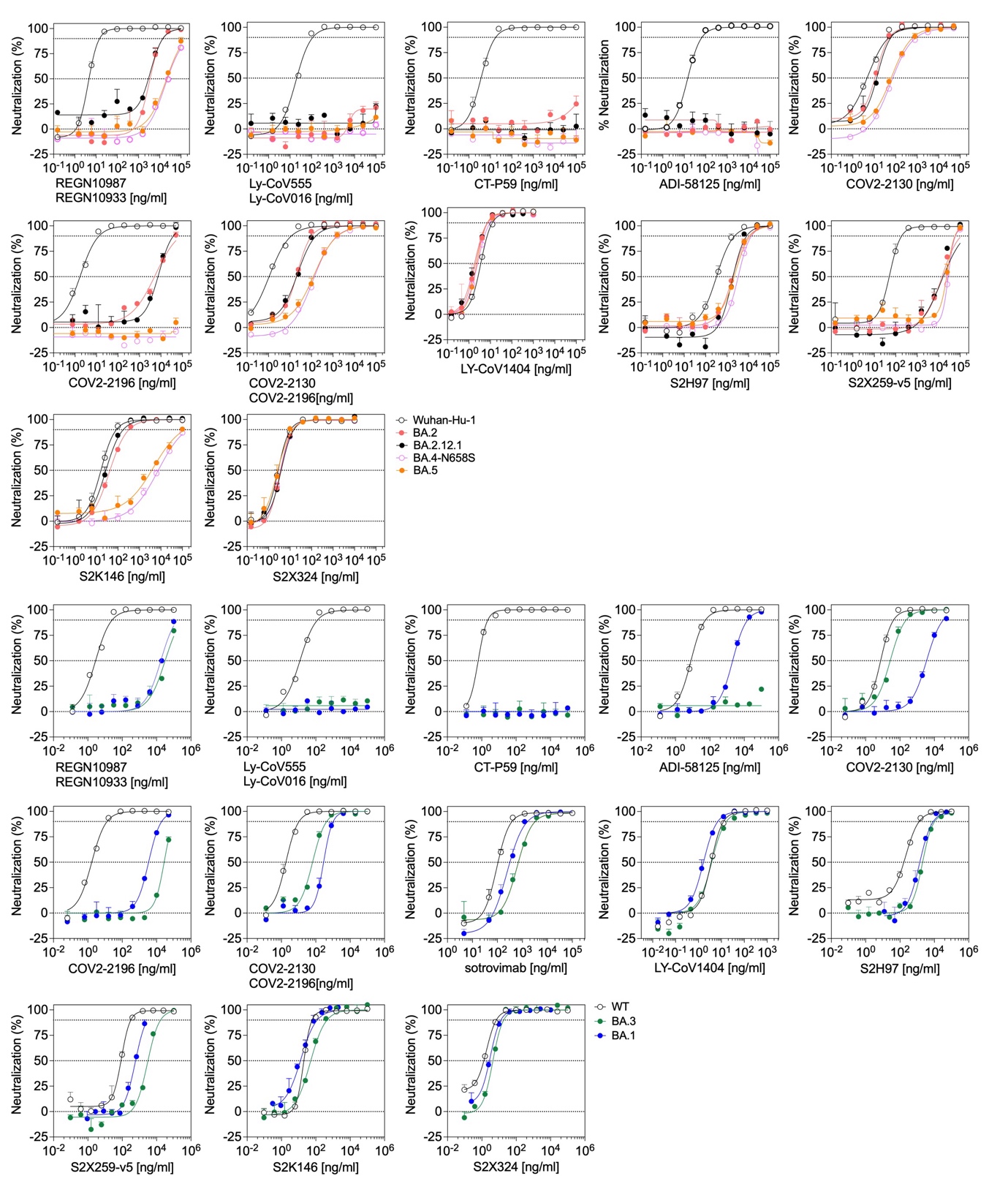


**Fig. S10A.** mAb-mediated neutralization of Wuhan-Hu-1, BA.1, BA.2, BA.3, BA.4-N658S, BA.5 and BA.2.12.1 S VSV pseudoviruses. One representative experiment out of at least two biological replicates is shown.

**
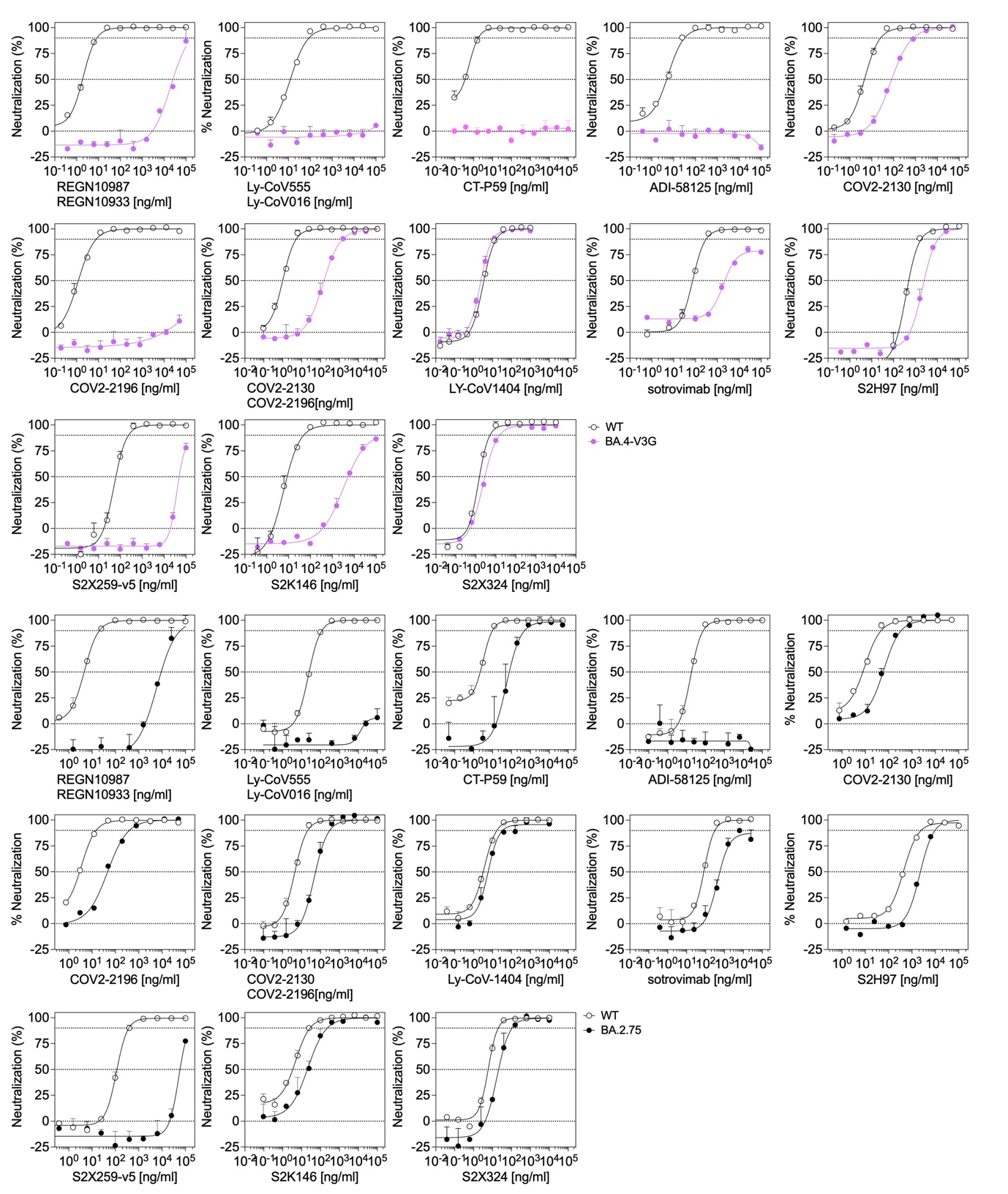
**

**Fig. S10B**: mAb-mediated neutralization of Wuhan-Hu-1, BA.4-V3G and BA.2.75 S VSV pseudoviruses. One representative experiment out of at least two biological replicates is shown.

**
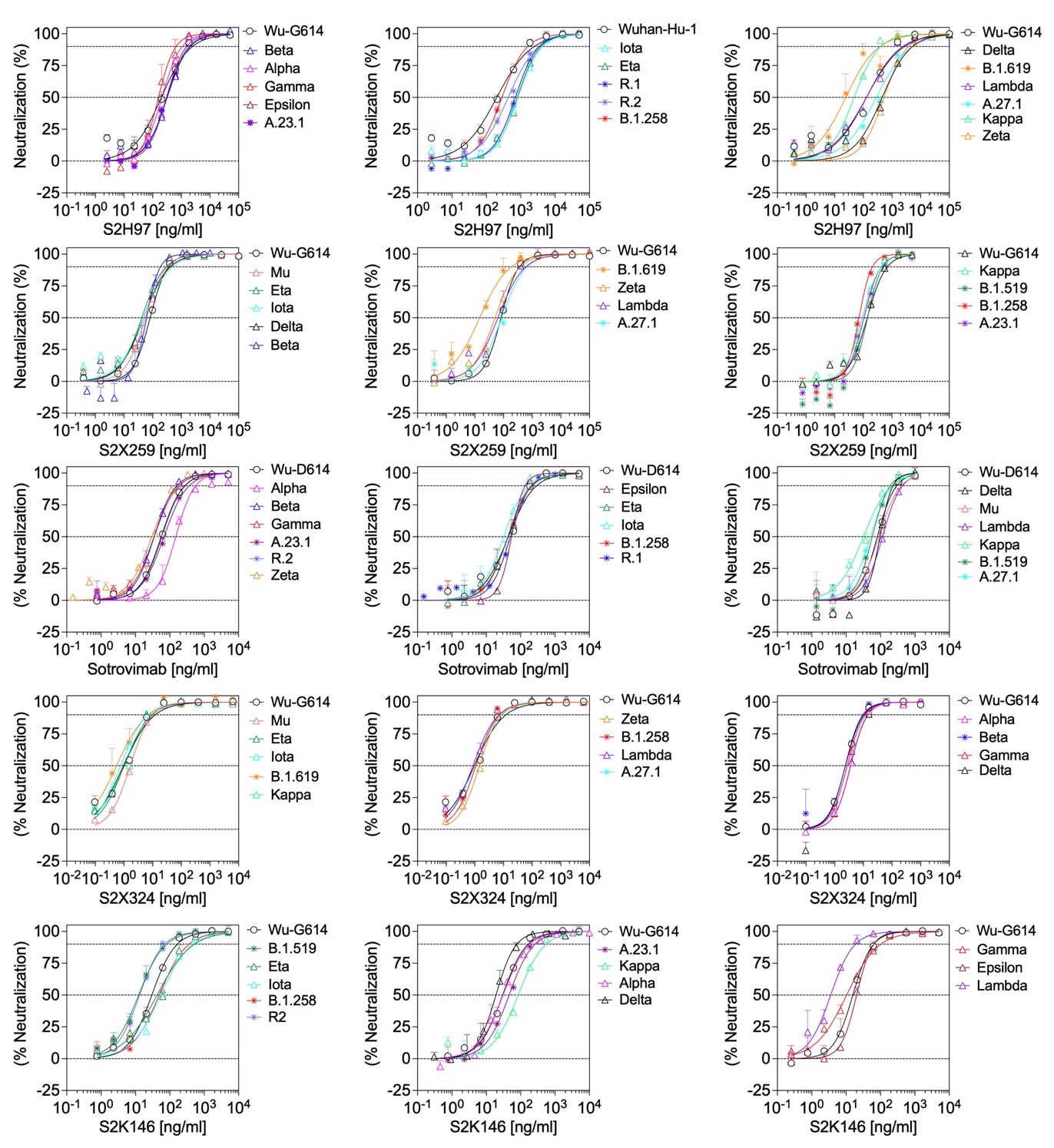
**

**Fig. S10C.** Neutralization of SARS-CoV-2 variant S VSV pseudoviruses mediated by broadly neutralizing sarbecovirus mAbs. One representative experiment out of at least two biological replicates is shown.

**Fig. S11.** **BA.2 mutations reducing sotrovimab binding to the BA.2 and related RBDs.** (**A-B**) Superimposition of the RBDs from the apo BA.2 S ectodomain trimer structure (gold, PDB 7UB0 (*45*)) onto the Omicron BA.1 S ectodomain trimer structure (cyan, PDB 7TLY (*18*)) bound to sotrovimab (purple and magenta for heavy and light chains) viewed along two orthogonal orientations. Only residues 361-382 of the BA.2 RBD are shown for clarity. The BA.1 S N343 glycan is shown as sticks and expected clashes with BA.2 residues N370 and F371 (spheres) are indicated with red stars in panel B. (**C-D**) Volumetric maps of the N343 glycan computed from molecular dynamics simulation of the BA.1 RBD (PDB 7TN0 (*18*)) mutated at positions 371, 376, 405, and 408 to match the BA.2 sequence (C) or from the BA.2 RBD (PDB ID 7BU0) (D) indicating steric overlap with the static sotrovimab heavy chain residues Y32 and Y100. (**E-F**) Neutralizing activity of sotrovimab against VSV pseudoviruses harboring SARS-CoV-2 Wu-G614 with the S371L/S373P/S375F (E) or the S371F/S373P/S375F (F) residue substitutions.


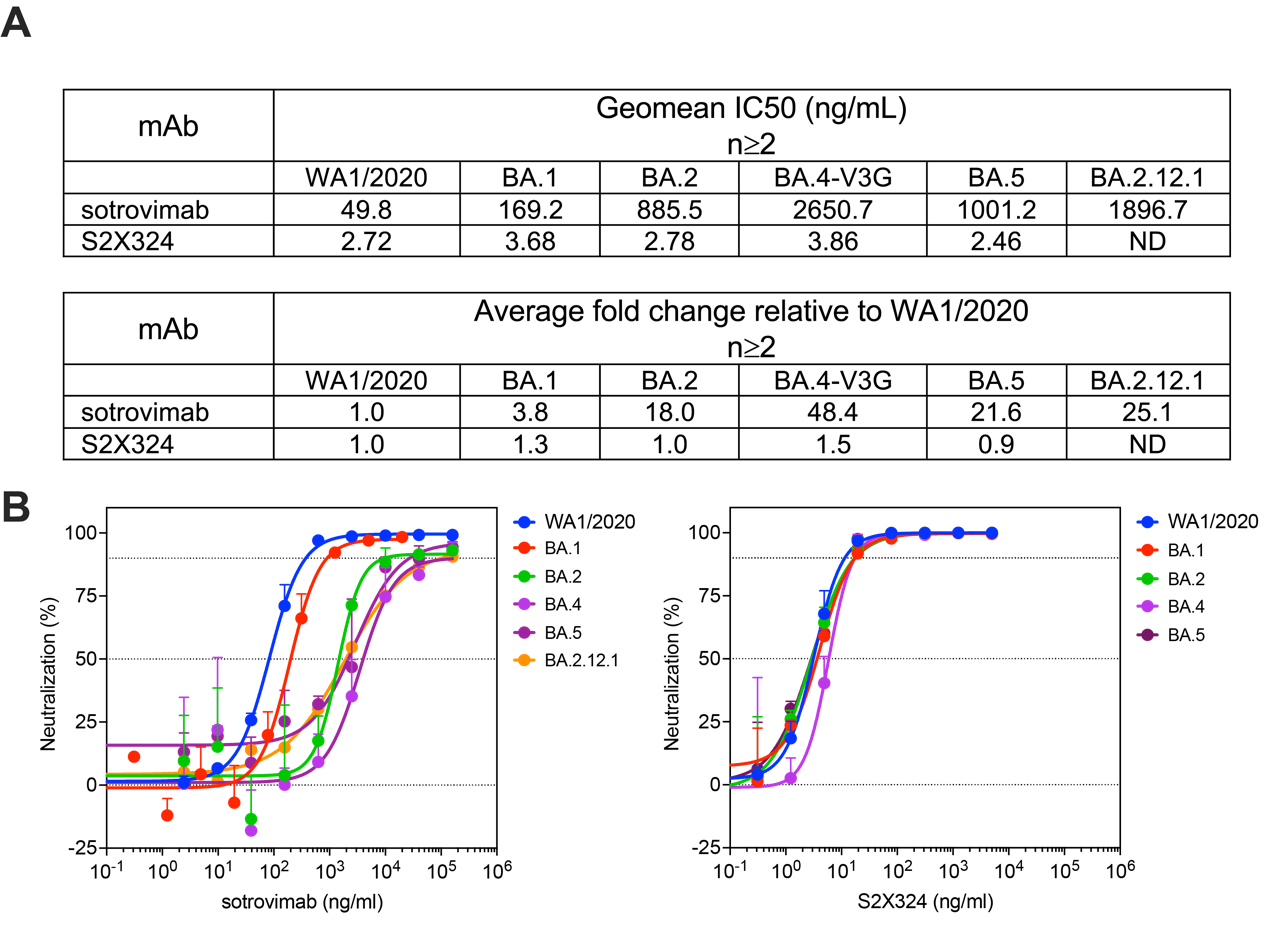


**Fig. S12.** **Neutralization of authentic SARS-CoV-2 variants by sotrovimab and S2X324.**

**A**, Geometric mean of IC_50_ values (ng/ml) and average fold change for the neutralization of Omicron sublineages and WA1/2020 by sotrovimab and S2X324 mAbs. Strains tested: USA-WA1/2020; BA.1: hCoV-19/USA/MD-HP20874/2021; BA.2: hCoV-19/USA/MD-HP24556/2022: BA.4: hCoV-19/USA/MD-HP30386/2022; BA.5: hCoV-19/USA/COR-22-063113/2022; BA.2.12.1: USA/NY-MSHSPSPPV56475/2022. The geometric mean IC_50_ reported was obtained from all independent experiments. The IC_50_ fold change for each authentic virus variant relative to the WA1/2020 virus was calculated for each experiment, and the mean fold change is reported. **B,** Representative curves showing neutralization of SARS-CoV-2 strains by sotrovimab and S2X324. Data represents the means of triplicates ± standard deviation from one experiment. Omicron BA.1 data are reported from (*43*). Graphs shown are representative of at least 2 independent experiments.

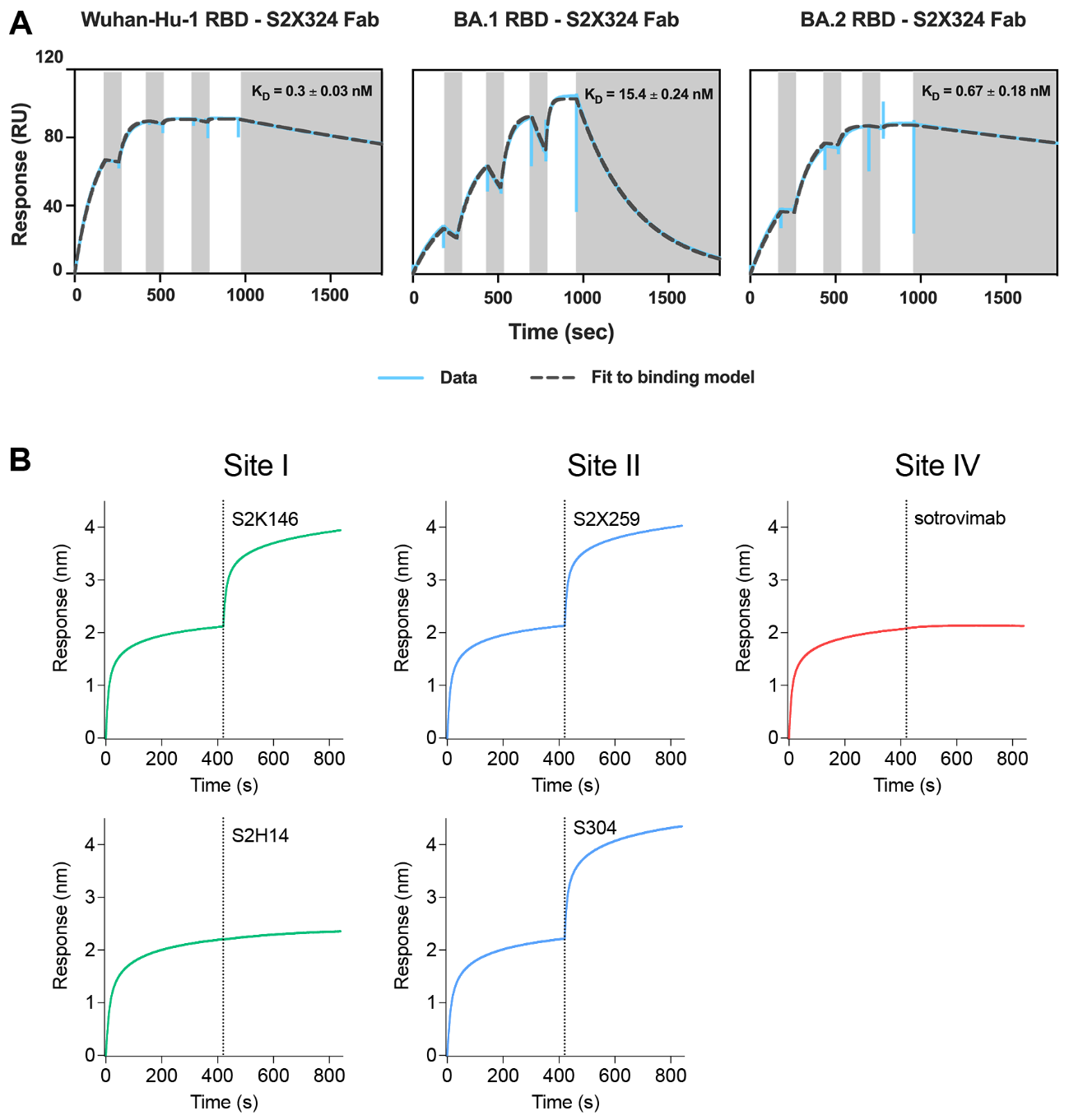


**Fig. S13.** **Binding affinity and site specificity of the S2X324 mAb** **for SARS-CoV-2 variant RBDs** (**A**) Binding of the S2X324 Fab to the SARS-CoV-2 Wuhan-Hu-1 RBD, BA.1 RBD or BA.2 RBD immobilized at the surface of SPR chips. Experiments were performed with a 3-fold dilution series of Fab (300, 100, 33 and 11 nM) and were run as single-cycle kinetics measurements. (**B**) Biolayer interferometry assessment of S2X324 mAb binding to the SARS-CoV-2 RBD in presence of site-I-targeting S2K146 or S2H14 mAbs; site-II targeting S2X259 or S304 mAbs; and site-IV-targeting S309 mAb. One independent experiment out of two is shown.


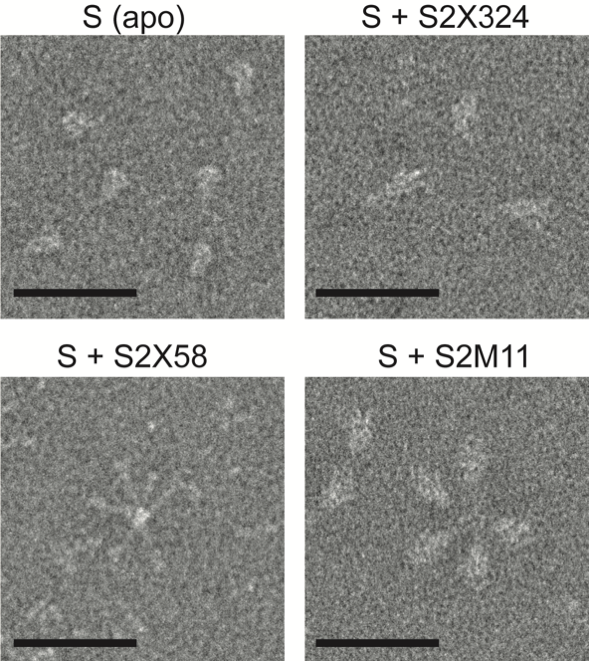


**Fig S14. Evaluation of the propensity of mAbs to promote S fusogenic conformational changes.**Fabs were incubated with native-like prefusion SARS-CoV-2 G614 S trimers for 1 hour and analyzed by negative stain EM to determine if the added Fabs promoted S refolding to the postfusion state.  Scale bar: 50 nm. Only S2X58 promoted S fusogenic conformational changes in these conditions.


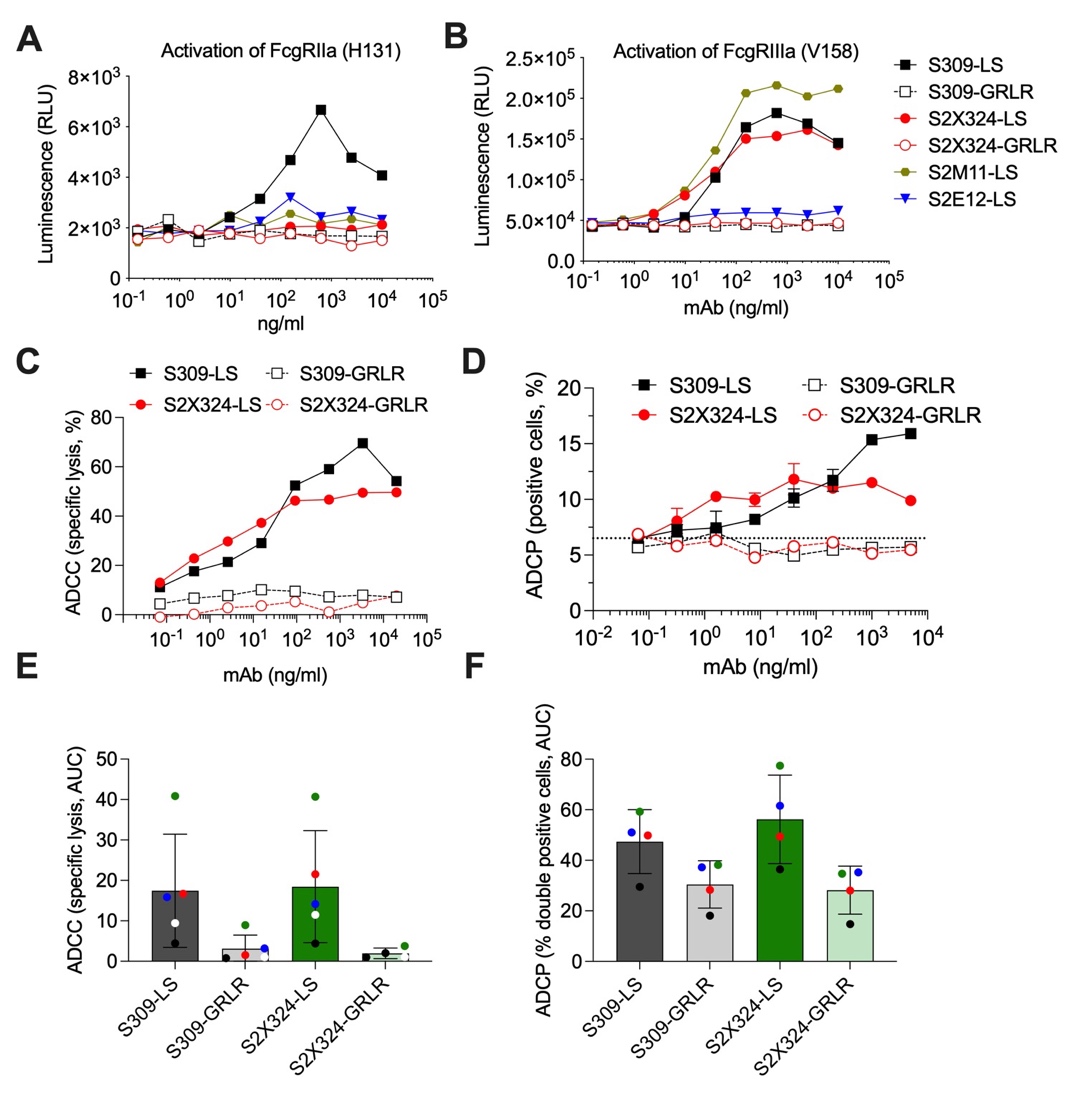


**Fig. S15. Analysis of S2X324-mediated effector functions. A-B**, NFAT-driven luciferase signal induced in Jurkat cells stably expressing FcγRIIa H131 (A) or FcγRIIIa V158 (B) by S2X324 binding to full-length wild-type SARS-CoV-2 S stably expressed at the surface of ExpiCHO cells. SE12, S2M11, S309 (sotrovimab parent), S309-GRLR, S2X324-GRLR mAbs are included as controls (GRLR: Fc-null mutations). **C-F,** S2X324-triggered activation of ADCC (C, E) and ADCP (D, F) following incubation with PBMCs as a source of NK cells and monocytes for ADCC and ADCP, respectively. AUC analyses of ADCC (E) and ADCP (F) mediated by S309-LS, S309-GRLR, S2X324-LS and S2X324-GRLR mAbs using primary NK cells or monocytes from five and four donors, respectively. Each donor is indicated by a given color.

**
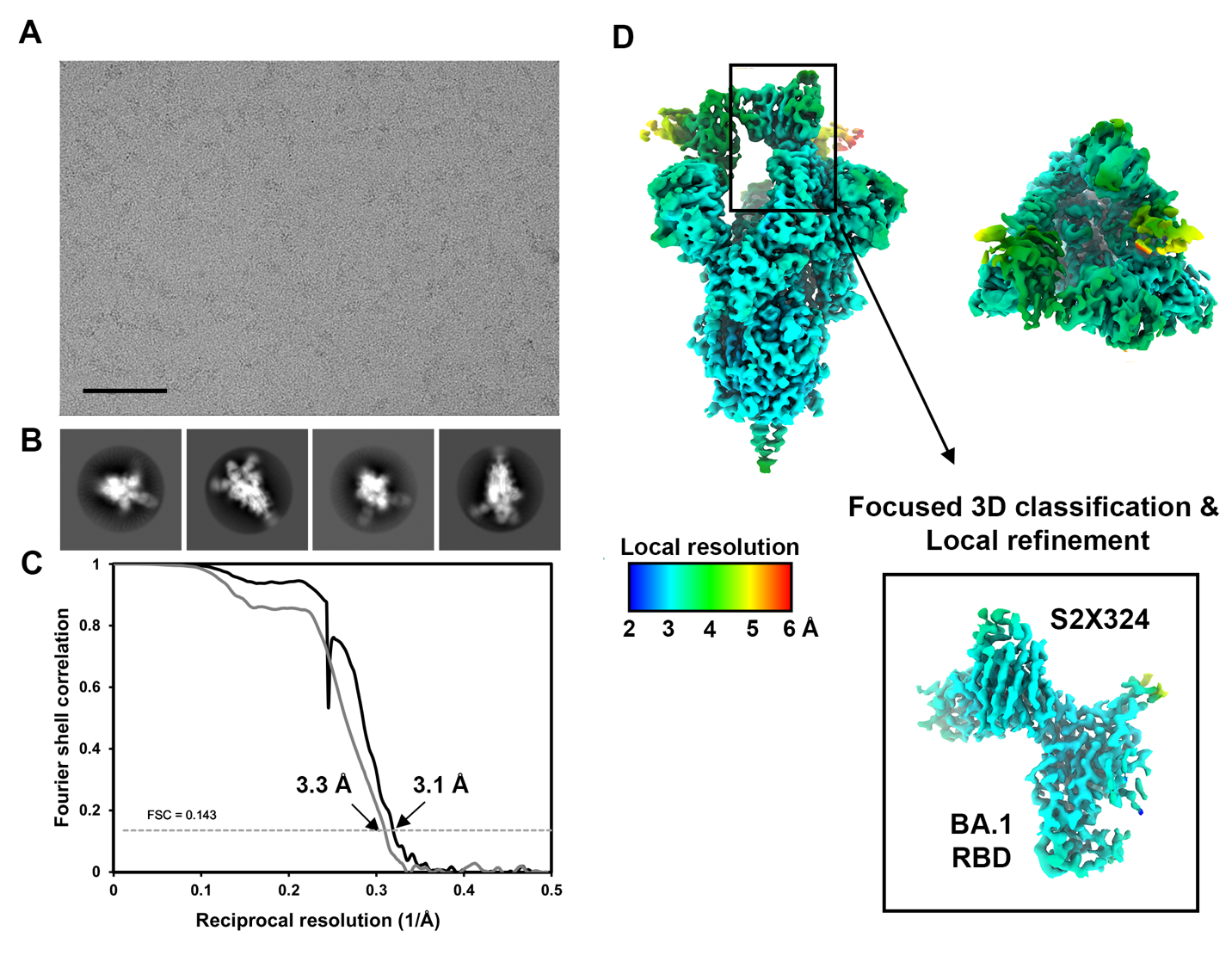
**

**Figure S16. CryoEM data processing of the S2X324-bound SARS-CoV-2 Omicron BA.1 S dataset.** **A-B,** Representative electron micrograph (A) and 2D class averages (B) of SARS-CoV-2 Omicron BA.1 S in complex with the S2X324 Fab embedded in vitreous ice. The scale bar represents 100 nm. **C,** Gold-standard Fourier shell correlation curves for the S2X324-bound SARS-CoV-2 S maps with two RBDs open state (black line) and locally refined RBD/S2X324 variable domain (grey line). The 0.143 cutoff is indicated by a horizontal dashed line. **D,** Local resolution maps calculated using CryoSPARC for the SARS-CoV-2 S/S2X324 Fab complex structure in two orthogonal orientations, the side view (left) and the top view (right) as well as for the locally refined RBD/S2X324 variable domain region (inset).


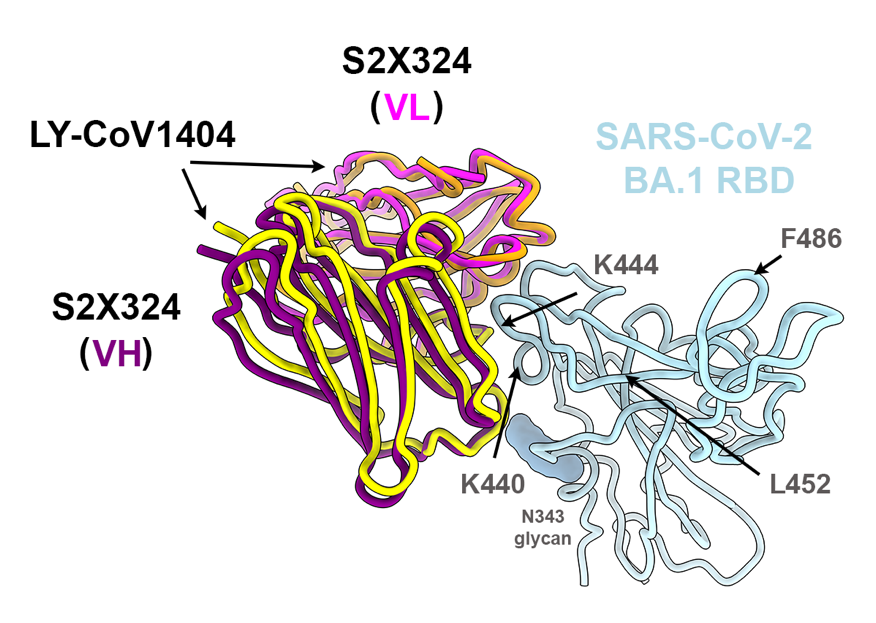


**Figure S17.** **Superposition of S2X324 and LY-CoV1404 bound to the SARS-CoV-2 RBD**.

Ribbon diagram of LY-CoV1404/RBD structure (PDB 7MMO, yellow and orange) superimposed onto S2X324/RBD (cryoEM structure, purple and magenta), using the RBD as a reference. The N343 glycan is rendered as blue spheres. Two selected epitope residues and the BA.5 RBD mutation relative to BA.2, i.e. residues L452R and F486V (which are positioned outside the S2X324/LY-CoV1404 epitope) are indicated with arrows.

**
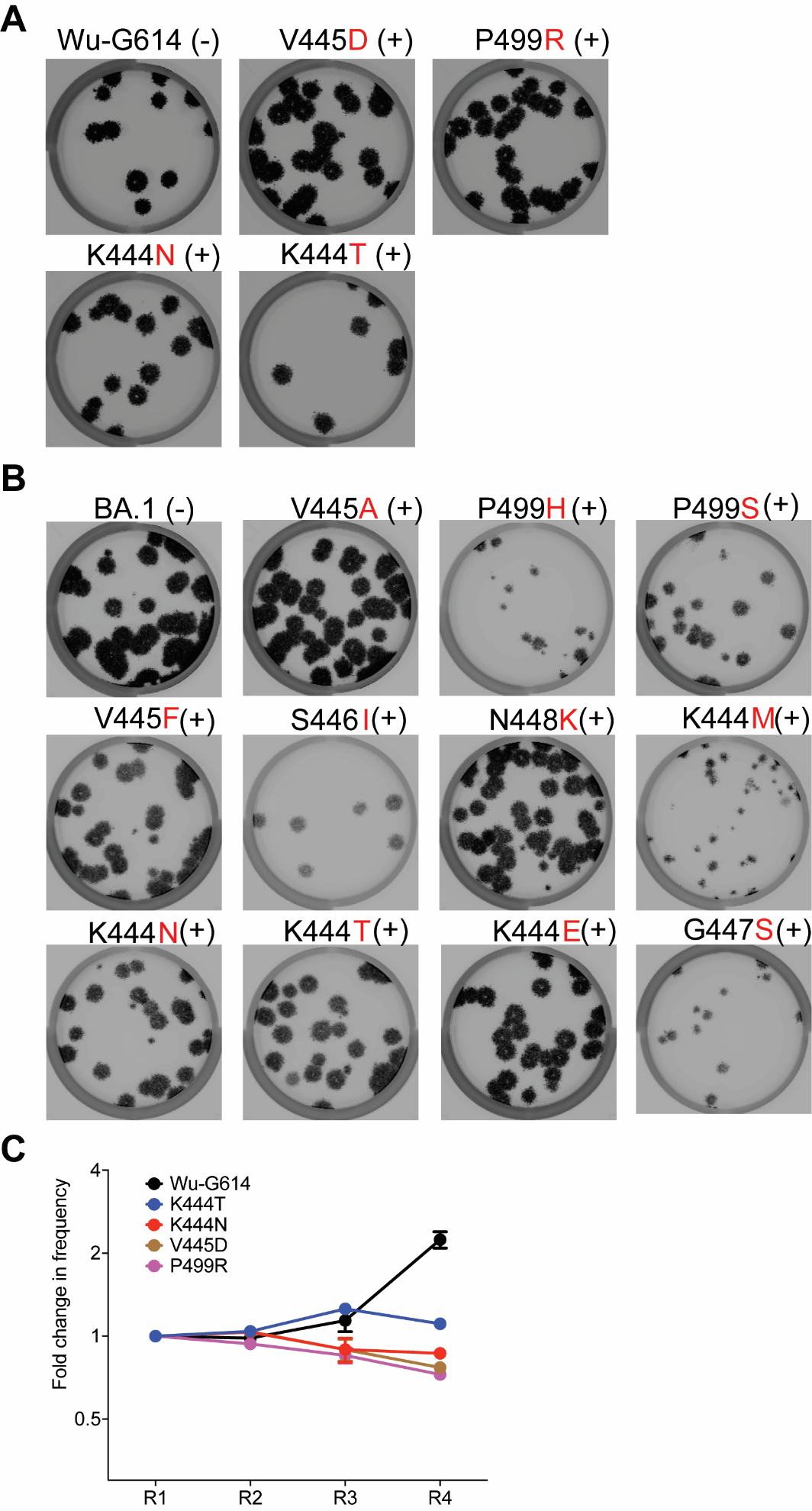
**

**Fig. S18. S2X324 escape clone selection by plaque assays using VSV/SARS-CoV-2 Wu-G614 or Omicron BA.1 S chimeric viruses. A-B,** Plaque assays performed to validate the isolated VSV/SARS-CoV-2 Wu-G614 S (A) and Omicron BA.1 (B) escape mutants using Vero cells in the presence (+) of S2X324 in the overlay. The escape plaques for each mutant are compared with those obtained with the corresponding wild type virus in absence (-) of S2X324 in the overlay. **C,** In vitro competition experiment to assess the relative replication fitness on VeroE6 cells of the VSV chimera harboring the SARS-CoV-2 Wu-G614 S in the absence or presence of one of the following S2X324 escape mutants: K444T/N, V445D and P499R.


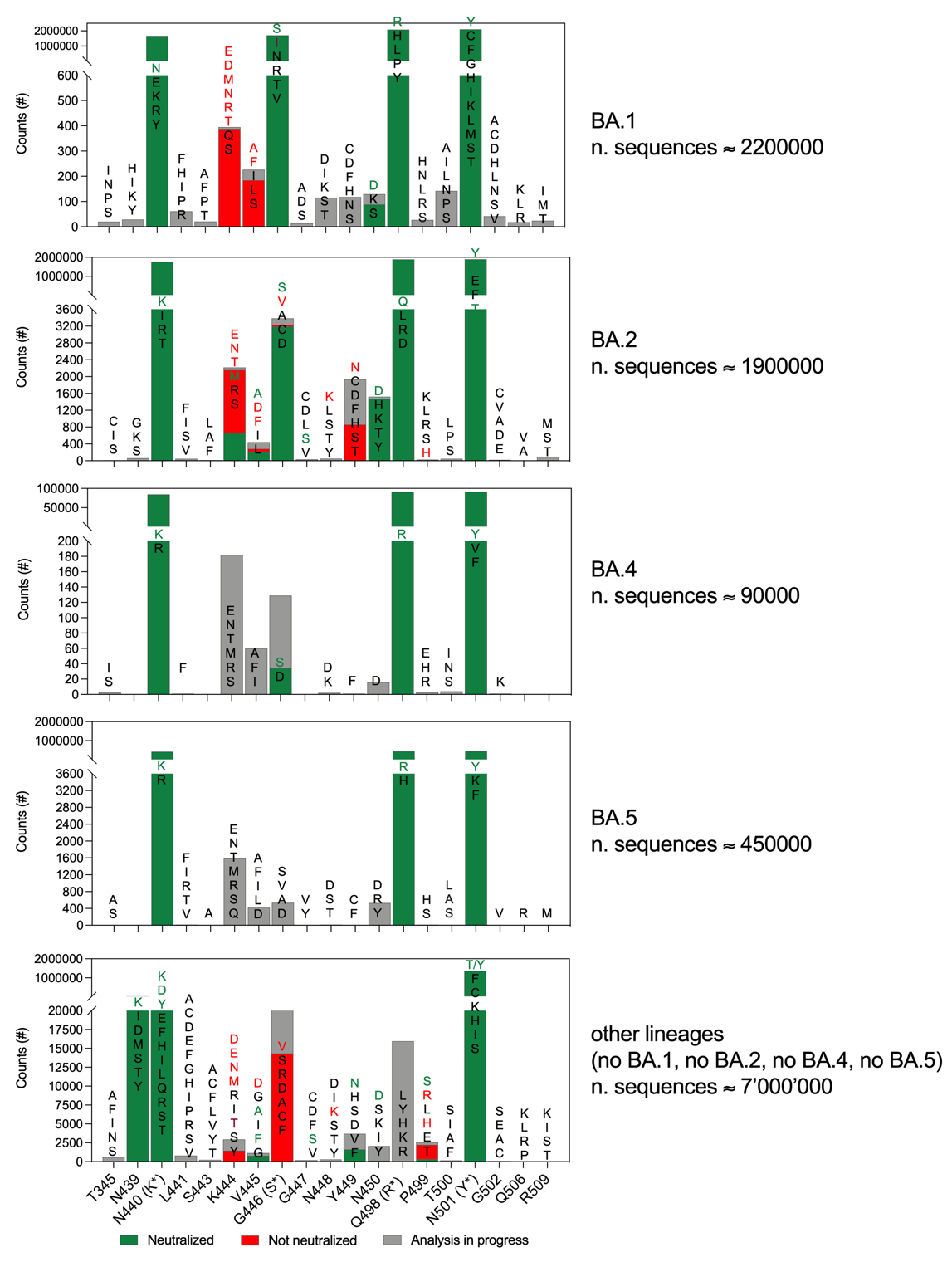


**Fig. S19.** **Frequency of observed mutants in the S2X324 epitope among deposited SARS-CoV-2 genomes.** Mutants within the S2X324 epitope on the basis of SARS-CoV-2 variant genome sequences available on GISAID as of 15 August 2022. Colored are shown the VSV pseudotyped mutants neutralized (green) or not neutralized (red) by S2X324.

**Data S1.** Description of the Cohorts of SARS-CoV-2-immune, vaccinated, or hybrid individuals, Related to Figure 1A, E-F.

**Table S1.** Neutralizing geometric mean titers (GMTs) against Wu-G614, Delta, BA.1, BA.2, BA.2.12.1, BA.4/5 and SARS-CoV S VSV pseudoviruses using plasma from subjects who were infected prior to being vaccinated with 2 doses (n=15) or 3 doses (n=8), subjects who experienced a Delta breakthrough infection after receiving 2 vaccine doses (n=15), a BA.1 breakthrough infection after either 2 (n=8) or 3 vaccine doses (n=8), or were vaccinated-only with 3 doses (n=7).

| GMTs | Infected-Vaccinated (2 doses) | Infected-Vaccinated (3 doses) | Delta Breakthrough (2 doses) | BA.1 Breakthrough (2 doses) | BA.1 Breakthrough (3 doses) | Vaccinated only (3 doses) |
| --- | --- | --- | --- | --- | --- | --- |
| Wu-G614 | 2065 | 6825 | 2031 | 3915 | 3757 | 1862 |
| Delta | 1398 | 1909 | 1033 | 2778 | 1927 | 662 |
| BA.1 | 576 | 830 | 560 | 2873 | 2393 | 426 |
| BA.2 | 1139 | 1708 | 792 | 2441 | 2340 | 531 |
| BA.2.12.1 | 981 | 1325 | 654 | 1446 | 2250 | 300 |
| BA.4/5 | 414 | 498 | 344 | 503 | 740 | 190 |
| SARS-CoV | 129 | 378 | 141 | 158 | 153 | 216 |

**Table S2.** Neutralizing activity against Wu-G614, Delta, BA.1, BA.2, BA.5 and SARS-CoV S VSV pseudoviruses using plasma from subjects who experienced an Omicron breakthrough or primary infection.

|  |  | Neutralization of VSV pseudoviruses harboring the indicated S  (reciprocal ID_50_) | | | | | | Neutralization of VSV pseudoviruses harboring the indicated S (fold loss vs Wu-G614) | | | | | | Fold loss of BA.5 vs BA.1 |
| --- | --- | --- | --- | --- | --- | --- | --- | --- | --- | --- | --- | --- | --- | --- |
|  | Donor ID | Wu | BA.1 | BA.2 | BA.5 | Delta | SARS-CoV | Wu | BA.1 | BA.2 | BA.5 | Delta | SARS-CoV | BA.5 |
| Vaccinated | VC201 | 1053 | 814 | 229 | 154 | 337 | 114 | 1 | 1 | 5 | 7 | 3 | 9 | 5 |
|  | VC202 | 4208 | 3827 | 2834 | 1467 | 2185 | 162 | 1 | 1 | 1 | 3 | 2 | 26 | 3 |
|  | VC203 | 4706 | 1150 | 1707 | 1416 | 2222 | 264 | 1 | 4 | 3 | 3 | 2 | 18 | 1 |
|  | VC204 | 7028 | 5804 | 2871 | 1085 | 4262 | 160 | 1 | 1 | 2 | 6 | 2 | 44 | 5 |
|  | VC206 | 6901 | 5904 | 2242 | 903 | 2510 | 100 | 1 | 1 | 3 | 8 | 3 | 69 | 7 |
|  | VC207 | 17538 | 1410 | 1533 | 916 | 19167 | 132 | 1 | 12 | 11 | 19 | 1 | 133 | 2 |
|  | VC208 | 6829 | 4333 | 2276 | 635 | 4401 | 126 | 1 | 2 | 3 | 11 | 2 | 54 | 7 |
|  | VC209 | 22780 | 7911 | 6322 | 2590 | 8192 | 368 | 1 | 3 | 4 | 9 | 3 | 62 | 3 |
| Non-vacc. | VC205 | 40 | 564 | 140 | 34 | 23 | 145 | 1 | 0 | 0 | 1 | 2 | 0 | 17 |
|  | VC210 | <10 | 20 | 9 | <10 | <10 | 31 | NC | NC | NC | NC | NC | NC | - |
|  | VC211 | 598 | 7294 | 1556 | 662 | 501 | 86 | 1 | 0 | 0 | 1 | 1 | 7 | 11 |

Shown is the reciprocal of the half-maximum inhibitory dose (ID_50_). NC, not calculable.

**Table S3.** Neutralizing activity of sotrovimab against VSV pseudoviruses harboring SARS-CoV-2 Wu-D614 S with the indicated substitutions.

| Amino Acid Substitutions | Geometric neutralization  (IC_50_, ng/ml) | Average fold-change in IC_50_ relative to wildtype^a^ |
| --- | --- | --- |
| S371L, S373P, S375F, D614G^b^ | 72.25 | 1.31 |
| S371F, S373P, S375F, D614G^c^ | 207.48 | 3.44 |

^a^ Fold change calculated relative to Wu-D614 (YP_009724390.1)

^b^ Evaluated in VeroE6 cells

^c^ Evaluated in VeroE6-TMPRSS2 cells due to decreased infectivity using VeroE6 cells.

**Table S4.** Kinetics parameters of S2X324 Fab binding to different RBDs evaluated from SPR binding assays with 2 replicates.

| **Replicate 1** | |  |  |  |  |  |  |  |
| --- | --- | --- | --- | --- | --- | --- | --- | --- |
| **RBD** | **ka (1/Ms)** | **kd (1/s)** | **KD (M)** | **KD (nM)** | **Rmax (RU)** | **tc** | **Chi² (RU²)** | **U-value** |
| WT | 6.92E+05 | 2.10E-04 | 3.04E-10 | 0.30 | 90.94 | 7.52E+14 | 0.175 | 1 |
| Omicron BA.1 | 1.89E+05 | 2.90E-03 | 1.54E-08 | 15.37 | 108.30 | 1.32E+16 | 1.56 | 1 |
| Omicron BA.2 | 2.79E+05 | 1.52E-04 | 5.43E-10 | 0.54 | 87.88 | 3.57E+16 | 1.62 | 2 |

| **Replicate 2** | |  |  |  |  |  |  |  |
| --- | --- | --- | --- | --- | --- | --- | --- | --- |
| **RBD** | **ka (1/Ms)** | **kd (1/s)** | **KD (M)** | **KD (nM)** | **Rmax (RU)** | **tc** | **Chi² (RU²)** | **U-value** |
| WT | 6.80E+05 | 2.31E-04 | 3.39E-10 | 0.34 | 85.04 | 6.41E+14 | 0.676 | 1 |
| BA.1 | 1.78E+05 | 0.002789 | 1.57E-08 | 15.71 | 129.6 | 1.43E+09 | 1.32 | 1 |
| BA.2 | 2.52E+05 | 2.00E-04 | 7.94E-10 | 0.79 | 63.87 | 3.12E+16 | 1.45 | 2 |

**Table S5. CryoEM data collection and refinement statistics.**

|  | SARS-CoV-2 S/S2X324  PDB 8ERR  EMD-28559 | SARS-CoV-2 S/S2x324  (local refinement)  PDB 8ERQ  EMD-28558 |
| --- | --- | --- |
| **Data collection and processing** |  |  |
| Magnification | 105,000 | 105,000 |
| Voltage (kV) | 300 | 300 |
| Electron exposure (e^–^/Å^2^) | 60 | 60 |
| Defocus range (μm) | 0.5-2.5 | 0.5-2.5 |
| Pixel size (Å) | 0.843 | 0.843 |
| Symmetry imposed | C1 | C1 |
| Final particle images (no.) | 398,180 | 354,249 |
| Map resolution (Å) | 3.1 | 3.3 |
| FSC threshold | 0.143 | 0.143 |
| Map sharpening *B* factor (Å^2^) | -98 | -100 |
|  |  |  |
| **Validation** |  |  |
| MolProbity score | 1.22 | 0.99 |
| Clashscore | 2.33 | 0.61 |
| Poor rotamers (%) | 0.88 | 1.09 |
| Ramachandran plot |  |  |
| Favored (%) | 96.7 | 96.2 |
| Allowed (%) | 3.0 | 3.8 |
| Disallowed (%) | 0.3 | 0 |

| **mAb** | **Clones** | **Nucleotide**  **mutant** | **Amino acid**  **mutant** |
| --- | --- | --- | --- |
| **S2X324** | #1 | G1332T | K444N |
|  | #2 | G1332T | K444N |
|  | #3 | T133AT | V445D |
|  | #4 | C1496G | P499R |
|  | #5 | G1332T | K444N |
|  | #6 | A1331C | K444T |

**Table S6.** Summary of nucleotide and amino acid mutations found in S2X324 neutralization-resistant VSV/SARS-CoV-2 Wu-G614 S chimera plaques.

| **mAbs** | **Clones** | **Nucleotide**  **mutant** | **Amino acid**  **mutant** |
| --- | --- | --- | --- |
| **S2X324** | #1 | G1332T | K444N |
|  | #2 | A1330G | K444E |
|  | #3 | G1332T | K444N |
|  | #4 | C1495T | P499S |
|  | #5 | G1332T | K444N |
|  | #6 | C1495T | P499S |
|  | #7 | T1334C | V445A |
|  | #8 | G1332T | K444N |
|  | #9 | C1496A | P499H |
|  | #10 | A1072T/  T1344C | I358F/  N448K |
|  | #11 | C1495T | P499S |
|  | #12 | A1330G | K444E |
|  | #13 | G1333T | V445F |
|  | #14 | G1337T | G446S S446I |
|  | #15 | G1332T | K444N |
|  | #16 | C1495T | P499S |
|  | #17 | T1344C | N448K |
|  | #18 | A1331T | N444M |
|  | #19 | G1332T | K444N |
|  | #20 | A1331C | K444T |
|  | #21 | A1331C | K444T |
|  | #22 | A1330G | K444E |
|  | #23 | G1339A | G447S |
|  | #24 | G1332T | K444N |

**Table S7.** Summary of nucleotide and amino acid mutations found in S2X324 neutralization-resistant VSV/SARS-CoV-2 Omicron BA.1 S chimera plaques.

|  |  | **VSV chimera** | **BA.1*** | **BA.2*** | **BA.4*** | **BA.5*** | **Other*** |
| --- | --- | --- | --- | --- | --- | --- | --- |
| **K444** | N | Wuhan/BA.1 | 72 | 1575 | 63 | 788 | 1292 |
|  | T | Wuhan/BA.1 | 185 | 36 | 18 | 449 | 125 |
|  | E | Wuhan/BA.1 | 11 | 10 | 3 | 9 | 31 |
|  | M | BA.1 | 8 | 14 | 10 | 234 | 186 |
|  | R | / | 113 | 659 | 107 | 535 | 1207 |
|  | D | Wuhan | 0 | 1 | 0 | 0 | 2 |
| **V445** | A | BA.1 | 142 | 228 | 47 | 383 | 443 |
|  | F | BA.1 | 41 | 56 | 18 | 121 | 336 |
|  | D | Wuhan | 0 | 10 | 0 | 3 | 8 |
|  | T | / | 0 | 0 | 0 | 0 | 0 |
| **S446** | I | BA.1 | 25 | 2 | 0 | 0 | 0 |
| **G447** | S | BA.1 | 5 | 10 | 0 | 0 | 72 |
| **N448** | K | BA.1 | 4 | 3 | 1 | 0 | 36 |
| **Y449** | N | / | 13 | 857 | 0 | 0 | 1622 |
| **P499** | H | BA.1 | 0 | 2 | 2 | 1 | 110 |
|  | R | Wuhan | 11 | 4 | 1 | 1 | 1772 |
|  | S | BA.1 | 6 | 4 | 0 | 15 | 340 |
|  |  | Sum | 636 | 3471 | 270 | 2539 | 7582 |
|  |  | Total | 2,283,189 | 2,031,048 | 98180 | 515541 | 7,078,434 |
|  |  | % | 0.03 | 0.17 | 0.28 | 0.49 | 0.11 |

*Counts retrieved from GISAID as of August 18, 2022, (sequences with Ns>5% were excluded from analysis)

**Table S8.** Evaluation of neutralizing activity of S2X324 against VSV pseudoviruses harboring S mutants and their prevalence among Delta and Omicron isolates.

| Amino acid residue | Mutation | Geomean IC_50_ (ng/mL)  n=2 | | | Average fold change relative  to reference variant  n=2 | | |
| --- | --- | --- | --- | --- | --- | --- | --- |
|  |  | S backbone | | | S backbone | | |
|  |  | Wu-G614 | BA.1 | BA.2 | Wu-G614 | BA.1 | BA.2 |
| N439 | K | 1.4 | nd | nd | 1.3 | nd | nd |
| N440(K*) | Y | 1.6 | nd | 4 | 1.3 | nd | 0.8 |
|  | D | 3.0 | nd | 5.7 | 2.5 | nd | 1.2 |
|  | N* | nd | 3 | 3.6 | nd | 0.9 | 1.0 |
|  | K | 1.5 | nd | nd | 1.2 | nd | nd |
| K444 | E | >2500 | >2500 | >2500 | - | - | - |
|  | D | >2500 | >2500 | >2500 | - | - | - |
|  | N | >2500 | >2500 | >2500 | - | - | - |
|  | M | nd | >2500 | 47.9 | nd | - | 12.4 |
|  | T | >2500 | >2500 | >2500 | - | - | - |
|  | R | nd | 56.4 | 5.1 | nd | 18 | 1.5 |
| V445 | D | >2500 | >2500 | >2500 | - | - | - |
|  | T | 17.1 | >2500 | 33.7 | 13.8 | - | 8.7 |
|  | A | 26.9 | >2500 | 43.1 | 5.2 | - | 11 |
|  | F | 8.4 | >2500 | 267 | 1.6 | - | 52.3 |
| G446(S*) | V | 27.9 | nd | >2500 | 21.9 | nd | - |
|  | I | 353.5 | nd | >2500 | 71.9 | nd | - |
|  | G* | nd | 2.5 | nd | nd | 0.7 | nd |
|  | S | nd | nd | 6 | nd | nd | 1.7 |
| G447 | S | 7.5 | nd | 26.2 | 1.4 | nd | 4.8 |
| N448 | K | >2500 | nd | >2500 | 742.5 | nd | - |
| Y449 | N | 1.2 | nd | >2500 | 0.9 | nd | - |
| N450 | D | 4.4 | 7 | 11.6 | 4.8 | 2 | 2.5 |
| Q498 (R*) | Q* | nd | 18.1 | 3.9 | nd | 5.3 | 1.1 |
| P499 | S | 7.8 | nd | 67.6 | 6.1 | nd | 12.2 |
|  | H | 714.2 | nd | >2500 | 236.5 | nd | - |
|  | R | >2500 | nd | nd | 805.2 | nd | nd |
| N501(Y*) | Y | 2 | nd | nd | 1.5 | nd | nd |
|  | T | 2.8 | nd | 2.8 | 2.2 | nd | 0.6 |
|  | N* | nd | 4.3 | nd | nd | 1.1 | nd |
| V503 | I | 1.5 | nd | 4.5 | 1.1 | nd | 1 |

**Table S9.** Neutralization of VSV pseudoviruses harboring the SARS-CoV-2 Wu-D614, BA.1 or BA.2 S mutations within the S2X324 epitope reported as geometric mean titers obtained from two technical replicates (IC_50_ expressed in ng/mL) and average fold change relative to the reference variant. “nd” not determined; “*” amino acid residue substitutions found in the Omicron variants.
